# Crystalline Ni_3_S_2_ Nanorods Tuned by Low-Crystalline NiCoS_*x*_ with Built-In Electric Field for Efficient Overall Water Splitting

**DOI:** 10.1007/s40820-026-02151-6

**Published:** 2026-03-26

**Authors:** Neng Chen, Jun He, Hongqiang Li, Dedong Jia, Haoran Qian, Huan Pang, Jieshan Qiu, Yongfeng Li, Xiaojun He

**Affiliations:** 1 School of Chemistry and Chemical Engineering, Anhui Province Key Laboratory of Coal Clean Conversion and Low Carbon Utilization, Anhui Province Key Laboratory of Efficient Conversion and Solid-State Storage of Hydrogen & Electricity, Key Laboratory of Metallurgical Emission Reduction and Resources Recycling, Ministry of Education, Anhui University of Technology, Ma’anshan, Anhui, 243002 People’s Republic of China; 2 Institute of Innovation Materials and Energy, School of Chemistry and Chemical Engineering, Yangzhou University, Yangzhou, 225002 People’s Republic of China; 3 College of Chemical Engineering, State Key Laboratory of Chemical Resource Engineering, Beijing University of Chemical Technology, Beijing, 100029 People’s Republic of China; 4 State Key Laboratory of Heavy Oil Processing, China University of Petroleum, Beijing, 102249 People’s Republic of China

**Keywords:** NiCoS_*x*_@Ni_3_S_2_, Low-crystalline tuned crystalline heterostructure, Built-in electric field, Work function, Water splitting

## Abstract

Efficient overall water splitting (OWS) technology has been highly demanded across the world, of which the key is the development of active and stable electrocatalysts for both hydrogen and oxygen evolution reactions (HER/OER). Herein, novel crystalline Ni_3_S_2_ nanorods tuned by low-crystalline NiCoS_*x*_ (NiCoS_*x*_@Ni_3_S_2_) are synthesized via an ion-exchange strategy. The built-in electric field at the heterogeneous interface driven by work function difference, facilitates rapid electron transfer from Ni_3_S_2_ to NiCoS_*x*_ via a robust Ni–S–Co bond bridge. This synergistic combination of the conductive crystalline core and low-crystalline shell optimizes the d-band center, balancing intermediate adsorption/desorption, speeding up water dissociation, enhancing hydrogen adsorption/desorption for HER, and lowering the energy barrier for OER, ultimately boosting OWS efficiency. The defect-rich, low-crystalline NiCoS*x* shell, bonded to the crystalline core via Ni–S–Co bonds, serves as a protective armor, enabling dynamic reconstruction into NiCoOOH and suppresses sulfide leaching, ensuring catalytic stability. The optimized NiCoS_*x*_@Ni_3_S_2_ achieves low HER/OER overpotentials of 346/520 mV@1000 mA cm^−2^, evidenced by an ultralow cell voltage of 2.10 V@1000 mA cm^−2^ for OWS and long-term durability up to 400 h. The work paves a novel way to fabricate sulfur-based electrocatalysts with high yet balanced activity and stability for OWS via an interface engineering strategy.

**Supplementary Information:**

The online version contains supplementary material available at 10.1007/s40820-026-02151-6.

## Highlights

- Low-crystalline NiCoS_x_-armored Ni_3_S_2_ nanorod heterostructure inhibits sulfide loss.
- Built-in electric field effect induced by work function accelerates electron transfer.
- The NiCoS_x_@Ni_3_S_2_/NF exhibits superior hydrogen and oxygen evolution reactions activity and stability.

**Supplementary Information:**

The online version contains supplementary material available at 10.1007/s40820-026-02151-6.

## Graphical abstract

**Figure:**
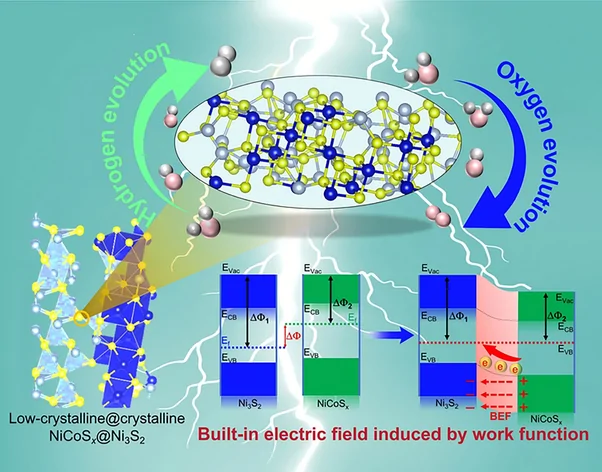

## Introduction

Hydrogen has emerged as one of the most promising clean energy carriers, providing a sustainable alternative to conventional fossil fuels due to its exceptional energy density and zero-carbon emissions in the global transition to a low-carbon economy [1, 2]. Electrochemical overall water splitting (OWS), which involves hydrogen evolution reaction (HER) and oxygen evolution reaction (OER), is considered one of the most promising technologies for efficient and sustainable hydrogen production [3, 4]. However, the large-scale industrial application of OWS is hampered by inherent limitations of state-of-the-art transition metal-based electrocatalysts including industrial-grade current densities, overpotential surges drastically due to insufficient active sites, sluggish mass/charge transfer, and mismatched intermediate adsorption energies [5, 6]. Harsh oxidative/reductive electrolytic environments trigger active phase exfoliation, lattice strain, and surface corrosion, with even cutting-edge catalysts barely stable for over 100 h at 1000 mA cm⁻^2^. Cost-efficiency is compromised by high cost/scarcity of noble metals and the complex synthesis process/poor stability of non-noble transition metal-based alternatives [7–9]. Therefore, it is urgent to develop transition metal-based electrocatalysts with balanced activity and stability for OWS [10, 11].

In the last few years, transition metal compounds, including transition metal oxides [12], phosphides [13], sulfides (TMSs) [14], nitrides [15], have undergone extensive research due to their availability and cost-efficiency. Among them, transition metal sulfides (TMSs, such as M_3_S_2_ and M_9_S_8_) exhibit excellent catalytic potential in HER and OER reactions, attributing to their unique d-electron configurations and adjustable sulfur coordination environments [16, 17]. Sulfur doping, in particular, is able to significantly modulate the electronic structure by adjusting electron density to optimize intermediate adsorption and charge transfer processes [18]. Sulfur doping or metal substitution can optimize the electronic structure and adjust the adsorption free energies of intermediates (H*, *OOH), thus enhancing catalytic activity [19, 20]. Furthermore, advanced interface engineering approaches, particularly the construction of well-designed heterostructures, have shown advantages in optimizing interfacial charge distribution and boosting catalytic performance [21, 22]. At present, it is necessary to establish a bi-directional regulation strategy in terms of O_2_/H_2_ intermediate binding energies to optimize the catalyst performance.

In recent years, low-crystallinity materials have gained widespread attention in the field of catalysis due to their atomic disorder, high density of dangling bonds, and abundant lattice defects. These structural features provide two core advantages for low-crystallinity materials: (1) high surface activity: amorphous/low-crystal surfaces expose more unsaturated coordination sites to optimize the adsorption of reaction intermediates; (2) excellent stability: disordered structures mitigate lattice strain at high potentials, preventing catalyst dissolution or phase transitions [23, 24]. Therefore, low-crystallinity materials are often regarded as a “low-crystal/amorphous skin” to offer more active sites and enhanced material stability [25, 26]. The long-range disorder of low-crystallinity materials leads to low charge carrier mobility, severely limiting electronic transport efficiency and thereby affecting catalytic kinetics [27, 28]. Yet, the conductivity of low-crystallinity materials is insufficient to support efficient catalysis. Thus, enhancing charge carrier mobility while maintaining surface activity remains a critical challenge.

To address this issue, researchers have proposed a gradient structural design strategy, wherein a long-range ordered crystalline framework (e.g., carbon coating or metal support) is constructed within the core of low-crystallinity materials to improve electronic conductivity, while the outer layer retains the high-activity defect structure, maintaining surface activity while enhancing conductivity [29, 30]. Despite progress in this area, the structural reconstruction of low-crystallinity/crystalline interfaces and the deactivation of active sites at high potentials and currents remain unresolved issues [31, 32]. Recent research has revealed that the built-in electric field (BEF) induced by the work function difference (ΔΦ-induced BEF) can significantly optimize catalytic behavior at high potentials and currents. ΔΦ-induced BEF drives charge redistribution at the interface, optimizing the adsorption of intermediates (such as H_2_/O_2_), thus improving catalytic efficiency [33, 34]. For example, Zhang et al. [35] utilized ΔΦ-induced BEF in the Ni_3_S_2_/Co_3_O_4_ heterostructure to enhance urea molecule adsorption, improving electrooxidation efficiency. BEF is used to modulate the hydrogen adsorption free energy in the Ni_2_P–CoCH sulfide heterostructure, reducing the HER overpotentials [36]. However, most current studies focus on BEF mechanisms at crystalline heterojunctions, and the BEF formation mechanism at low-crystallinity/crystalline interfaces remains unclear [37, 38]. Moreover, at high potentials and currents, structural reconstruction and active site deactivation at low-crystallinity/crystalline interfaces have yet to be fully resolved [39–42]. Hence, it is a challenge to achieve engineering of low-crystalline/crystalline heterogeneous interface with ΔΦ-induced BEF to develop OWS electrocatalysts with high yet balanced activity and stability. In all, the scale-up of OWS technology for industrial applications is plagued by specific bottlenecks including performance decay at high current densities, poor durability in harsh electrolytic environments, and cost-effectiveness imbalance [43–45].

Herein, we report crystalline Ni_3_S_2_ nanorods tuned by low-crystalline NiCoS_*x*_ (NiCoS_*x*_@Ni_3_S_2_) by an anion and cation exchange strategy. The ultraviolet photoelectron spectroscopy (UPS) spectra, X-ray absorption near edge structure (XANES) spectra and density functional theory (DFT) calculations reveal that the ΔΦ-induced BEF at NiCoS_*x*_/Ni_3_S_2_ heterogeneous interface facilitates electron transfer through Ni–S–Co interfacial bridge, enhancing water dissociation and optimizing hydrogen adsorption/desorption for HER. Meanwhile, the BEF in NiCoS_*x*_@Ni_3_S_2_ reduces the reaction energy barrier of *O → *OOH for OER, thereby promoting the overall catalytic kinetics of water splitting. In situ Raman and X-ray photoelectron spectroscopy (XPS) characterization results illustrate that the ultra-thin NiCoS_*x*_ layer functions as a protective armor to avoid or suppress the sulfide loss, resulting in high catalytic activity and stability of the NiCoS_*x*_@Ni_3_S_2_ catalysts. The NiCoS_*x*_@Ni_3_S_2_ with tuned structure makes the HER/OER possible at a low overpotential of 346/520 mV@1000 mA cm^−2^, evidenced by an ultralow cell voltage of 2.10 V@1000 mA cm^−2^ for OWS and long-term durability up to 400 h at current densities of 1000 mA cm^−2^, outperforming most of the TMS-based electrocatalysts in literature. The work paves a way to fabricate sulfur-based electrocatalysts with high yet balanced activity and stability for OWS via interface engineering strategy.

## Experimental Section

### Synthesis of Ni(OH)_2_/NF

To remove the oxide layer, Ni foam (NF, 2 cm × 2 cm) was sonicated in ethanol, acetone, and HCl solution for 15 min, respectively. Subsequently, the NF was soaked in deionized (DI) water at 80 °C for 3 h to grow Ni(OH)_2_ nanosheets on NF surface. The resulting samples were repeatedly washed with ethanol and air-dried at room temperature. The sample was labeled as Ni(OH)_2_/NF.

### Synthesis of NiCo_2_O_4_@NiO/NF

To investigate the regulation effects of temperature and time on the cation exchange process, the as-prepared Ni(OH)_2_/NF was immersed into 0.1 M Co(NO_3_)_2_ aqueous solution under different reaction conditions: varying temperatures (40, 80, 120, and 160 °C) for 3 h, and varying durations (0.5, 1, 3, and 6 h) at the temperature of 120 °C. After the cation exchange reaction, the sample was taken out and dried naturally at room temperature to form Ni/Co(OH)_2_/NF nanosheet arrays. The as-prepared Ni/Co(OH)_2_/NF was oxidized in a tube furnace under air atmosphere at 350 °C for 2 h to obtain NiCo_2_O_4_@NiO/NF nanosheets.

### Synthesis of NiCoS_x_@Ni_3_S_2_/NF

Analogous to the cation exchange regulation, the anion exchange sulfidation process was also optimized by adjusting temperature and time. Specifically, the as-prepared NiCo_2_O_4_@NiO/NF was vertically erected in 30 mL of 50 mmol L^−1^ thiourea solution, and the mixture was transferred into a 100 mL Teflon-lined autoclave. The autoclave was sealed and maintained under different reaction conditions: varying temperatures (80, 120, 160, and 200 °C) with a fixed duration of 3 h, and varying durations (0.5, 1, 3, and 6 h) at a fixed temperature of 160 °C. After the anion exchange sulfidation reaction, the autoclave was naturally cooled to room temperature. The resulting electrode material was thoroughly washed with ethanol and DI water to remove unreacted thiourea and by-products, then dried naturally at room temperature. The final product, composed of crystalline Ni_3_S_2_ nanorods tuned by low-crystalline NiCoS_*x*_, was denoted as NiCoS_*x*_@Ni_3_S_2_/NF.

### Synthesis of Ni_3_S_2_/NF and NiCoS_x_/NF

Ni_3_S_2_/NF was synthesized by the same procedure with the absence of Co(NO_3_)_2_. NiCoS_*x*_/NF was synthesized by the same procedure with the presence of Ni(OH)_2_/NF in 0.1 M Co(NO_3_)_2_ solution at 80 °C for 12 h for sufficient cation exchange and was oxidized in a tube furnace to obtain NiCo_2_O_4_/NF. The NiCo_2_O_4_/NF was etched using thiourea at 160 °C for 12 h for sufficient anion exchange.

## Results and Discussion

### Synthesis and Characterization of Low-Crystalline NiCoS_***x***_-Tuned Crystalline Ni_3_S_2_

As illustrated in Fig. S1, the crystalline Ni_3_S_2_ nanorods tuned by low-crystalline NiCoS_*x*_ were synthesized via three-step procedure, e.g., in situ growth, anion and cation exchange, in which nickel foam (NF) serves as the conductive substrate and nickel source. First, Ni(OH)_2_/NF was prepared in situ on nickel foam at 80 °C in deionized water, where NF undergoes spontaneous surface oxidation, generating Ni^2+^, OH^−^, and hydrogen gas. This electrochemical corrosion process is shown as: 2Ni + 2H_2_O + O_2_ → 2Ni(OH)_2_. When the Ni^2+^ and OH^−^ reach a high concentration near the NF surface, they form Ni(OH)_2_ and deposit back onto the surface. Subsequently, Ni/Co(OH)_2_/NF was formed by dipping Ni(OH)_2_/NF into Co(NO_3_)_2_ solution via cation exchange. In solution, Co^2+^ first adsorbs onto the negatively charged Ni(OH)_2_ surface through electrostatic interactions. An ion exchange reaction then occurs: Ni(OH)_2_ + Co^2+^ → NiCo(OH)_*x*_, which promotes the entry of Co^2+^ into the lattice, replacing Ni^2+^ in Ni(OH)_2_ to form a NiCo(OH)_*x*_ solid solution. At 300 °C in air, NiCo(OH)_*x*_ undergoes dehydration and transforms into NiCo_2_O_4_: NiCo(OH)_2_ → NiCo_2_O_4_ + H_2_O. Metal and oxygen ions move and rearrange during this process. At the same time, some Co^2+^ and Ni^2+^ are oxidized into Co^3+^ and Ni^3+^, forming a mixed-valence state, improving the material’s electrical conductivity and chemical stability. Of which, the nanosheet shape is maintained to provide fixed active sites for the next sulfuration step. The NiCoS_*x*_@Ni_3_S_2_/NF was formed via hydrothermal anion exchange by using thiourea as sulfur source as below. Under hydrothermal conditions at 120 °C, thiourea undergoes a decomposition reaction: CH_4_N_2_S + 2H_2_O → CO_2_ + 2NH_3_ + H_2_S. At the same time, NiCo_2_O_4_ reacts with sulfide ions (S^2^^−^) through a substitution reaction: NiCo_2_O_4_ + S^2^^−^ + H^+^ → NiCoS_*x*_ + H_2_O. During this process, Ni, Co, and S elements are co-introduced. The synergistic co-deposition effect suppresses atomic ordering, leading to the formation of an amorphous material with a unique structure. The co-deposition of multiple elements enhances the structural activity of the catalyst and helps improve its catalytic performance.

**Fig. 1:**
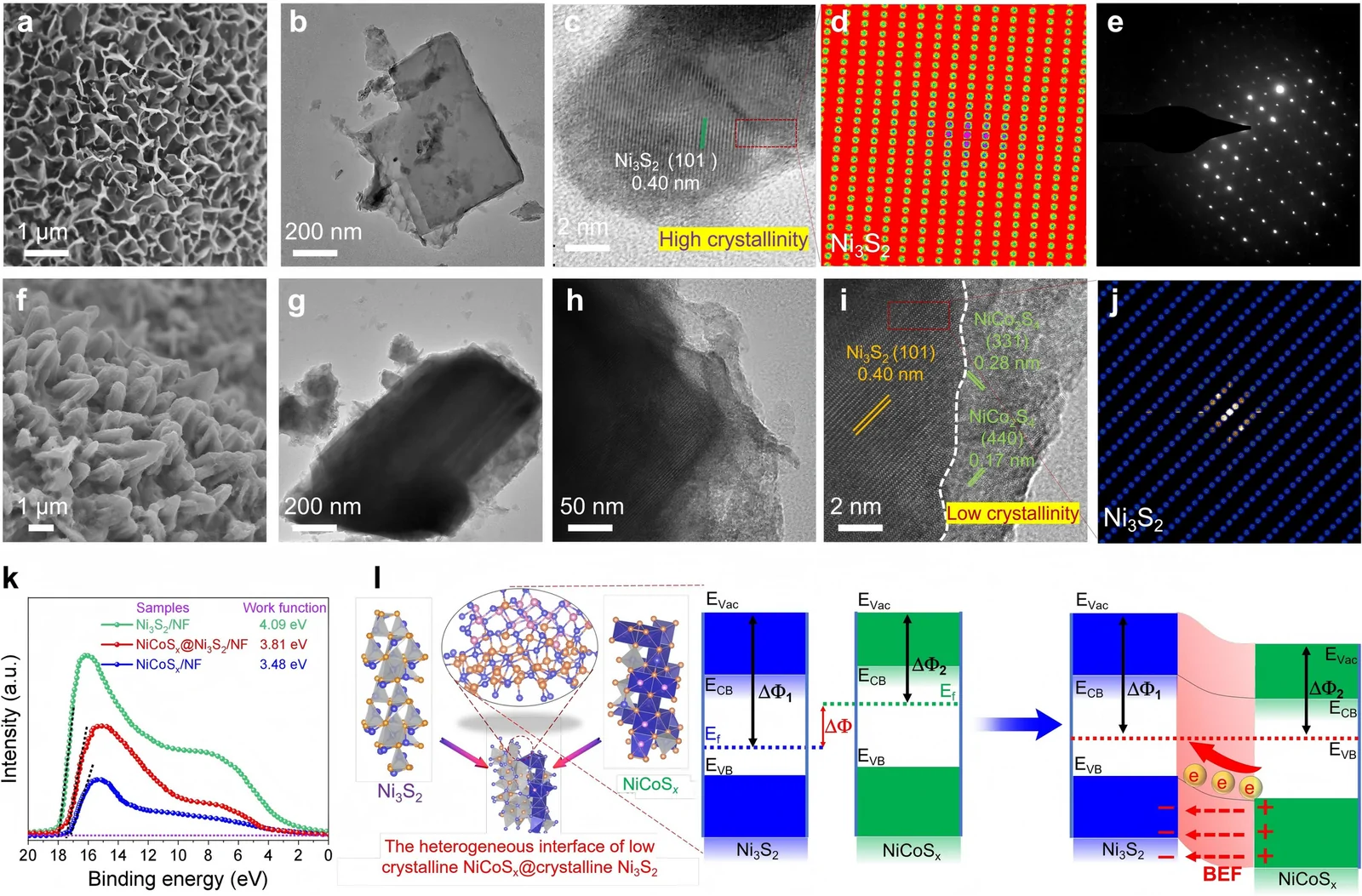
Structural characterization. NiCo_2_O_4_@NiO: **a** SEM image, **b** TEM image, **c** HRTEM image, **d** Corresponding high-resolution atomic image and **e** FFT pattern. NiCoS_*x*_@Ni_3_S_2_: **f** SEM image, **g**, **h** TEM images, **i** HRTEM image,** j** Corresponding high-resolution atomic image. **k** UPS spectra of Ni_3_S_2_/NF, NiCoS_*x*_@Ni_3_S_2_/NF, NiCoS_*x*_/NF. **l** Schematic diagram of the electron transfer process in the energy band of NiCoS_*x*_@Ni_3_S_2_

The elemental composition and crystal structure of the synthesized electrode materials were confirmed by X-ray diffraction (XRD) characterization. As illustrated in Fig. S2, all the samples exhibit the characteristic peaks at 44.5°, 51.9° and 76.5°, which match perfectly with the metal Ni. The XRD patterns of Ni_3_S_2_/NF show the characteristic peaks at 21.7°, 31.1°, 37.8°, 38.3°, 44.3°, 49.7°, 50.1°, and 54.6°, corresponding to the (101), (110), (003), (021), (202), (113), (211), and (104) crystal planes of Ni_3_S_2_ (JCPDS 48–0083), respectively. In addition to the characteristic peaks of Ni_3_S_2_, the XRD patterns of NiCoS_*x*_@Ni_3_S_2_/NF exhibit distinct diffraction peaks at 26.8°, 47.4°, 50.4°, 65.1°, and 78.2°, corresponding to the (220), (422), (511), (533), and (731) crystal planes of NiCo_2_S_4_ (JCPDS 20–0782), respectively [46]. Yet, the XRD patterns of NiCoS_*x*_ exhibit relatively weak diffraction peaks, suggesting its low crystallinity. After extensive etching by Co and S, only the diffraction peaks from the NF substrate remain observable, confirming the complete amorphization of NiCoS_*x*_ on the NF surface. Scanning electron microscopy (SEM) and transmission electron microscope (TEM) were performed to study the microstructure of the samples. As shown in Fig. S3, uniform Ni(OH)_2_/NF nanosheets are formed by etching growth on the NF substrate. Meanwhile, NiCo(OH)_2_/NF maintains the nanosheet morphology after further exchange of Ni^2+^ with Co^2+^. Furthermore, the NiCo_2_O_4_@NiO/NF nanosheets are formed through oxidative transformation of NiCo(OH)_2_/NF precursors in air, facilitated by Ni–Co bonding (Fig. 1a, b). The NiCo_2_O_4_@NiO/NF heterostructure displays well-defined lattice fringes with lattice spacing of 0.47 and 0.21 nm, corresponding to the (111) and (200) planes, respectively (Fig. 1c–e), revealing high crystallinity. Furthermore, the NiCoS_*x*_@Ni_3_S_2_/NF nanorods are formed by anion exchange method (Fig. 1f). The TEM results prove the presence of nanorods, along with thin layers at their edges (Fig. 1g, h). The HRTEM images reveal that the heterogeneous interface is directly observed at the phase boundary between the Ni_3_S_2_ and NiCoS_*x*_ (Fig. 1i). The observed 0.40 nm lattice fringes correspond to the (102) planes of crystalline Ni_3_S_2_, confirming the highly ordered structure in the nanorod core (Fig. 1j). However, the outer low-crystalline layer shows 0.28 and 0.17 nm fringes, matching NiCoS_*x*_ (211) and (100) planes, respectively. The above-mentioned structure displays extensive atomic disorder and defects, confirming low crystallinity. This morphology arises from the outward diffusion of Co^2^⁺ and the inward diffusion of S^2−^, coupled with the formation of nanoarray by S^2−^ etching. Both inner crystalline Ni_3_S_2_ layer (high conductivity) and outer low-crystalline NiCoS_*x*_ layer (rich defect) form a precisely tuned heterostructure. The scanning transmission electron microscopy-energy dispersive X-ray spectroscopy (TEM-EDS) analysis reveals the uniform distribution of Co, Ni, and S elements in NiCoS_*x*_@Ni_3_S_2_ nanorod (Fig. S4). Meanwhile, the molar fractions of Ni, S, and Co in NiCoS_*x*_@Ni_3_S_2_ are 61.82%, 36.77%, and 1.40%, respectively (Fig. S5, Supporting Information). For the Ni_3_S_2_/NF sample, the Ni/S atomic ratio is 60.6/39.4, which closely matches the theoretical stoichiometric ratio of 3/2 by using ICP-OES. For the NiCoS_*x*_/NF, the overall Ni/Co/S ratio is 58.2/6.7/35.5. For the NiCoS_*x*_@Ni_3_S_2_/NF sample, the Ni/Co/S ratio is 66.2/2.4/31.4, where the Ni signal primarily originates from the core of the substrate. The enrichment of cobalt at the interface is induced by the Ni–S–Co bridging bond. As a control sample, the Ni_3_S_2_ without Co ion exchange treatment maintains vertically aligned convex nanoarray (Fig. S6). The SEM analysis reveals that excessive sulfur etching generates rough nanoarrays of NiCoS_*x*_/NF (Fig. S7), while the TEM analysis indicates the formation of an amorphous structure (Fig. S8). Ultraviolet photoelectron spectroscopy (UPS) was employed to probe the interfacial interactions in low-crystalline NiCoS_*x*_@crystalline Ni_3_S_2_ heterogeneous interface. The ΔΦ is calculated as ΔΦ = h*ν*—E_cutoff_, where h*ν* represents the incident photon energy (21.22 eV) and the E_cutoff_ is derived from the cutoff energy from the UPS measurements. As shown in Fig. S9, the E_cutoff_ value for NiCoS_*x*_@Ni_3_S_2_/NF, Ni_3_S_2_/NF, and NiCoS_*x*_/NF is 17.41, 17.74, and 17.13 eV, respectively. The ΔΦ value for NiCoS_*x*_@Ni_3_S_2_ (ΔΦ** = **3.81 eV) is between that of Ni_3_S_2_/NF (ΔΦ = 4.09 eV) and NiCoS_*x*_/NF (ΔΦ = 3.48 eV) (Fig. 1k). These values produce an asymmetric concentration gradient of electrons, creating a BEF in the direction of the ΔΦ from low value (NiCoS_*x*_) to high value (Ni_3_S_2_). Furthermore, the band diagrams demonstrate the BEF-driven electron transfer from NiCoS_*x*_ to the NiCoS_*x*_@Ni_3_S_2_ interface, and ultimately to Ni_3_S_2_, realizing new equilibrium state (Fig. 1l). The BEF helps to reduce the energy barrier for electron escape from the surface of material, thereby promoting electron transfer. Therefore, synergistic chemical bond coupling and BEF-driven charge redistribution at the heterointerface enhance electron transfer and optimize intrinsic activity.

**Fig. 2:**
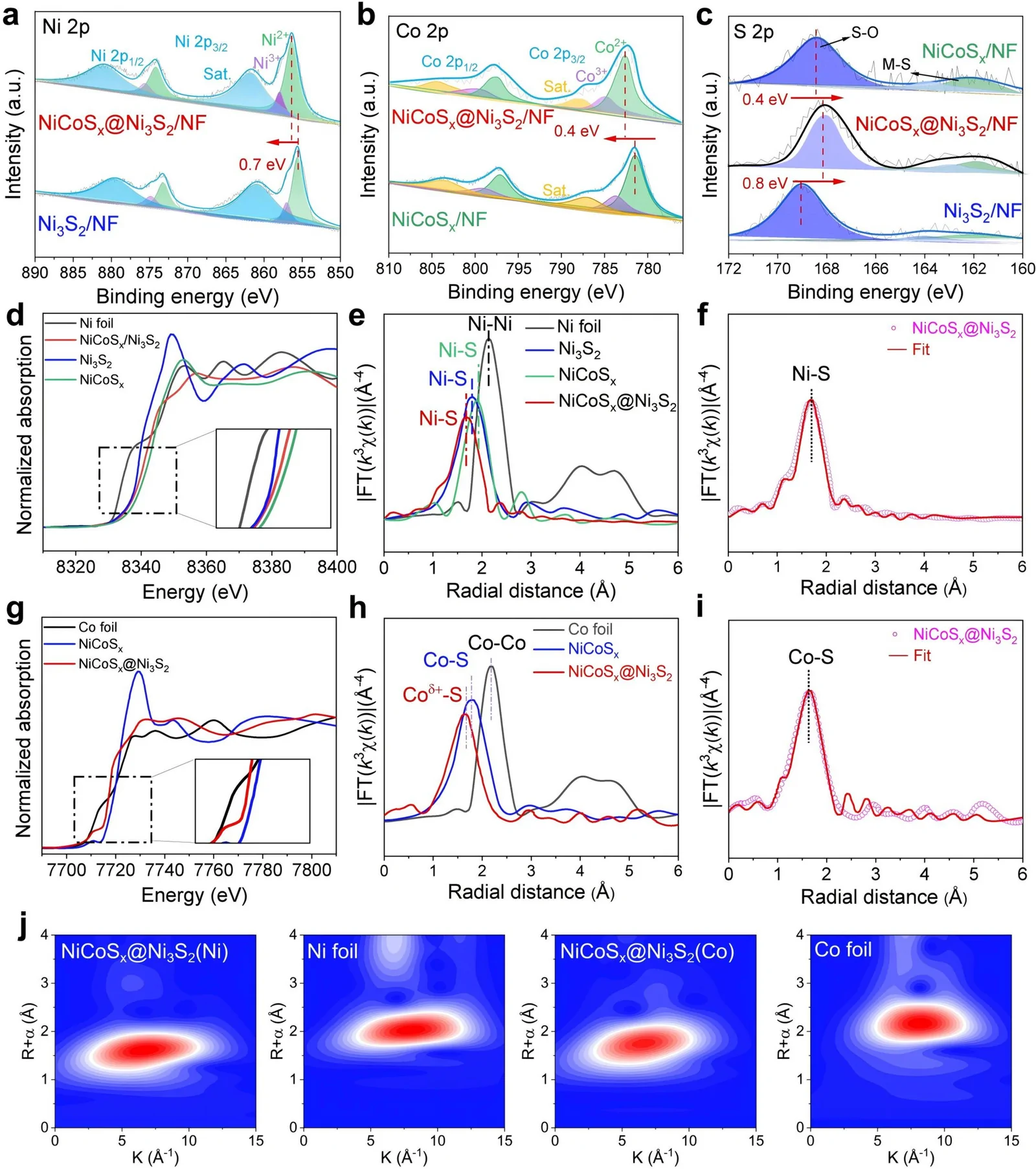
Electronic configuration and atomic structure. XPS spectra of Ni_3_S_2_, NiCoS_*x*_, and NiCoS_*x*_@Ni_3_S_2_: **a** Ni 2*p*, **b** Co 2*p*, **c** S 2*p*. **d**, **g** XANES spectra. **e**, **h** k^3^-weighted EXAFS spectra. **f**, **i** FT-EXAFS fitting results. **j** wavelet transform WT of Ni_3_S_2_, NiCoS_*x*_@Ni_3_S_2_, Ni foil and Co foil

The surface chemical compositions and valence distributions of Ni, Co and S in Ni_3_S_2_/NF, NiCoS_*x*_/NF and NiCoS_*x*_@Ni_3_S_2_/NF were analyzed by XPS technique. As shown in Fig. 2a, the binding energies of 855.5/861.0 and 873.1/879.2 eV correspond to the characteristic peaks of Ni^2^⁺ and Ni^3^⁺, which are assigned to Ni 2*p*_3/2_ and Ni 2*p*_1/2_, respectively. The binding energies of 780.3 and 783.2 eV correspond to the characteristic peaks of Co^2+^ and Co^3+^ (Fig. 2b). The main peak in the S 2*p* XPS spectra at 161.6 and 163.4 eV is assigned to the S–O bond and metal-S bond, respectively (Fig. 2c). Notably, XPS analysis reveals that a positive shift of 0.7 eV in the binding energy of Ni 2*p* indicates the oxidation Ni^3+^ from Ni^2+^, and a positive shift of 0.4 eV in the Co 2*p* binding energy confirms the formation of Co^3+^. Concurrently, the S 2*p* binding energy exhibits negative shifts of 0.4 and 0.8 eV, respectively, collectively attributing to interfacial electron transfer from Ni and Co to S. In the heterostructure, Ni 2*p*_3/2_ and Co 2*p*_3/2_ peaks shift positively relative to the pure phases, indicating partial electron depletion on the shell side, while the negative shift of the S 2*p* peaks suggests stronger metal-S interactions. The optimized charge redistribution at the heterointerface enhances the adsorption energy of OER intermediates (*OH → *O) and improves H_2_O dissociation kinetics. This synergistic effect originates from the heterointerface between the low-crystalline NiCoS_*x*_ layer and the crystalline Ni_3_S_2_ phase, which facilitates rapid electron transfer and stabilizes the active sites, ultimately achieving high catalytic activity.

X-ray absorption spectroscopy techniques, including XANES and extended X-ray absorption fine structure (EXAFS) spectra were explored to probe the electronic properties, valence states, and local coordination environments of Ni_3_S_2_, NiCoS_*x*_, and NiCoS_*x*_@Ni_3_S_2_. Ni K-edge XANES shows the pre-edge positive peak shifts of NiCoS_*x*_@Ni_3_S_2_ versus Ni_3_S_2_, indicating that Co promotes Ni to higher oxidation states (Fig. 2d). Co K-edge XANES reveals a slight pre-edge peak downshift of NiCoS_*x*_@Ni_3_S_2_ versus NiCoS_*x*_ (Fig. 2g). The results indicate an electron transfer pathway from NiCoS_*x*_ to NiCoS_*x*_@Ni_3_S_2_, subsequently to Ni_3_S_2_, in agreement with the XPS and UPS results. R-space resolution analysis reveals two prominent coordination peaks at the Ni and Co K-edges of NiCoS_*x*_@Ni_3_S_2_ at 1.76 and 1.82 Å, corresponding to Ni-S and Co-S bonds, respectively. Moreover, NiCoS_*x*_@Ni_3_S_2_ exhibits shorter Ni–S (1.87 Å) and Co–S (1.89 Å) bonds than pure phases, indicating stronger interfacial bonding (Fig. 2e, h). The EXAFS spectra are fitted to investigate the coordination environment and coordination number (CN) of NiCoS_*x*_@Ni_3_S_2_ (Fig. 2f, i and Table S1). The EXAFS analysis of NiCoS_*x*_@Ni_3_S_2_ confirms the Ni–S bond (2.05 Å, CN = 2.7) and Co-S (2.11 Å, CN = 2.11) bond. The shortening of the Ni–S and Co–S bond length is ascribed to the formation of Ni–S–Co interfacial bridge in NiCoS_*x*_@Ni_3_S_2_. Wavelet transform analysis of Ni K-edge EXAFS oscillations is probed to investigate the atomic dispersion properties (Fig. 2j). The maximum intensity of the Ni foil at 2.3 Å, and Co foil at 2.1 Å is ascribed to the electronic contributions of both Ni–Ni and Co–Co bond, respectively. The maximum intensity of NiCoS_*x*_@Ni_3_S_2_ at 1.5 and 1.8 Å is plausibly interpreted as the contribution of the Ni–S bond and Co–S bond. These values are smaller than the maximum intensity of the pure Ni and Co foil. Overall, these findings demonstrate that the low-crystalline NiCoS_*x*_ shell and crystalline Ni_3_S_2_ core undergo significant interfacial charge redistribution, effectively stabilizing lattice sulfur. The resulting Ni–S–Co interfacial bridges create new electron-transfer pathways, which not only suppress oxidative degradation during OER but also enhance both the activity and durability of NiCoS_*x*_@Ni_3_S_2_ compared to single-phase catalysts.

Compared with conventional sulfide catalysts (e.g., NiCo_2_S_4_, Co_9_S_8_, Ni_3_S_2_), the low-crystalline NiCoS_*x*_ shell is the critical component for enhancing the overall water splitting performance of NiCoS_*x*_@Ni_3_S_2_, with core contributions [47–51]: 1) Atomic disorder and abundant defects boost the exposure of unsaturated coordination active sites, as reflected by high electrochemical active surface area (ECSA) of the catalyst; 2) The work function difference between low-crystalline NiCoS_*x*_ and crystalline Ni_3_S_2_ induces a built-in electric field, which, coupled with Ni–S–Co interfacial bridges, optimizes the d-band center of Ni/Co active sites and facilitates directional charge transfer, laying the groundwork for accelerated reaction kinetics. These structural and electronic merits of the low-crystalline NiCoS_*x*_ shell, in synergy with the high-conductivity crystalline Ni_3_S_2_ core, are expected to responsible for the superior HER and OER performance of NiCoS_*x*_@Ni_3_S_2_ as elaborated in the following sections.

### Hydrogen Evolution Catalysis

**Fig. 3:**
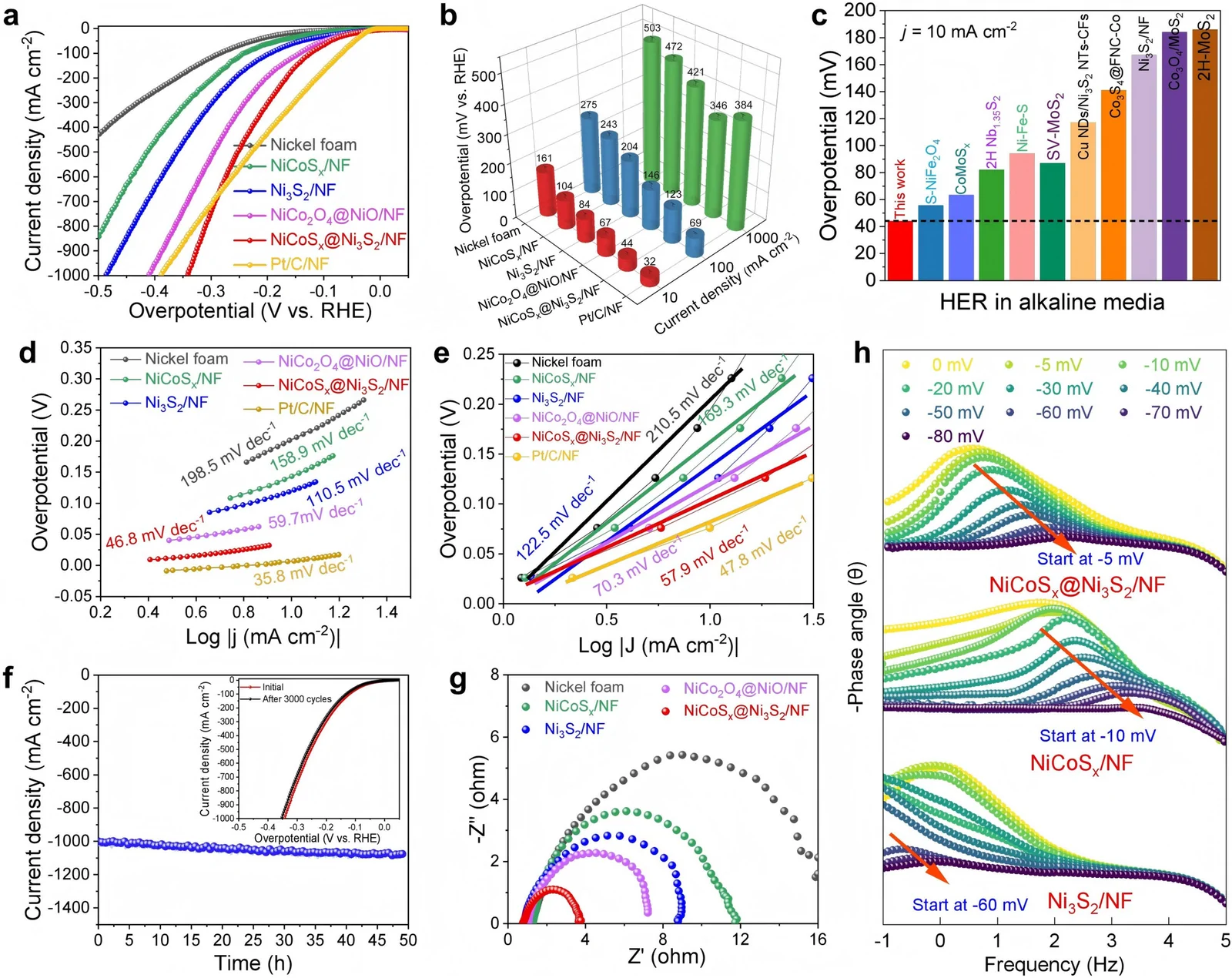
HER catalytic activity of Ni_3_S_2_/NF, NiCoS_*x*_/NF, NiCo_2_O_4_@NiO/NF, NiCoS_*x*_@Ni_3_S_2_/NF and Pt/C/NF in 1.0 M KOH solution: **a** LSV polarization curves, **b** Comparison of overpotentials at 10, 100, and 1000 mA cm^−2^. **c** Comparison of HER performance among the reported electrocatalysts. **d** Tafel plots. **e** Steady-state polarization Tafel curves. **f** Current density time-dependent test of NiCoS_*x*_@Ni_3_S_2_/NF. **g** Nyquist plots. **h** Phase angle plots of EIS data at various voltages for Ni_3_S_2_/NF, NiCoS_*x*_/NF and NiCoS_*x*_@Ni_3_S_2_/NF

The HER performance of NiCoS_*x*_@Ni_3_S_2_/NF was systematically evaluated against control samples (NiCoS_*x*_/NF, Ni_3_S_2_/NF, NiCo_2_O_4_@NiO/NF) via a three-electrode system. As shown in Fig. 3a, b, NiCoS_*x*_@Ni_3_S_2_/NF consistently exhibits the lowest overpotentials across all tested conditions, requiring 44, 123, and 346 mV at 10, 100, and 1000 mA cm^−2^, respectively. This demonstrates its excellent catalytic activity and remarkable stability under high current densities. In contrast, Pt/C/NF shows strong performance at low current densities (32 mV at 10 mA cm^−2^), but its overpotential increases sharply to 384 mV at 1000 mA cm^−2^, indicating poor stability at high currents. Notably, the η_10_ value of NiCoS_*x*_@Ni_3_S_2_/NF outperforms that of most other related transition metal sulfide-based composites (Fig. 3c, Table S2). In contrast, commercial Pt/C possesses the best HER activity, but Pt/C is disadvantageous at high current densities (> 712 mA cm^−2^). Tafel plots analysis from LSV data reveals the HER kinetics and intrinsic catalytic activity. The NiCoS_*x*_@Ni_3_S_2_/NF shows superior HER kinetics (Tafel slope = 46.8 mV dec^−1^) versus controls samples: NF (198.5 mV dec^−1^), NiCoS_*x*_/NF (158.9 mV dec^−1^), Ni_3_S_2_/NF (110.5 mV dec^−1^), and NiCo_2_O_4_@NiO/NF (59.7 mV dec^−1^) (Fig. 3d).

To reflect HER kinetics more accurately, the Tafel slope is calculated by steady-state polarization at constant potentials. Specifically, chronoamperometry (CA) was performed for 150 s of steady-state current density in the voltage of − 0.45 ~ 0 V versus RHE. The Tafel slope of NiCoS_*x*_@Ni_3_S_2_/NF for the steady-state polarization method is 57.9 mV dec^−1^, which is significantly better than the comparison samples of NF (210.5 mV dec^−1^), NiCoS_*x*_/NF (169.3 mV dec^−1^), Ni_3_S_2_/NF (122.5 mV dec^−1^), and NiCo_2_O_4_@NiO/NF (70.3 mV dec^−1^) (Fig. 3e). The steady-state and instantaneous Tafel slopes show similar trends, confirming that NiCoS_*x*_@Ni_3_S_2_/NF accelerates the rate-determining steps of Volmer-Heyrovsky in HER (Figs. S10–S13). In addition, the NiCoS_*x*_@Ni_3_S_2_/NF electrode exhibits excellent durability, maintaining a stable current density of 1000 mA cm^−2^ for 48 h with negligible decay. Moreover, the polarization curves before and after 3000 cycles nearly overlap, confirming its robust catalytic activity and structural stability for practical HER applications (Fig. 3f). The TOF value of NiCoS_*x*_@Ni_3_S_2_ (265 s^−1^) is significantly bigger than that of NiCo_2_O_4_@NiO/NF (132 s^−1^), Ni_3_S_2_/NF (41 s^−1^) and NiCoS_*x*_/NF (19 s^−1^) at 50 mV, reflecting superior intrinsic HER activity (Figs. S14 , S15). NiCoS_*x*_@Ni_3_S_2_/NF shows the highest ECSA (25.1 mF cm^−2^) compared with NiCo_2_O_4_@NiO/NF (12.9 mF cm^−2^), Ni_3_S_2_/NF (10.1 mF cm^−2^) and NiCoS_*x*_/NF (5.2 mF cm^−2^) (Figs. S16, S17), demonstrating the sulfur defects and low crystallinity boost the exposure of the active site. Furthermore, electrochemical impedance spectroscopy (EIS) measurements were conducted to probe charge transfer kinetics. The NiCoS_*x*_@Ni_3_S_2_/NF demonstrates the lowest charge transfer resistance (R_ct_ = 7.2 Ω), smaller than other control samples: NF (> 16 Ω), Ni_3_S_2_/NF (9.1 Ω), NiCoS_*x*_/NF (11.6 Ω) (Fig. 3g). Operando EIS is used to probe the charge transfer mechanism of HER in Ni_3_S_2_/NF, NiCo_2_O_4_@NiO/NF and NiCoS_*x*_@Ni_3_S_2_/NF across 0 to − 80 mV, with Bode plot analysis (Fig. 3h). The high-frequency region of Bode phase plots reflects the electron transfer between catalyst bulk and active sites, corresponding to the Volmer step. Conversely, the mid- and low-frequency regions are indicative of the Heyrovsky step. The HER proceeds via the Volmer-Heyrovsky pathway, where chemisorbed H atoms react with H_2_O to generate H_2_. The frequency peak gradually decreases and shifts to higher frequencies as the applied potential decreases. This indicates the reduced charge-transfer resistance at the catalyst-electrolyte interface with enhanced reaction kinetics. Notably, NiCoS_*x*_@Ni_3_S_2_/NF exhibits both reduced phase angles and accelerated high-frequency peak shifts compared to Ni_3_S_2_/NF and NiCo_2_O_4_@NiO/NF at the equal potentials. Thus, the NiCoS_*x*_@Ni_3_S_2_/NF promotes the adsorption of water and hydrogen, resulting in accelerated HER kinetics.

### Oxygen Evolution Catalysis

**Fig. 4:**
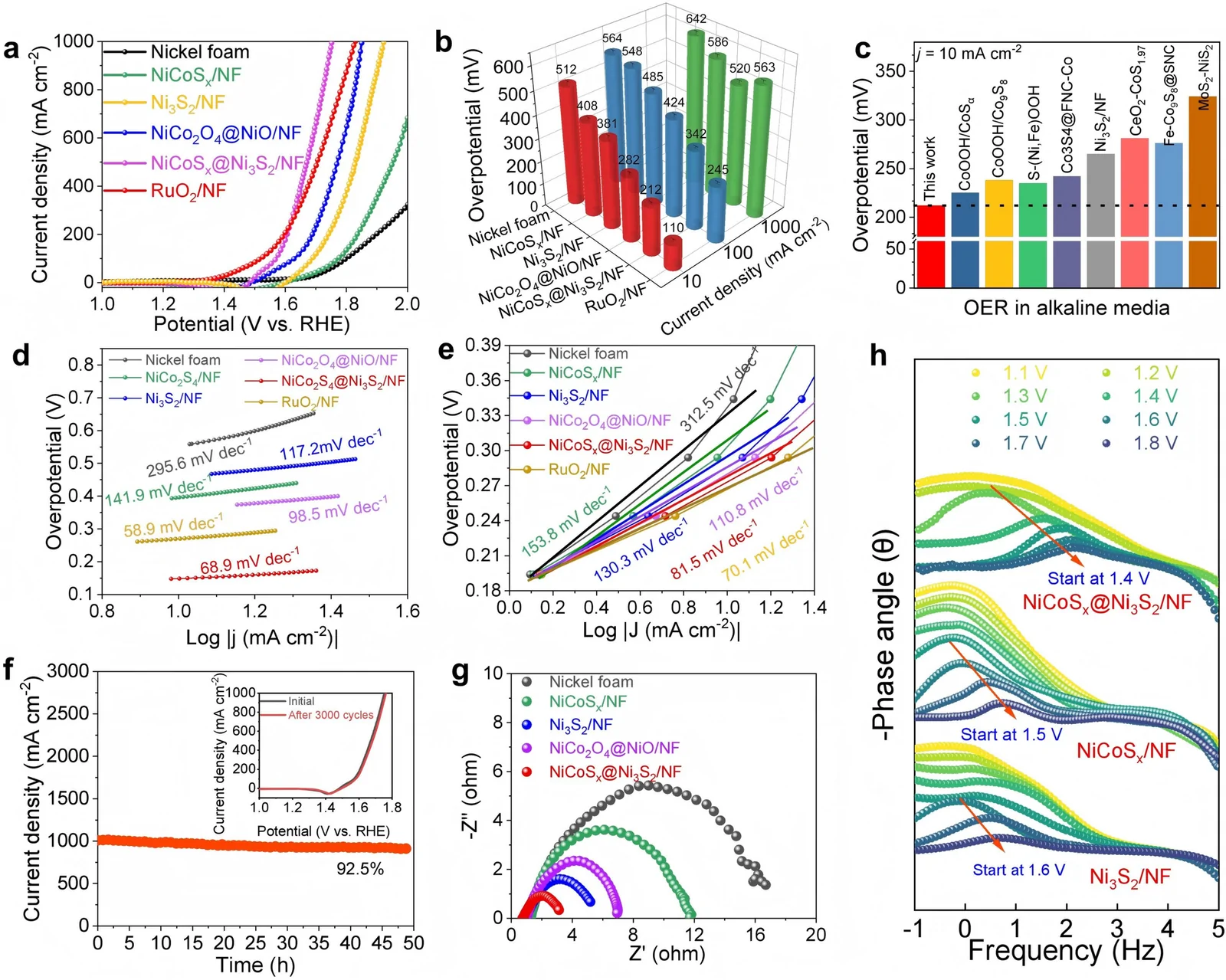
OER catalytic activity of Ni_3_S_2_/NF, NiCoS_*x*_/NF, NiCo_2_O_4_@NiO/NF, NiCoS_*x*_@Ni_3_S_2_/NF and RuO_2_/NF in 1.0 M KOH solution: **a** LSV polarization curves, **b** Comparison of overpotentials at 10, 100, 1000 mA cm^−2^. **c** Comparison of OER performance among the reported electrocatalysts. **d** Tafel plots. **e** Steady-state polarization Tafel curves. **f** Current density time-dependent test of NiCoS_*x*_@Ni_3_S_2_/NF. **g** Nyquist plots. **h** Phase angle plots of EIS data at various voltages for Ni_3_S_2_/NF, NiCoS_*x*_/NF and NiCoS_*x*_@Ni_3_S_2_/NF

Given its exceptional HER performance, NiCoS_*x*_@Ni_3_S_2_/NF demonstrates equally outstanding OER activity. As shown in Fig. 4a, the OER polarization curves reveal that NiCoS_*x*_@Ni_3_S_2_/NF delivers the highest current densities at lower overpotentials compared with other electrodes, including bare NF, Ni_3_S_2_/NF, NiCoS_*x*_@Ni_3_S_2_/NF, NiCo_2_O_4_@NiO/NF and RuO_2_/NF. This superior performance is further quantified in Fig. 4b, where NiCoS*ₓ*@Ni_3_S_2_/NF exhibits the lowest overpotentials of 212, 342, and 520 mV at 10, 100, and 1000 mA cm^−2^, respectively. In comparison, RuO_2_/NF, Ni_3_S_2_/NF and NiCo_2_O_4_@NiO/NF exhibit significantly higher overpotentials. The remarkable OER activity of NiCoS_*x*_@Ni_3_S_2_/NF is attributed to the synergistic effect of the heterostructured interface, which facilitates rapid charge transfer and optimizes the exposure of active sites, thereby enabling both low onset potential and robust catalytic efficiency under high current densities. Notably, the η_10_ value of NiCoS_*x*_@Ni_3_S_2_/NF surpasses that of reported TMS-based composites (Fig. 4c, Table S3). The NiCoS_*x*_@Ni_3_S_2_/NF demonstrates superior OER performance, exhibiting a significantly lower Tafel slope (58.9 mV dec^−1^) compared to control samples (NF: 295.6 mV dec^−1^, NiCoS_*x*_/NF: 141.9 mV dec^−1^, Ni_3_S_2_/NF: 117.2 mV dec^−1^, and NiCoS_*x*_@Ni_3_S_2_/NF: 98.5 mV dec^−1^) (Fig. 4d).

For consistent OER kinetic analysis, Tafel slopes are derived from steady-state polarization at constant potentials, following the same methodology employed for HER analysis (Figs. 4e, S18 and S19). The transient Tafel slopes of these materials are consistent with the Tafel slopes of the steady state polarization method (Fig. S20). In electrocatalytic reaction kinetics, the difference between steady-state and transient Tafel slopes primarily arises from the dynamic adsorption equilibrium of intermediates on the electrode surface (Figs. S13 and S20). The steady-state Tafel slope of NiCoS_*x*_@Ni_3_S_2_/NF is higher than the transient one, which is explained by the following factors: (1) During steady-state polarization, the continuous application of potential leads to the gradual accumulation of H*/*OH intermediates on the active sites, creating an additional adsorption energy barrier; (2) The low crystallinity and high defect density of the NiCoS*ₓ* layer provide more intermediate adsorption sites, further hindering charge transfer; (3) The heterojunction interface undergoes dynamic reconstruction under prolonged polarization, and some active sites become passivated. In contrast, transient measurements capture the intrinsic kinetics of the initial electron transfer step, where intermediate accumulation is not yet significant, thus reflecting the theoretical kinetics more accurately. The TOF value of NiCoS_*x*_@Ni_3_S_2_/NF (126 s^−1^) at the overpotential of 400 mV is significantly higher than that of NiCoS_*x*_/NF (17 mV dec^−1^), Ni_3_S_2_/NF (39 mV dec^−1^) and NiCo_2_O_4_@NiO/NF (75 mV dec^−1^), which is consistent with the performance of OER (Figs. S21, S22). In addition, the NiCoS*ₓ*@Ni_3_S_2_/NF electrode maintains 92.5% of its initial current density after 48 h at 1000 mA cm^−^^2^, and the polarization curves before and after 3000 cycles nearly overlap, confirming its excellent durability and structural stability for OER (Fig. 4f). As illustrated in Figs. S23  and S24, the NiCoS_*x*_@Ni_3_S_2_/NF exhibits the highest ECSA of 21.4 mF cm^−2^, surpassing that of NiCo_2_O_4_@NiO/NF (16.0 mF cm^−2^), Ni_3_S_2_/NF (9.8 mF cm^−2^), and NiCoS_*x*_/NF (2.0 mF cm^−2^). In addition, the increased ECSA value indicates that the low-crystalline structure effectively increases the accessibility of superior active sites. The NiCoS_*x*_@Ni_3_S_2_/NF demonstrates the lowest charge transfer resistance (R_ct_ = 3.1 Ω), surpassing contrast samples: NiCo_2_O_4_@NiO/NF (7.1 Ω), Ni_3_S_2_/NF (5.2 Ω), NiCoS_*x*_/NF (12 Ω), and NF (17 Ω) (Fig. 4g). Moreover, operando EIS measurements on Ni_3_S_2_/NF, NiCoS_*x*_/NF and NiCoS_*x*_@Ni_3_S_2_/NF provide deeper insight into the kinetics of the water oxidation reaction (Fig. 4h). Notably, the high-frequency region (10^2^–10^4^ Hz) reflects inner-layer electron transfer from bulk to surface active sites, while the low-frequency range (10^−2^ ~ 10^2^ Hz) reveals interfacial charge transfer at the catalyst/electrolyte interface. The NiCoS_*x*_@Ni_3_S_2_/NF shows OER phase angle reduction at 1.4 V through low-frequency phase angle, outperforming NiCo_2_O_4_@NiO/NF (1.5 V) and Ni_3_S_2_/NF (1.6 V). The NiCoS_*x*_@Ni_3_S_2_/NF exhibits reduced peak intensities across both the frequency domains, with accelerated attenuation as the applied potential increases. These results suggest that the low-crystalline NiCoS_*x*_-tuned crystalline Ni_3_S_2_ heterogeneous interface enhances both capacitive behavior and -OH adsorption, accelerating OER kinetics.

### Cation and Anion Exchange Regulation Mechanism

In both cation exchange and anion exchange sulfidation processes, etching temperature and reaction time collaboratively regulate the catalyst's morphology, composition, phase structure, and defect characteristics, establishing a precise “structure-performance” relationship that determines its bifunctional HER/OER electrocatalytic activity.

Temperature regulates the thermodynamic and kinetic balance of ion exchange, thereby precisely controlling Co^2+^ doping efficiency, morphology, and crystallinity (Fig. S25). At 40 °C, ion exchange is slow, leading to incomplete Co^2+^ doping, disordered morphology, and limited active site exposure. At 80 °C, the morphology becomes more regular, and site exposure improves. At 120 °C, the system reaches thermodynamic equilibrium, with arrays becoming more regular and dispersed, and the active site exposure reaches its peak. ICP-MS results show that the Co/Ni ratio increases from 0.012 at 40 °C to 0.036 at 120 °C due to accelerated Ni dissolution and Co enrichment, which matches the moderately low crystallinity structure (Fig. S26). As the Co^2+^ exchange temperature increases, the EPR signal intensity gradually strengthens, indicating that higher temperatures promote the incorporation of Co^2+^ into the carrier lattice, thereby inducing a higher concentration of lattice defects (Fig. S27). The LSV curves for HER and OER at different Co^2+^ exchange temperatures indicate that the optimal catalytic activity occurs at 120 °C, while 160 °C results in decreased performance (Fig. S28).

Time governs the morphological evolution and Co^2+^ doping depth, impacting active site exposure and mass transfer efficiency (Fig. S29). At 0.5 h, the catalyst is a dense substrate with limited active sites and low mass transfer efficiency. After 1 h, discrete nanosheets form, but the structure lacks regularity. After 3 h, the nanosheets crosslink into an open layered structure, achieving the optimal synergy between active site exposure and mass transfer efficiency. At 6 h, excessive growth and agglomeration bury the active sites, leading to performance degradation. ICP-MS and electrochemical tests confirm that at 3 h, the Co/Ni ratio is 0.036, with the optimal synergy between doping and morphology, resulting in the best HER/OER activity (Figs. S30 and S31).

Sulfidation time regulates the morphology, phase structure, and defect concentration of the catalyst. From 0.5 to 2 h, the surface forms nanostructures like nanospikes, particles, and discrete nanosheets, with poor site exposure and mass transfer (Fig. S32). After 3 h, a flower-like array (the optimal morphology) forms, significantly improving site exposure and mass transfer efficiency. At 6 h, excessive growth and agglomeration bury the active sites. The XRD result shows that after 3 h, a highly crystalline NiCoS*ₓ*@Ni_3_S_2_ heterojunction is formed, while after 6 h, it transitions to a pure-phase NiCoS*ₓ*, losing the heterojunction advantage (Fig. S33). Electron paramagnetic resonance (EPR) spectroscopy was employed to detect defect-induced unpaired electrons, confirming the low-crystalline nature of NiCoS_*x*_@Ni_3_S_2_. Intensive sulfide etching amplifies these defects, with the defect concentration initially increasing and peaking at 3 h, providing the most active sites. After 6 h, excessive defects reduce stability (Fig. S34). Electrochemical tests confirm that 3 h is the optimal sulfidation time (Fig. S35).

Temperature significantly affects the morphology, phase, and defects of the catalyst during sulfidation (Fig. S36). At 80 °C, the catalyst forms a loose fibrous structure, with poor mass transfer and stability. At 120 °C, the catalyst forms dense aggregates with low porosity and insufficient active sites. At 160 °C, the catalyst forms a high surface area flower-like array (the optimal morphology) (Fig. S37). At 200 °C, severe agglomeration leads to the loss of structural advantages. The XRD result shows that at 160 °C, high crystallinity target sulfide phases are formed, enhancing electron transport. At 200 °C, the target phase proportion decreases. The EPR results show that at 160 °C, defect concentration and active site exposure are optimally balanced. Electrochemical tests confirm that the HER/OER activity is the best at 160 °C (Fig. S38).

Thus, cation exchange (120 °C, 3 h) and anion exchange sulfidation (3 h, 160 °C) collaboratively regulate the morphology, phase and defects of the catalyst. This precise optimization achieves the optimal balance between active site exposure, structural stability, and electron transport efficiency, maximizing its bifunctional electrocatalytic performance.

### Surface Reconstruction Dynamics and Corrosion Resistance in OER

**Fig. 5:**
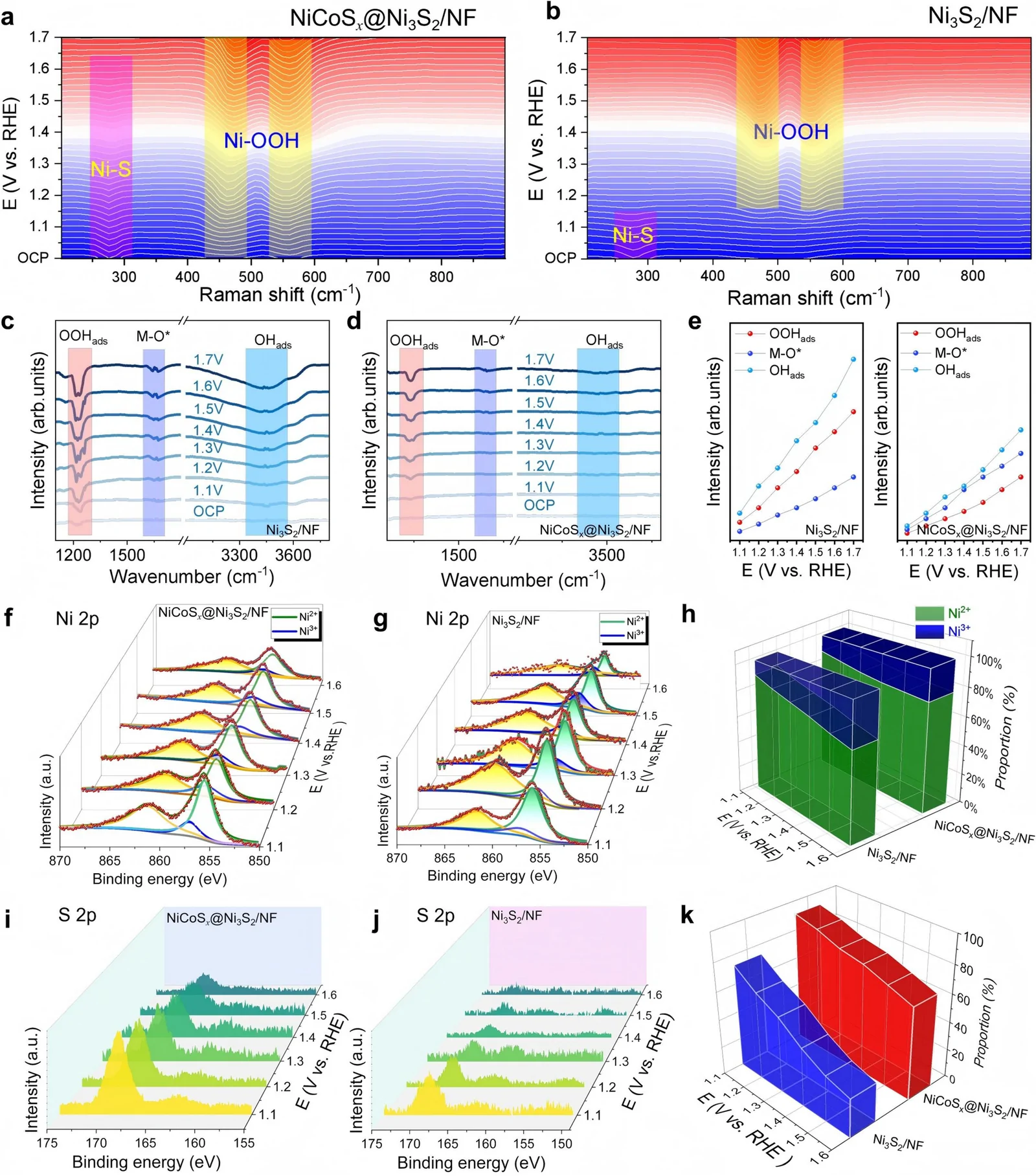
Potential-dependent in situ Raman spectra of: **a** NiCoS_*x*_@Ni_3_S_2_/NF, **b** Ni_3_S_2_/NF. In situ FTIR spectra of **c** Ni_3_S_2_/NF and **d** NiCoS*ₓ*@Ni_3_S_2_/NF catalyst under different applied potentials. **e** Variation in Raman signal intensity of OOH_ads_, M–O, and OH_ads_ species on NiCoS_*x*_@Ni_3_S_2_/NF and Ni_3_S_2_/NF catalysts with applied potential. XPS spectra of Ni 2*p*: **f** NiCoS_*x*_@Ni_3_S_2_/NF, **g** Ni_3_S_2_/NF under different potentials. **h** XPS fitting analysis results of Ni^2+^ and Ni^3+^ ratios for Ni_3_S_2_/NF and NiCoS_*x*_@Ni_3_S_2_/NF under different potentials. XPS spectra of S 2*p*: **i** NiCoS_*x*_@Ni_3_S_2_/NF, **j** Ni_3_S_2_/NF under different potentials. **k** Characteristic peaks of sulfur as percentage of S 2*p* for Ni_3_S_2_/NF and NiCoS_*x*_@Ni_3_S_2_/NF under different potentials

Oxygen evolution catalysis operando raman spectroscopy was employed to dynamically monitor the surface reconstruction and oxidative corrosion of sulfur species, providing insights into the evolution of electrocatalytic active centers. As shown in Fig. 5a, b, Raman spectroscopy analysis identifies characteristic peaks at 271 ~ 285 cm^−1^ (attributed to the Ni-S phase) and at 462 ~ 477/547 ~ 558 cm^−1^ (attributed to Ni–O phase in Ni–OOH). The Ni–S phase peak of NiCoS_*x*_@Ni_3_S_2_/NF and Ni_3_S_2_/NF completely disappears at 1.52 and 1.15 V, respectively. This confirms that low-crystalline NiCoS_*x*_ stabilizes metal-sulfur (M-S) bond in crystalline Ni_3_S_2_, effectively enhancing catalytic stability via suppressing sulfide oxidation and corrosion to some degree. Simultaneously, the NiCoS_*x*_@Ni_3_S_2_ heterointerface promotes spontaneous Ni–OOH formation at lower open circuit potential (OCP) than Ni_3_S_2_/NF (1.20 V) through interfacial electron redistribution, reducing the activation potential. The oxidation mechanism achieves breakthrough performance through synergistic effects as below: (1) The heterogeneous interface of NiCoS_*x*_/Ni_3_S_2_ promotes low-potential Ni–OOH generation, boosting catalytic kinetics via synergistic surface amorphization and active-phase reconstruction. (2) Co-induced amorphous NiCoS_*x*_ surface enables dynamic phase reconstruction, achieving high yet balanced activity and stability for OWS via an interface engineering strategy. What’s more, the surface intermediate evolution dynamics of NiCoS_*x*_@Ni_3_S_2_/NF and Ni_3_S_2_/NF across a 1.0 ~ 1.7 V potential window were probed via in-situ FTIR spectroscopy. In-situ FTIR spectra revealed that both catalysts exhibited distinct characteristic signals attributable to OOH_ads_, M–O* and OH_ads_ species, with signal intensities escalating monotonically with increasing applied potential. Notably, Ni_3_S_2_ displays substantially intensified M–O* and OH_ads_ signals, a hallmark of adsorbate evolution mechanism (AEM) relying on surface intermediate accumulation (Fig. 5c). By contrast, weaker intermediate signals of NiCoS_*x*_@Ni_3_S_2_/NF imply predominant lattice oxygen evolution mechanism (LOM) contribution (Fig. 5d). Collectively, these findings confirm that the amorphous NiCoS_*x*_ outer layer on NiCoS_*x*_@Ni_3_S_2_/NF induces sulfur-oxygen substitution to form an oxygen-rich surface, which elevates LOM contribution, promotes reconstruction into M-OOH, and boosts OER performance (Fig. 5e). In situ XPS technique at various applied potentials was employed to monitor potential-dependent evolution of electronic structures during OER. As shown in the Fig. 5f, g, the concentration of Ni^3+^ species on Ni_3_S_2_ progressively accumulates as the applied potential increases, accompanied by a positive shift in the Ni 2*p* binding energy. This result suggests electron transfer along the Ni-S coordination direction, leading to the oxidation of Ni species. Compared to Ni_3_S_2_, the NiCoS_*x*_@Ni_3_S_2_/NF shows stable Ni^3+^ valence state in Ni 2*p* XPS spectra, confirming the stability of valence during electrocatalysis (Fig. 5h).

During OER polarization, NiCoS_*x*_@Ni_3_S_2_/NF maintains persistent Co-S/Ni-S bonds, but Ni_3_S_2_/NF shows progressive bond degradation, demonstrating the enhanced structural stability of the former. As the OER potential increases, the Co–S/Ni–S bonding signals in Ni_3_S_2_/NF exhibit significant attenuation, while those remain stable in NiCoS_*x*_@Ni_3_S_2_/NF. Therefore, the incorporation of Co and S into NiCoS_*x*_@Ni_3_S_2_/NF endows the catalyst with dual functionality: it facilitates surface reconstruction and stabilizes the M–S bonds, thereby achieving an optimal balance between catalytic activity and structural durability. In situ S 2*s* XPS spectra show that, at potentials ranging from 1.1 to 1.6 V (vs. RHE), the shifts in binding energy and intensity of sulfur species for NiCoS_*x*_@Ni_3_S_2_/NF are significantly more pronounced than those for Ni_3_S_2_/NF (Fig. 5i, j). Quantitative results further demonstrate that NiCoS_*x*_@Ni_3_S_2_/NF maintains a higher proportion of active sulfur species across all applied potentials (Fig. 5k), confirming that NiCoS_*x*_ modification enriches active sulfur sites and thereby enhance electrocatalytic performance and stability.

These findings confirm that, while complete suppression of sulfide leaching is unattainable under strongly oxidative OER conditions, the NiCoS_*x*_ shell substantially mitigates sulfur depletion across a broad operational range. This enhancement is ascribed to the synergistic effects of robust Ni–S–Co interfacial bonding that strengthens lattice sulfur retention, the formation of a reconstructed NiCoOOH-rich surface layer serving as a physical and chemical barrier and a ΔΦ-induced BEF that optimizes interfacial charge distribution and attenuates oxidative degradation at sulfide sites.

### Evaluation of Overall Water Splitting and Device Applications

**Fig. 6:**
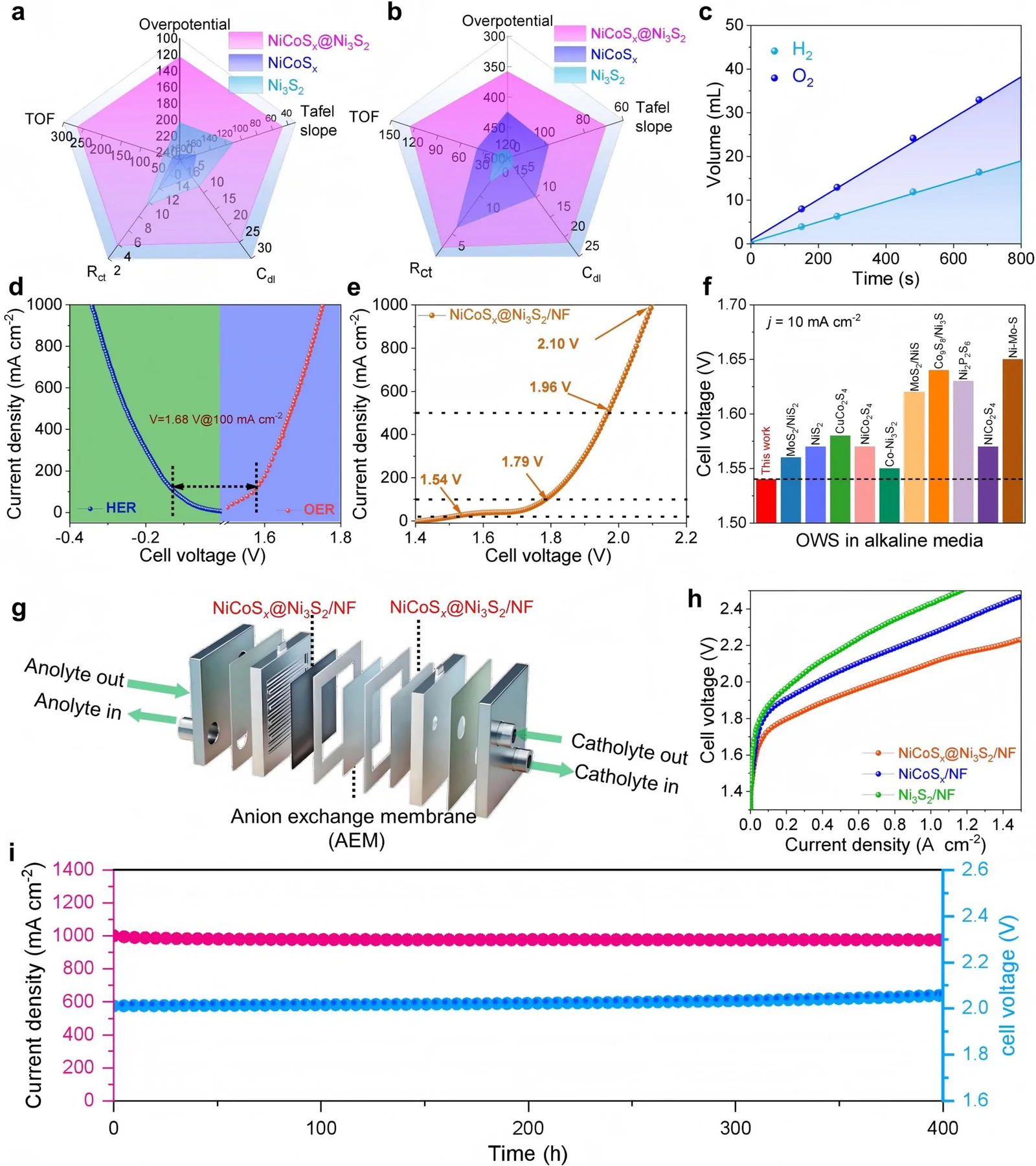
**a** HER and **b** OER performance of Ni_3_S_2_/NF, NiCoS_*x*_*/*NF and NiCoS_*x*_@Ni_3_S_2_/NF. **c** Volume of produced H_2_ and O_2_ as a function of time in a NiCoS_*x*_@Ni_3_S_2_/NF electrolyzer. **d** HER and OER LSV polarization curves of NiCoS_*x*_@Ni_3_S_2_/NF. **e** LSV polarization curves of NiCoS_*x*_@Ni_3_S_2_/NF||NiCoS_*x*_@Ni_3_S_2_/NF for OWS. **f** Comparison of OWS performance among the reported electrocatalysts. **g** Schematic diagram of the electrolyzer. **h** Polarization curves of the electrolyzer. **i** Current density and cell voltage curves of the electrolyzer during a 400 h stability test of NiCoS_*x*_@Ni_3_S_2_/NF||NiCoS_*x*_@Ni_3_S_2_/NF via long-term chronoamperometry/chronopotentiometry

The comprehensive OWS performance was evaluated by constructing radar charts from HER and OER metrics (η_10_, TOF, Tafel slope, R_ct_, C_dl_). As shown in the Fig. 6a, b, NiCoS_*x*_@Ni_3_S_2_/NF exhibits the largest enclosed area, demonstrating superior HER and OER performance compared to the NiCo_2_O_4_@NiO/NF and Ni_3_S_2_/NF counterparts. Based on the bifunctional catalytic advantages of NiCoS_*x*_@Ni_3_S_2_/NF, a symmetric electrolyzer (NiCoS_*x*_@Ni_3_S_2_/NF||NiCoS_*x*_@Ni_3_S_2_/NF) was assembled to evaluate the OWS performance. The Faradaic efficiency (FE) of the overall water-splitting system was evaluated by a gas-collection (drainage) method. As shown in Fig. 6c, the measured H_2_/O_2_ volume ratio is close to the theoretical value of 2:1, indicating an FE approaching 100% and confirming that nearly all the input electrons are effectively utilized for water splitting, with negligible side reactions. To rigorously evaluate the catalytic performance, a commercial noble-metal-based electrolyzer using 20 wt% Pt/C/NF as the cathode and RuO_2_/NF as the anode (RuO_2_/NF||Pt/C/NF) was adopted as the benchmark for comparison. The NiCoS_*x*_@Ni_3_S_2_/NF||NiCoS_*x*_@Ni_3_S_2_/NF electrode exhibits an excellent OWS performance (1.54 V@10 mA cm^−2^, 2.10 V@1000 mA cm^−2^) comparable to that of Pt/C||RuO_2_ (Fig. 6d, e), outperforming other reported catalysts (Fig. 6f, Table S4). The electrolyzer with NiCoS_*x*_@Ni_3_S_2_/NF||NiCoS_*x*_@Ni_3_S_2_/NF symmetric electrodes shows excellent long-term stability at varied constant potentials. At 1.62, 1.79, and 1.96 V, the initial current densities are 50.3, 99.3, and 498.4 mA cm^−2^, with current retention of 95.6%, 94.1%, and 93.2%, respectively. The current density increases nearly tenfold with rising potential, realizing a superior activity-stability balance for practical applications (Fig. S39).

The protective role of the low-crystalline NiCoS_*x*_ shell against sulfide leaching during OER was quantitatively assessed by ICP-OES analysis of the sulfur content before and after 192 h of continuous electrolysis at varying current densities (Tables S5 –S7, Fig. S40). NiCoS_*x*_@Ni_3_S_2_/NF exhibits a relative sulfur loss of only 5.7%, markedly lower than the 18.5% observed for bare Ni_3_S_2_/NF at 10 mA cm^−2^. The core–shell architecture retains the majority of its sulfur content, with a loss of 9.6% at 100 mA cm^−2^, whereas Ni_3_S_2_/NF suffers a 23.9% decrease. Under an industrially relevant load of 500 mA cm^−2^, NiCoS_*x*_@Ni_3_S_2_/NF displays a sulfur loss of 11.8%, in sharp contrast to the 27.5% loss recorded for Ni_3_S_2_/NF.

During the 192 h OER durability tests conducted at varying current densities (10, 100, and 500 mA cm^−^^2^), the post-mortem ICP-MS analysis of electrolyte leaching concentrations and simultaneous monitoring of activity loss rates (Fig. S41) reveals a striking contrast in stability between NiCoS*ₓ*@Ni_3_S_2_/NF and Ni_3_S_2_/NF. NiCoS*ₓ*@Ni_3_S_2_/NF exhibited exceptional resistance to elemental leaching: under the harsh condition of 500 mA cm^−^^2^, the leaching concentrations of Ni and Co remain < 1 ppm, S leaching reaches only 2 ppm, and the corresponding activity loss rate is strictly controlled below 5%. This ultra-stable performance originates from the Ni–Co–S heterostructural synergy, which enhances metal-sulfur (M–S) bond energy, suppresses the oxidation of S^2^^−^ to SO_4_^2^^−^, and minimizes the formation of inert surface hydroxides, thereby preserving the integrity of active sites and the electronic structure of the M–S system. In stark contrast, the Co-free Ni_3_S_2_/NF, which possesses weaker M–S bond stability, exhibits significant leaching at 500 mA cm^−^^2^, with Ni and S concentrations rising to 3 and 8.5 ppm, respectively, and the activity loss rate increase sharply to 35%. These findings confirm that Co doping and the construction of the Ni–Co–S heterostructure effectively buffer interfacial oxidation stress, inhibit elemental leaching and surface reconstruction, and thus are responsible for the ultra-high activity and stability of NiCoS_*x*_@Ni_3_S_2_/NF even under high-current operating conditions.

To highlight the industrial application of anion exchange membrane water electrolysis (AEMWE) for hydrogen production, this study integrates a bifunctional electrode based on the core–shell structure NiCoS_*x*_@Ni_3_S_2_/NF into an electrolyzer (Fig. 6g). Polarization curves show that the electrode exhibits excellent catalytic activity in both the anode OER and the cathode HER. It achieves a high current density of 1.0 A cm^−^^2^ at a low overpotential of 2.12 V, demonstrating outstanding performance (Fig. 6h). Regarding stability, the electrode operates stability for 192 h, and even continuously for 400 h, showcasing the exceptional electrochemical durability provided by the core–shell structure (Fig. 6i).

More significantly, the nanorod morphology is essentially preserved after the OER reaction, with the formation of hydroxyl oxides on their surfaces (Fig. S42). XPS analysis reveals significant changes in the surface chemical states of the catalyst after HER and OER reactions: the appearance of a new peak in the Ni 2*p* and Co 2*p* spectrum after OER confirms the formation of NiOOH and CoOOH species; the Co 2*p* spectrum shows a 0.3 eV positive shift, indicating oxidation of Co species; In the S 2*p* spectrum, the intensity decreases after HER considering sulfur reduction/leaching, while peak broadening appears after OER attributing to sulfate formation. These results collectively reveal the dynamic evolution of the catalyst surface during the electrochemical reactions and their crucial impact on catalytic activity (Figs. S43, S44). This research provides a novel strategy for the development of efficient and stable industrial water electrolysis hydrogen production systems. Overall, the NiCoS_*x*_@Ni_3_S_2_/NF bifunctional electrode demonstrates not only excellent catalytic activity and stability but also strong potential for industrial applications, making it a promising solution for constructing efficient, stable and scalable AEMWE hydrogen production systems.

To contextualize the durability of NiCoS_*x*_@Ni_3_S_2_/NF, we systematically benchmarked its performance against recent state-of-the-art TMS electrocatalysts as summarized in Table S8. The survey shows that most reported sulfide-based OWS catalysts achieve long-term stability primarily at low-to-moderate current densities (≤ 100 ~ 500 mA cm^−2^) for operation times ranging from several h to 200 h. Representative examples include Co_9_S_8_–NiCo_2_S_4_/N-rGO, which maintains negligible voltage growth for 15 h at 10 ~ 100 mA cm^−2^, and NiCo_2_S_4_@Ce-NiFe LDH/CeO_2_ nanoarrays, which operate stably for 100 h at 50 ~ 100 mA cm^−2^. Only a few catalysts, such as CeO_2_/NiCo_2_S_4_, demonstrate > 200 h durability at 500 mA cm^−2^. In contrast, NiCoS_*x*_@Ni_3_S_2_/NF exhibits continuous OWS operation for 192 h under stepwise current densities, reaching 1.0 A cm^−2^ in 1.0 M KOH-approaching industrially relevant regimes while maintaining significantly higher sulfur retention compared with bare Ni_3_S_2_, as confirmed by ICP-OES analysis. The superior durability under harsher loads than most reported studies, results from the synergy of a low-crystalline NiCoS*ₓ* shell, crystalline Ni_3_S_2_ core and a ΔΦ-induced BEF. These features collectively inhibit structural degradation and slow sulfur leaching during extended OER operation.

### Density Functional Theory Calculation

**Fig. 7:**
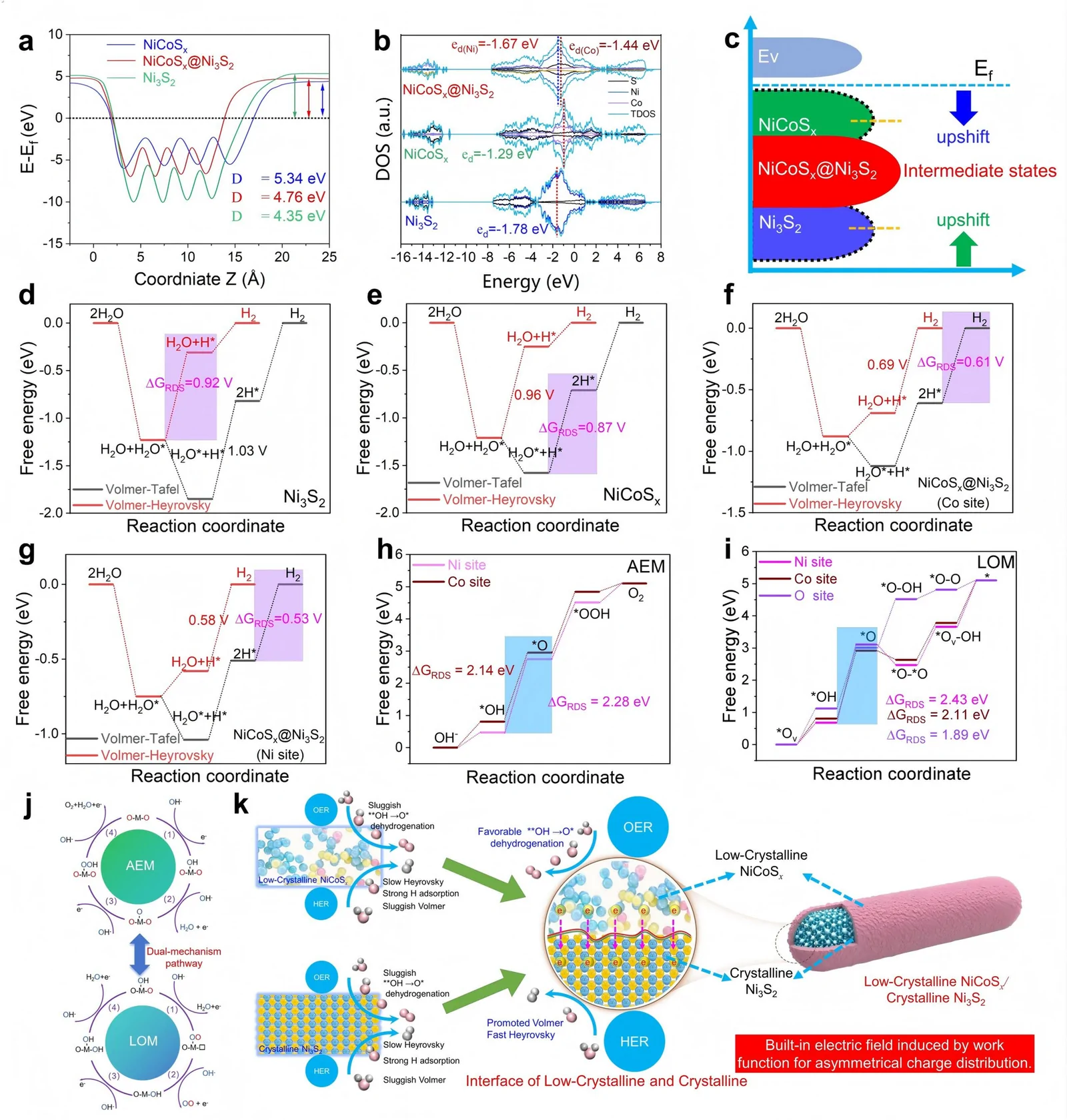
The mechanism of simulation calculation. **a** ΔΦ of the Ni_3_S_2_, NiCoS_*x*_, and NiCoS_*x*_@Ni_3_S_2_. **b** DOSs and **c** schematic diagram of d-band center of Ni_3_S_2_, NiCoS_*x*_, and NiCoS_*x*_@Ni_3_S_2_. Free energy profiles of hydrogen evolution reaction via Volmer-Tafel and Volmer-Heyrovsky pathways on: **d** Ni_3_S_2_, **e** NiCoS_*x*_, **f** NiCoS_*x*_@Ni_3_S_2_ Ni site, and **g** NiCoS_*x*_@Ni_3_S_2_ Co site. Free energy profiles of the OER via the **h** AEM mechanism and **i** LOM mechanism. **j** Schematic illustration of the dual-mechanism pathways AEM and LOM for oxygen evolution reaction. **k** Schematic illustration of the catalytic mechanism for the enhanced OER and HER at the NiCoS_*x*_@Ni_3_S_2_ heterointerface

Density functional theory (DFT) calculations were performed to gain deeper insights into the catalytic mechanism of NiCoS_*x*_ and Ni_3_S_2_. Theoretical models of Ni_3_S_2_, NiCoS_*x*_ and NiCoS_*x*_@Ni_3_S_2_ were constructed and optimized (Fig. S45). The interfacial charge polarization revealed by charge density difference optimizes the intermediate adsorption at the heterostructure (Fig. S46a, b). To probe the interfacial charge dynamics in the heterostructure, the ΔΦ for Ni_3_S_2_ (4.35 eV), NiCoS_*x*_ (5.34 eV) and NiCoS_*x*_@Ni_3_S_2_ (4.76 eV), reveals that BEF governs electron transfer across the heterogeneous interface (Fig. 7a). These values indicate that the heterogeneous interface layer gives rise to an asymmetric charge distribution generated by the BEF results. This BEF facilitates the easy transfer of electrons from electron-rich NiCoS_*x*_ to electron-deficient Ni_3_S_2_, until the entire heterogeneous interface reaches a charge balance. This result is consistent with that of the UPS analysis (Fig. 1k).

Notably, the electron-rich Ni_3_S_2_ surface enhances H*/H_2_O adsorption, and the electron-deficient NiCoS_*x*_ interface lowers the Gibbs free barrier for H*/*OH intermediate conversion. This synergistic electronic modulation optimizes the HER/OER kinetics, significantly boosting catalytic OWS performance. Molecular orbital theory elucidates the electronic coupling effects of Ni, S, and Co species in the NiCoS_*x*_@Ni_3_S_2_/NF heterostructure, revealing an enhanced electron transfer mechanism that leads to its superior catalytic performance. The low-spin state configuration (t_2g_^6^e_2g_^2^) of Ni^2+^ indicates complete occupation of the π-symmetric d-orbitals, resulting in significant electron–electron (e-e) repulsion between the bridging Ni^2+^ and S^2−^ species. Conversely, Co^2+^ exhibits a high-spin state configuration (t_2g_^3^e_g_^4^), characterized by unpaired electrons in the π-symmetric d-orbitals, facilitating electronic interactions with S^2−^ through bridging coordination (Fig. S47a). Therefore, the chemical bonding between NiCoS_*x*_ and Ni_3_S_2_ at the Ni–S–Co interface intensifies the electron–electron repulsion between Co^2+^ and S^2−^ species, thereby facilitating the formation of an extended π-electron cloud bridge between Ni^2+^ and S^2−^ species. This dynamic process contributes to the formation of Ni–S–Co by the electron coupling effect between Co^2+^, S^2−^, and Ni^2+^, realizing the electron transfer from NiCoS_*x*_ to Ni_3_S_2_ (Fig. S47b). Density of states (DOS) analysis was performed on Ni_3_S_2_, NiCoS_*x*_ and NiCoS_*x*_@Ni_3_S_2_ models to elucidate their catalytic mechanism. The DOS near the E_f_ of the NiCoS_*x*_@Ni_3_S_2_ is primarily contributed by the 3*d* orbitals of Co and Ni, rather than the 2*p* orbital of sulfur, suggesting that the adsorption behavior of reaction intermediates on the catalyst surface is predominantly governed by the electronic interaction mechanism between Co atoms and Ni atoms. As shown in the Fig. 7b, the d-band centers of Ni/Co active sites (− 1.67/− 1.44 eV) in the NiCoS_*x*_@Ni_3_S_2_ are in between those of Ni_3_S_2_ (− 1.78 eV) and NiCoS_*x*_ (− 1.29 eV), indicating that the adsorption energy of HER/OER intermediates in NiCoS_*x*_@Ni_3_S_2_ is neither too strong nor too weak, thereby enhancing the electrocatalytic activity (Fig. 7c).

To explore the regulation mechanism of catalyst composition, interface microstructure and active site configuration on the kinetics of the HER, this study systematically characterizes the free energy evolution of Ni_3_S_2_, NiCoS_*x*_ and NiCoS_*x*_@Ni_3_S_2_ (Co and Ni active sites) in the HER process based on first-principles calculations. The results show that Ni_3_S_2_ mainly catalyzes HER via the Volmer-Heyrovsky pathway, with a high rate-determining step (RDS) barrier of 0.92 eV (Fig. 7d). Co doping improves the catalytic activity of NiCoS_*x*_ by rearranging the electronic structure and adjusting hydrogen adsorption energy, lowering the RDS barrier to 0.87 eV (Fig. 7e). In the NiCoS_*x*_@Ni_3_S_2_ heterojunction, Co doping induces electronic coupling and synergistic effects, reducing the RDS barrier further to 0.61 eV at the Co site and 0.53 eV at the Ni site (Fig. 7f, g). This shifts the HER reaction to follow the Volmer-Tafel pathway, demonstrating excellent intrinsic activity. These results demonstrate that the synergistic strategy of heterostructure construction and Co doping effectively overcomes performance bottlenecks in sulfide-based catalysts, with Ni sites in the NiCoS_*x*_@Ni_3_S_2_ heterojunction serving as high-activity centers.

The OER in KOH electrolyte involves a four-electron “energy-climbing” process, and the activity is closely related to the magnitude of the Gibbs free energy (ΔG) of the intermediates on the material surface. This study constructed seven DFT models, including Ni_3_S_2_, Ni_3_S_2_/NiOOH, NiCoS_*x*_/NiOOH, NiCoS_*x*_/CoOOH, NiCoS_*x*_@Ni_3_S_2_, NiCoS_*x*_@Ni_3_S_2_/NiOOH, NiCoS_*x*_@Ni_3_S_2_/CoOOH (Fig. S45) to explore the regulatory mechanism of interface reconstruction on catalytic kinetics. DFT free energy calculations reveal a stepwise optimization mechanism for OER kinetics driven by heterojunction interface reconstruction: Ni_3_S_2_ exhibits strong adsorption of *OH/*O intermediates due to the low electronegativity of sulfur atoms, resulting in a high energy barrier of 3.37 eV (ΔG_3_) for the RDS OH → O step (Fig. S48a). In contrast, the NiCoS_*x*_/NiOOH heterojunction lowers the energy barrier to 2.39 eV (ΔG_2_), due to the coupling of the interface S–O bond and the oxygen-evolving properties of NiOOH, confirming the crucial role of the reconstructed layer (Fig. S48b, c). Upon further cobalt doping, the high-spin state of Co^3+^ (t_2g_^3^e_g_^4^) in NiCoS_*x*_@Ni_3_S_2_/NiOOH optimizes the OOH adsorption configuration, lowering the rate-determining O → *OOH energy barrier to 2.09 eV, lower than that at the Ni site (2.28 eV). This advantage stems from the defect-rich low-crystallinity NiCoS*ₓ* layer (ECSA = 25.1 mF cm^−^^2^) and BEF-driven charge transfer (R_st_ = 3.1 Ω). Ultimately, the theoretical energy barrier aligns closely with the experimental overpotential (229 mV@10 mA cm^−^^2^), confirming that the pre-reconstructed sulfide interface continuously enhances the kinetics of the reconstructed layer through electronic coupling.

To explore the catalytic dynamics of the catalyst in the OER, this study uses first-principles calculations to compare the free energy evolution paths of the AEM and the LOM. The results show that the OER reaction primarily follows the LOM path, with a significantly lower energy barrier compared to the AEM path. In the AEM path, the RDS energy barriers for Ni and Co sites are 2.14 and 2.28 eV, respectively (Fig. 7h). In the LOM path, the energy barrier for the O site is the lowest at 1.89 eV, with 2.11 and 2.43 eV for the Ni and Co sites, respectively (Fig. 7i). This indicates that LOM is the dominant reaction pathway, with the O site playing a key role in the LOM path (Fig. 7j). These findings provide theoretical support for designing efficient OER catalysts by regulating the participation of lattice oxygen.

The enhanced HER and OER activities of the NiCoS*ₓ*@Ni_3_S_2_/NF catalyst originate from the spontaneous electron transfer induced by the work function difference (ΔΦ) at the NiCoS_*x*_/Ni_3_S_2_ interface, which generates a robust BEF with asymmetric charge distribution (Fig. 7k). This BEF significantly optimizes reaction kinetics: (1) For HER, it lowers the energy barrier for water dissociation (Volmer step: H_2_O → H* + OH^−^) and facilitates hydrogen adsorption/desorption (Heyrovsky step). Specifically, H* preferentially adsorbs on the negatively charged Ni_3_S_2_ side, where it combines with another H* and electrons to form H_2_. (2) For OER, the BEF reduces the activation energy for O–O bond formation and accelerates the RDS (*OH → *O) by stabilizing intermediates on the positively charged NiCoS_*x*_. Therefore, the crystalline Ni_3_S_2_ nanorods tuned by low-crystalline NiCoS_*x*_ with BEF enable superior dual functionality for efficient overall water splitting.

## Conclusions

In summary, we synthesize a novel electrocatalyst of NiCoS_*x*_@Ni_3_S_2_ by making use of low-crystalline NiCoS_*x*_ to tune crystalline Ni_3_S_2_ nanorods via ion-exchange strategy, where the low-crystalline NiCoS_*x*_ shell endows the catalyst with multiple synergistic advantages: enriched active sites from atomic disorder, optimized electron transfer from the built-in electric field induced by work function difference, dynamic surface reconstruction into high-activity NiCoOOH, and enhanced structural stability via suppressing sulfide leaching. The optimized NiCoS_*x*_@Ni_3_S_2_ achieves low HER/OER overpotentials of 346/520 mV@1000 mA cm^−2^, evidenced by an ultralow cell voltage of 2.10 V@1000 mA cm^−2^ for OWS and remarkable long-term stability for 400 h at various current densities. Based on in situ Raman, XPS and DFT simulations, the superior catalytic performance and exceptional stability are ascribed to the following factors: (**i**) The inner layer is high crystalline Ni_3_S_2_, exhibiting exceptional electrical conductivity, while the outer surface layer is composed of low-crystalline NiCoS_*x*_ with abundant defect sites; (**ii**) The BEF in NiCoS_*x*_@Ni_3_S_2_ accelerates water dissociation, optimizes hydrogen adsorption/desorption for HER, and reduces the reaction energy barrier of *O → *OOH for OER, thereby promoting the overall catalytic kinetics of water splitting; (**iii**) The low-crystalline NiCoS_*x*_ layer with the strong Ni–S–Co interfacial bridge acts an armor to suppress the sulfide loss, resulting in high catalytic activity and stability. This work paves a way to fabricate sulfur-based electrocatalysts with high yet balanced activity and stability for OWS via interface engineering strategy.

## Supplementary Information

Below is the link to the electronic supplementary material.

Supplementary file1 (DOCX 7633 kb)
