# Supplementary material for: Crystalline Ni3S2 Nanorods Tuned by Low-Crystalline NiCoSx with Built-In Electric Field for Efficient Overall Water Splitting
Source: Nanomicro Lett. 2026 Mar 26;18:304. doi: 10.1007/s40820-026-02151-6 (PMC13022221; doi:10.1007/s40820-026-02151-6)
Supplement: Supplementary file 1 — Supplementary file1 (DOCX 7633 kb) [file 40820_2026_2151_MOESM1_ESM.docx]

Supporting Information for

**Crystalline Ni_3_S_2_ Nanorods Tuned by Low-Crystalline NiCoS*****_x_* with Built-In Electric Field for Efficient Overall Water Splitting**

Neng Chen^1^, Jun He^1^, Hongqiang Li^1^, Dedong Jia^1^, Haoran Qian^1^, Huan Pang^2,^ *, Jieshan Qiu^3,^ *, Yongfeng Li^4^, Xiaojun He^1,^ *

^1^ School of Chemistry and Chemical Engineering; Anhui Province Key Laboratory of Coal Clean Conversion and Low Carbon Utilization; Anhui Province Key Laboratory of Efficient Conversion and Solid-State Storage of Hydrogen & Electricity; Key Laboratory of Metallurgical Emission Reduction and Resources Recycling, Ministry of Education, Anhui University of Technology, Ma’anshan, Anhui 243002, P. R. China

^2^ Institute of Innovation Materials and Energy, School of Chemistry and Chemical Engineering, Yangzhou University, Yangzhou, 225002, P. R. China

^3^ College of Chemical Engineering, State Key Laboratory of Chemical Resource Engineering, Beijing University of Chemical Technology, Beijing, 100029, P. R. China

^4^ State Key Laboratory of Heavy Oil Processing, China University of Petroleum, Beijing, 102249, P. R. China

*Corresponding authors. E-mail: [panghuan@yzu.edu.cn](mailto:panghuan@yzu.edu.cn) (Huan Pang); [qiujs@mail.buct.edu.cn](mailto:qiujs@mail.buct.edu.cn) (Jieshan Qiu); [xjhe@ahut.edu.cn](mailto:xjhe@ahut.edu.cn) (Xiaojun He)

**S1 Experimental section**

**S1.1 Characterization of samples**

The crystal composition of the catalysts was identified by X-ray diffraction technique (XRD, Rigaku MiniFlex 600 with CuKα radiation (λ = 1.54056 Å)). The morphology and microstructure of the catalysts were observed by scanning electron microscope (SEM, FEI Quanta 200F) and transmission electron microscopy (TEM, Tecnai G2 F20). Chemical state of the catalysts was examined by X-ray photoelectron spectroscopy (XPS, Thermo Fisher). The electron para-magnetic resonance (EPR) analysis was performed utilizing a Bruker A200. UPS measurements were conducted by using a Thermo Fisher Scientific Escalab 250Xi instrument. The graphitization degree and defects of the catalysts were evaluated by Raman microscopy (Horiba Jobin Yvon). The measurement of work function (WF) was accomplished by Ultraviolet Photoelectron Spectroscopy (UPS) technique. A helium discharge lamp with a photon energy of 21.22 eV was employed as the excitation source. During the analysis of the UPS spectrum, the secondary electron cutoff edge (E_cutoff_) was precisely identified at the high binding energy. Subsequently, the formula of WF = 21.22 eV - |E_cutoff_ - EF| was utilized to calculate the work function, and EF stands for the Fermi level with 0 eV baseline.

**S1.2 In situ Raman and XPS measurement**

The in-situ three-electrode electrochemical cell was conducted by employing CHI660E electrochemical workstation with Ni_3_S_2_/NiCoS*_x_*/NF as the working electrode, Hg/HgO electrode as the reference electrode, and graphite rod as the counter electrode. In situ Raman spectra was conducted at various applied potentials from 1.35 to −0.5 V vs. RHE with intervals of 0.01 V for electrooxidation process. Additionally, in situ XPS of Ni_3_S_2_/NF and NiCoS*_x_*@Ni_3_S_2_/NF was performed at different potentials from 1.1 to 1.6 V vs. RHE with intervals of 0.1 V.

**S1.3 X-ray absorption spectra**

The X-ray absorption spectroscopy (XAS) measurements were conducted at the BL01C1 beamline of the Shanghai Synchrotron Radiation Facility (SSRF). The typical energy of the storage ring was 1.5 GeV, with an electron current of 180 mA under top-up mode. The incident white light was monochromatized using a Si (111) double-crystal monochromator and calibrated with standard metal foils to ensure energy accuracy. For sample preparation, the samples were pressed into thin slices and positioned at a 90° angle to the incident beam in a sample holder. The XAS spectra were collected in transmission mode using two ion chambers for signal detection. The Ni K-edge spectra were scanned in the range of 8200 to 8700 eV with a step size of 0.1 eV, while the Co K-edge spectra were scanned in the range of 7500 to 8000 eV with the same step size. XANES (X-ray absorption near-edge structure) and EXAFS (Extended X-ray absorption fine structure) data were analyzed using the Athena and Artemis software. Key parameters, including coordination number (N), bond length (R in Å), and the Debye-Waller factor (σ²), were extracted from the EXAFS data to obtain detailed structural information. To further investigate the nature of first-shell backscattering atoms and probe both light and heavy scatterers, wavelet transform (WT) analysis was employed. This qualitative analysis method focused on the characteristics of the backscattering atoms and bond lengths, benefiting from the high resolution in both the wavenumber k and radial distribution function R, complementing the limitations of Fourier transform (FT) analysis. A Morlet wavelet was selected as the mother wavelet, with parameters (η = 8, σ = 1) to optimize the resolution in the wave vector k for better data analysis.

**S1.4 Electrochemical measurements****.**

The electrochemical performance of the electrocatalysts was collected by the electrochemical workstation (CHI 760E, Shanghai) in 1.0 M KOH solution on a standard three electrode system with as-synthesized composite materials (1×1 cm^2^) as the working electrode, Hg/HgO electrode and the graphite rod as the reference electrode and counter electrode, respectively. The polarization curves for HER and OER were obtained by linear sweep voltammetry (LSV) at a scan rate of 2 mV s^−1^, which ensures quasi-steady-state conditions while effectively minimizing the influence of capacitive currents. An 80% iR compensation was applied to all polarization data to correct for solution resistance, thereby ensuring that the obtained electrochemical performance accurately reflects the intrinsic catalytic activity of the samples. Prior to electrochemical measurements, a pre-activation process was performed to stabilize the electrode–electrolyte interface and ensure measurement reproducibility. Specifically, the catalyst-coated electrodes were subjected to continuous cyclic voltammetry (CV) scans within the relevant potential window of each reaction at a scan rate of 50 mV s^−1^ for 20 cycles until the polarization curves became stable. For HER, the potential range was set from −0.2 to 0.1 V vs. RHE, while for OER, the potential range was 1.0 to 1.6 V vs. RHE. After this pre-activation process, all polarization and durability measurements were conducted. All electrochemical measurements were conducted at room temperature (approximately 25 ± 2 °C) under ambient laboratory conditions, without external temperature control. The electrolyte (e.g., 1.0 M KOH aqueous solution) was bubbled with high-purity N_2_ (99.999%) continuously for at least 30 min to remove dissolved O_2_. The N_2_ flow was maintained to the electrolyte throughout the electrochemical measurements to prevent re-dissolution of atmospheric oxygen.

The electrochemical active surface area (ECSA) was in proportion to the double-layer capacitance (C_dl_) obtained by cyclic voltammogram (CV) at various scan rates of 20, 40, 60, 80, 100, and 120 mV s^−1^. The Tafel slope by steady state polarization method was employed to obtain the current density at the 200 s of chronoamperometric (CA) response at an interval potential of 0.050 V in the catalytic turnover area (the potential range of HER is −0.026 ~ −0.476 V, and that of OER is 1.424 ~ 1.924 V). The Tafel slope of the steady state polarization curve was calculated by using the current density in the steady state and the corresponding overpotential compensated by the resistance. Electrochemical impedance spectroscopy (EIS) was collected in the potentiation mode at a potential of −0.15 V (vs. RHE) from 10^−1^ Hz to 10^5^ Hz. The amplitude of the alternating voltage was −0.17 V vs. RHE for HER and 1.58 V vs. RHE for OER.

**S1.5 Calculation of the number of active sites**

The active sites (n) of the catalysts was measured by CV in 1.0 M KOH solution (pH = 14) at a scan rate of 50 mV s^−1^ with potential from 0.8 to 1.6 V for OER and −0.2 to 0.6 V for HER (vs. RHE). The area of the integral curve was used to calculate the absolute value of the voltammetric charge. The equation (S1) was used to calculate the n:

| $n=\frac{Q}{mF}=\frac{It}{mF}=\frac{IV}{mF\upsilon}$ | (S1) |
| --- | --- |

Where, *Q* is the total voltammetric charge and *F* is the faraday constant (C mol^−1^), *I* is the current (A), *t* is the time (s) and *V* is the potential (V) and *ʋ* is the scan rate (V s^−1^).

**S1.6 Calculation of turnover frequency (TOF)**

The TOF for OER and HER was determined by using equation (S2):

| $\text{TOF}=\frac{jA}{nmF}$ | (S2) |
| --- | --- |

Where, *j* is the current in ampere (A) obtained during LSV measurement, *A* is geometric electrode area (1 cm^2^), *F* is the Faraday constant (96485 C mol^−1^), *m* is the number of electrons transferred to generate one molecule of the product (*m* = 4 for OER, and *m* = 2 for HER) and *n* is the number of active sites, respectively (mol^−1^).

**S1.7 Membrane Pre-treatment and Electrolyzer Assembly**

Prior to fabricating the membrane-electrode assembly (MEA), the anion exchange membrane (Fumasep FAA-3-50) was subjected to preprocessing in a 0.5 M potassium hydroxide (KOH) alkaline solution for at least 18 hours. After this pretreatment stage, the membrane was thoroughly rinsed with deionized (DI) water, which promoted its conversion into the hydroxyl form through an ion exchange process. To preclude CO₂-induced contamination that could deteriorate the membrane’s electrochemical performance, the preprocessed membrane was stored in a humidified, CO_2_-free environment. The preprocessed membrane was sandwiched between two NiCoS*_x_*@Ni_3_S_2_/NF electrodes, which functioned as the anode and cathode, respectively. The resulting stacked configuration was then hot-pressed at 40 °C under a pressure of 5 MPa for a duration of 3 minutes. Subsequent to hot-pressing, the two NiCoS*_x_*@Ni_3_S_2_/NF electrodes and the membrane were integrated to form the MEA. This MEA was further assembled with auxiliary components, such as titanium flow endplates and polytetrafluoroethylene (PTFE) gaskets, to finalize the complete electrolyzer system. For comparative analysis, a lab-fabricated anion exchange membrane water electrolyzer (AEMWE) cell was constructed using RuO_2_ and 20 wt.% Pt/C as catalysts, with their respective loadings on the membrane regulated at 2 mg cm^−2^ and 1 mg cm^−2^. The active area of the single electrolytic cell was fixed at 2 cm × 2 cm.

**S1.8 DFT calculations**

In this study, all density functional theory (DFT) computations were carried out using the Vienna Ab initio Simulation Package (VASP). The calculations were carried out by employing the projector augmented wave (PAW) approach within the framework of first-principles DFT.

The exchange-correlation functional was described by the Perdew-Burke-Ernzerhof (PBE-GGA) functional within the generalized gradient approximation (GGA), which exhibits excellent reliability in predicting the structural, electronic properties, and catalytic behaviors of transition metal sulfides (TMSs). Considering the non-negligible influence of van der Waals forces (dispersion interactions) on the adsorption behavior of reaction intermediates such as *OH, *O, and *OOH on the catalyst surface, the semi-empirical DFT-D3 correction method proposed by Grimme was introduced. The core electrons were treated with effective core potentials (ECP) combined with the double numerical plus polarization (DNP v4.4) basis set, balancing computational accuracy and efficiency by freezing core electrons while retaining the flexibility of valence electrons.

Regarding the computational models, the surface structures of Ni₃S₂ and NiCoSₓ were both constructed based on their experimentally confirmed crystal phases. The Ni₃S₂ (101) surface was selected as the model surface because it was verified as the thermodynamically most stable facet by surface energy calculations, and X-ray diffraction (XRD) and transmission electron microscopy (TEM) characterizations confirmed it as the dominant exposed facet on the catalyst surface. For NiCoS*_x_*, the (220) surface was chosen in consideration of the lattice distortion induced by Co doping (verified by XRD peak shift and high-resolution transmission electron microscopy (HRTEM) lattice fringe analysis). The Co doping amount in the model (Ni:Co molar ratio of 3:1) was consistent with the inductively coupled plasma mass spectrometry (ICP-MS) test results of the synthesized catalyst.

A 15 Å vacuum layer was set along the z-axis for the supercell models of both surfaces to eliminate spurious interactions between periodic images. Structural relaxation was performed until the residual force on each atom was less than 0.02 eV Å⁻^1^ and the total energy converged to 10⁻^5^ eV per unit cell. For Brillouin zone integration, a 2×2×1 Monkhorst-Pack k-point mesh was used for structural optimization, while a denser 4×4×1 k-point mesh was adopted for density of states (DOS) and partial density of states (PDOS) calculations to ensure sufficient sampling of the electronic structure. Charge density difference, work function, and adsorption energies were calculated to elucidate interfacial electronic modulation and reaction energetics.

For each elementary step, the Gibbs reaction free energy ΔG is defined as the difference between free energies of the initial and final states and is given by the expression (S3):

ΔG =ΔE +ΔEZPE-TΔS (S3)

Where ΔE is the reaction energy of reactant and product molecules adsorbed on catalyst surface, obtained from DFT calculations; ΔEZPE and ΔS are the change in zero-point energies and entropy due to the reaction.

**Supplementary Figures and Tables**

**
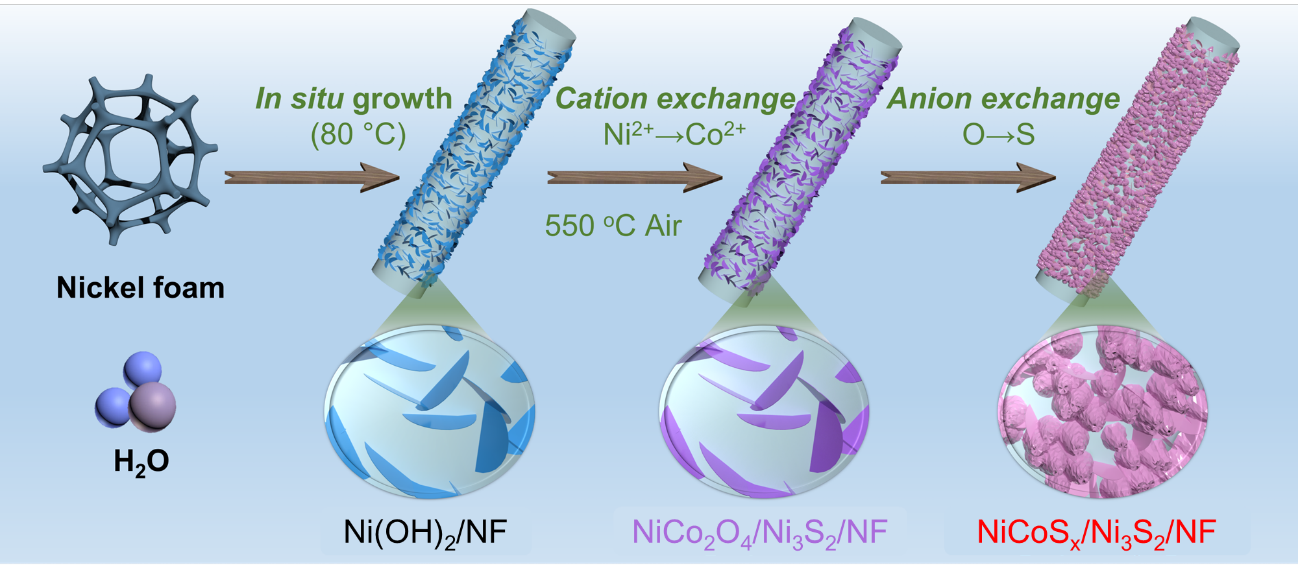
**

**Fig. S1** Schematic diagram of the manufacturing procedure of NiCoS*_x_*@Ni_3_S_2_/NF


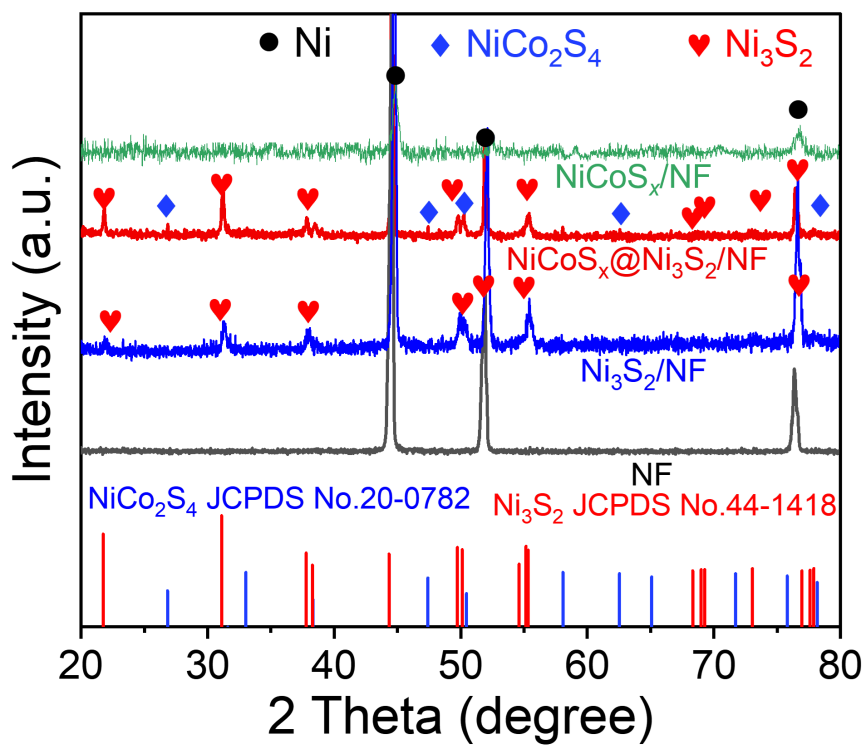


**Fig. S2** XRD patterns of Ni_3_S_2_/NF, NiCoS*_x_*@Ni_3_S_2_/NF, and NiCoS*_x_*/NF


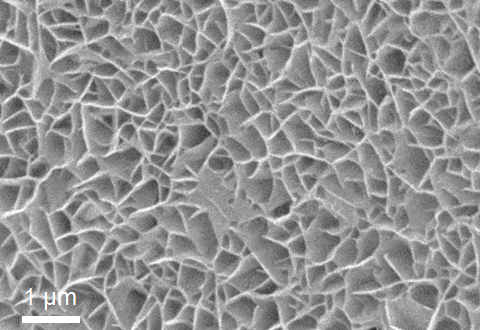


**Fig. S3** The SEM image of NiCoO/NF


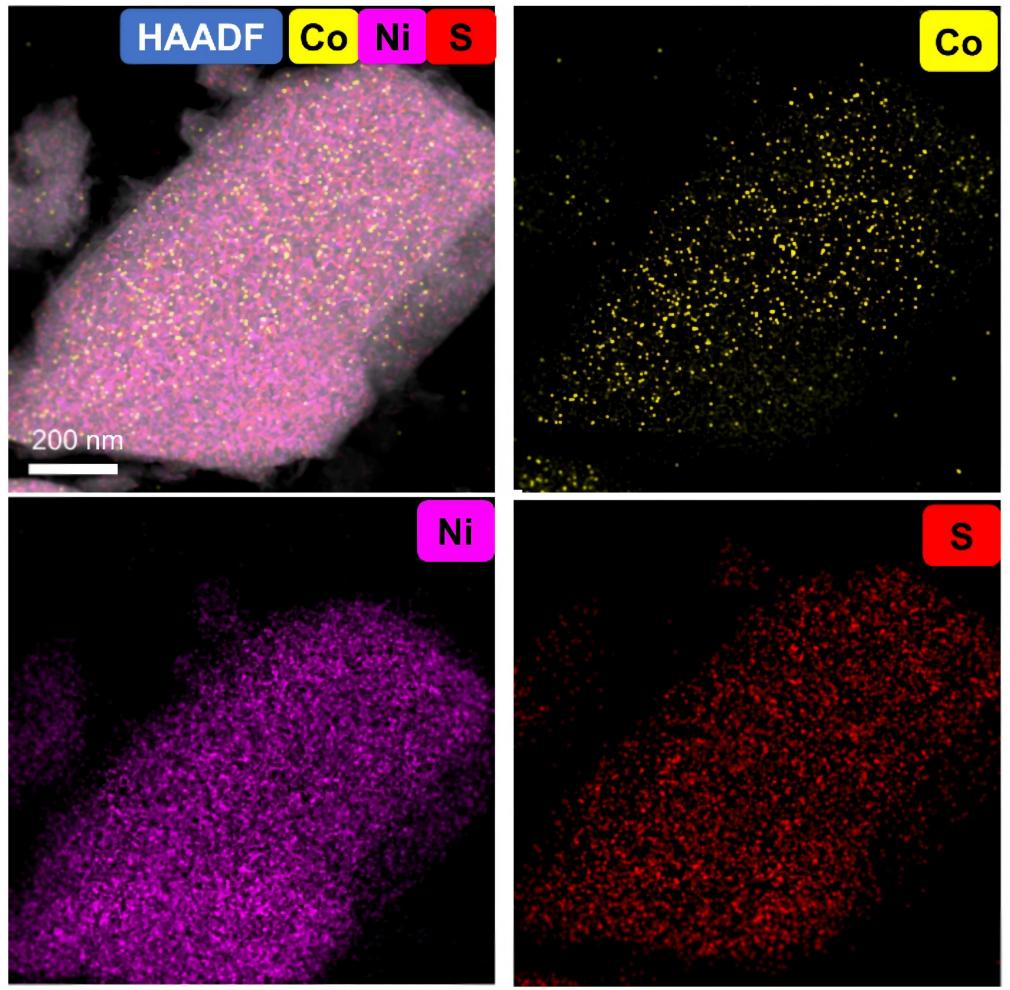


**Fig. S4** The EDX elemental mappings of NiCoS*_x_*@Ni_3_S_2_ for Co, Ni and S element


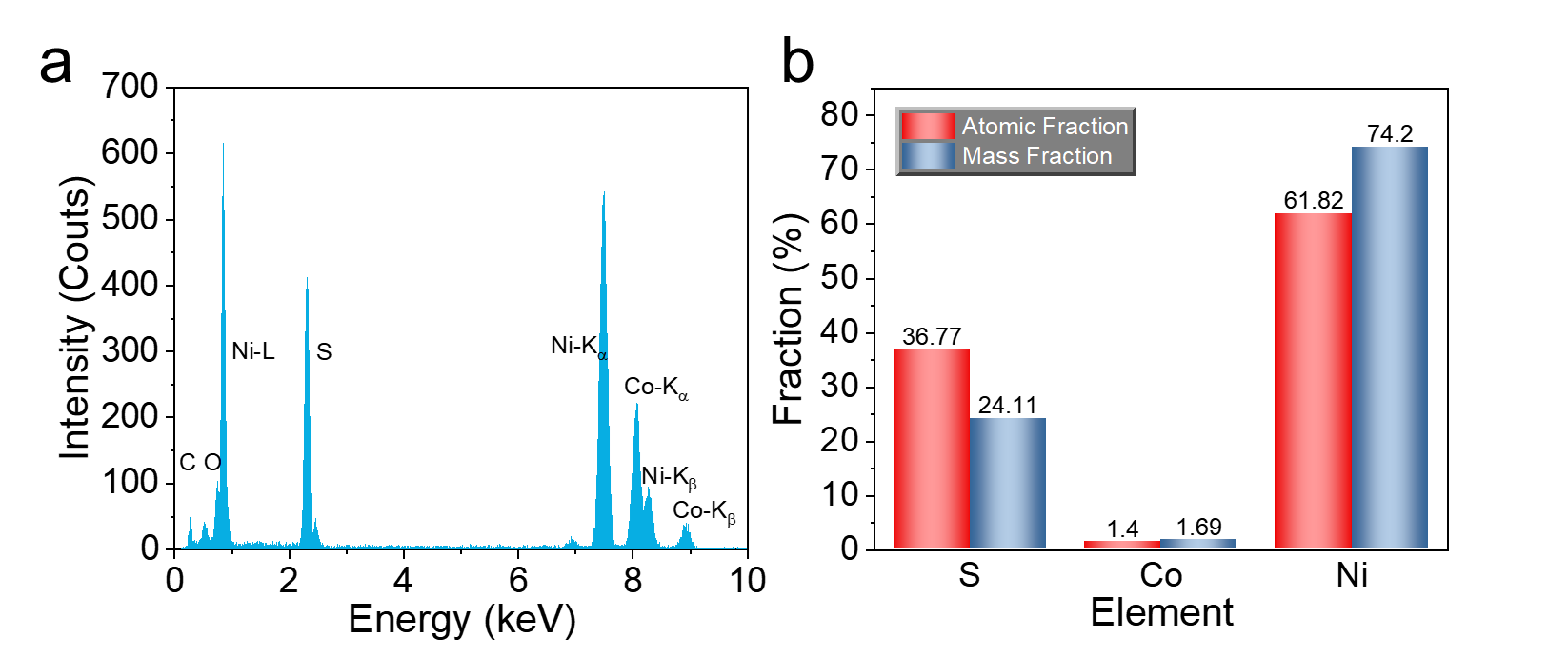


**Fig. S5 a**, **b** Elemental distribution of the EDX energy spectrum for NiCoS*_x_*@Ni_3_S_2_


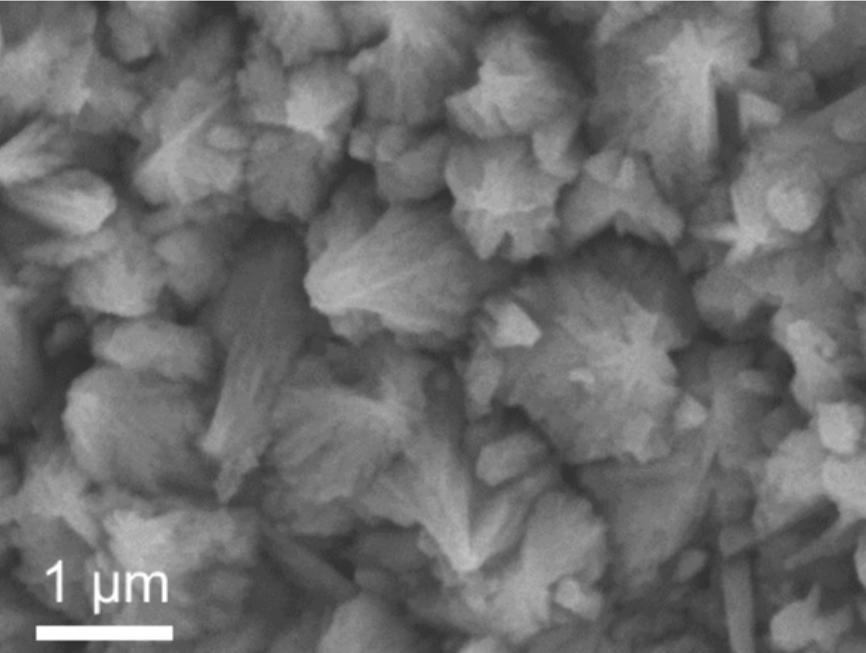


**Fig. S6** The SEM image of Ni_3_S_2_/NF


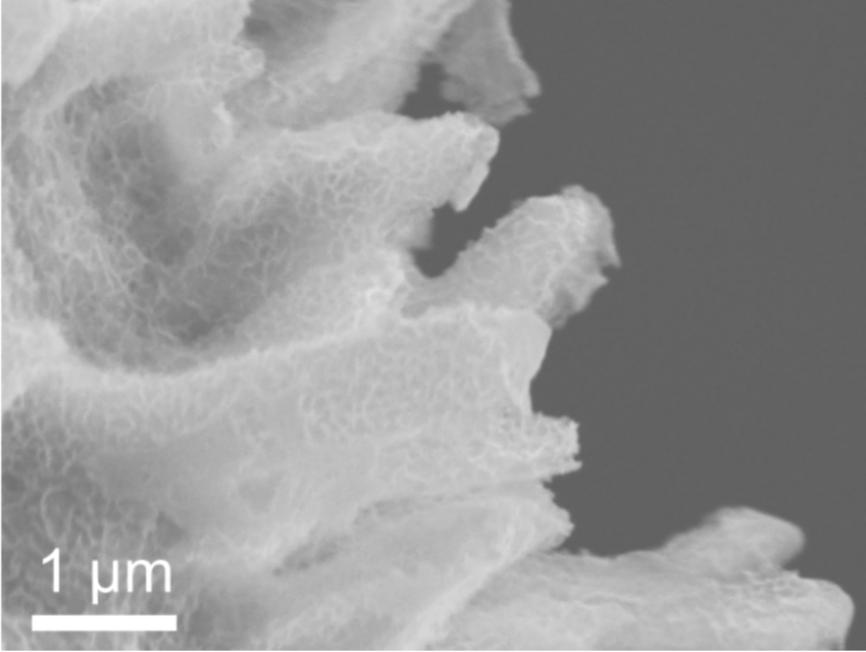


**Fig. S7** The SEM image of NiCoS*_x_*/NF


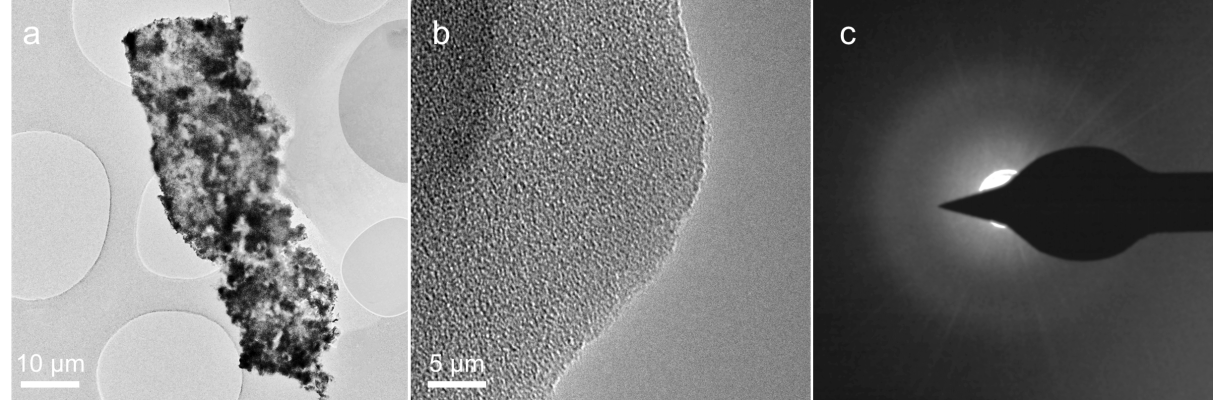


**Fig. S8** **a** TEM image, **b** HRTEM image, **c** SAED pattern of NiCoS*_x_*/NF


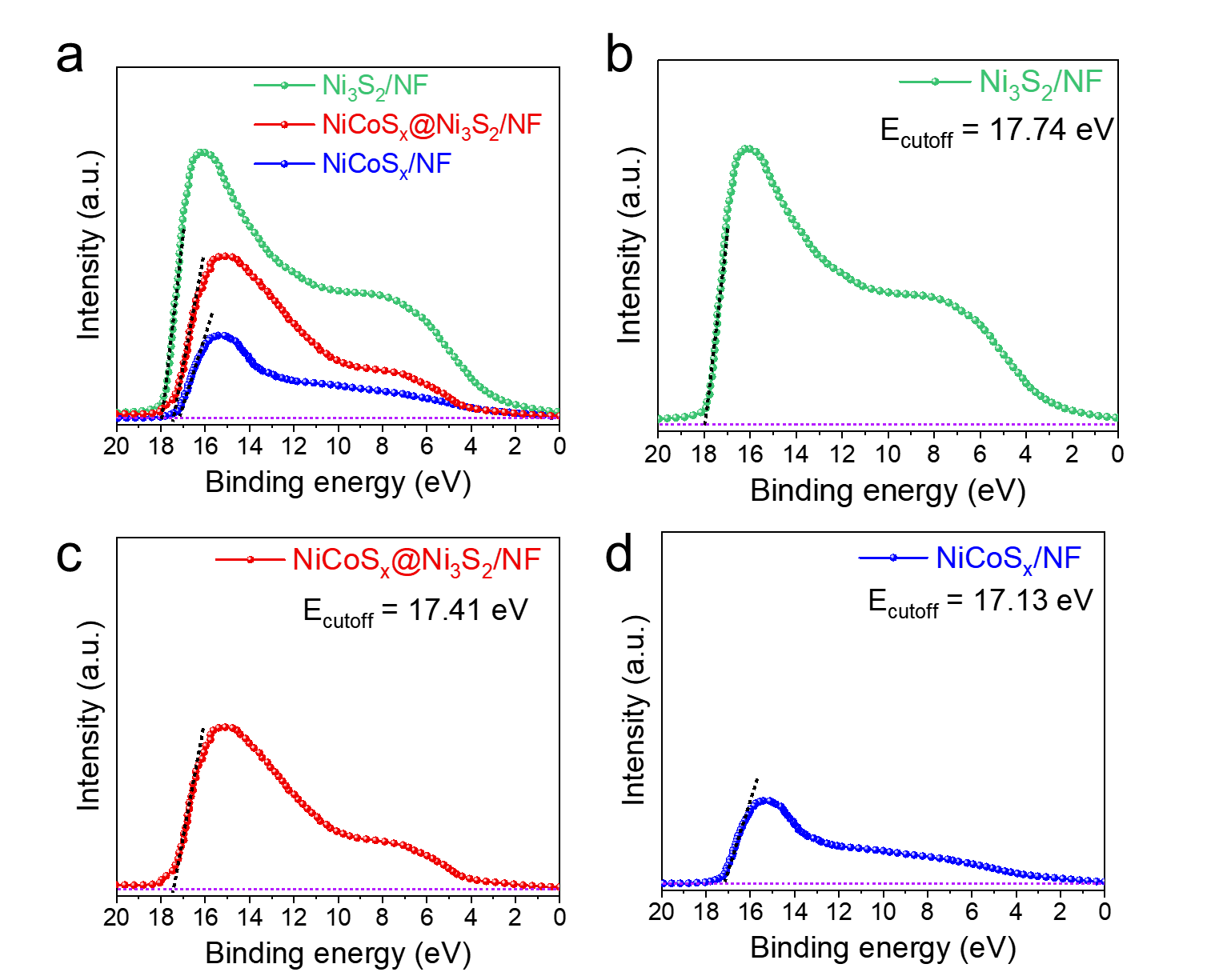


**Fig. S9** UPS spectra: **a** Comparison of all samples, **b** Ni_3_S_2_/NF, **c** NiCoS*_x_*@Ni_3_S_2_/NF, **d** NiCoS*_x_*/NF


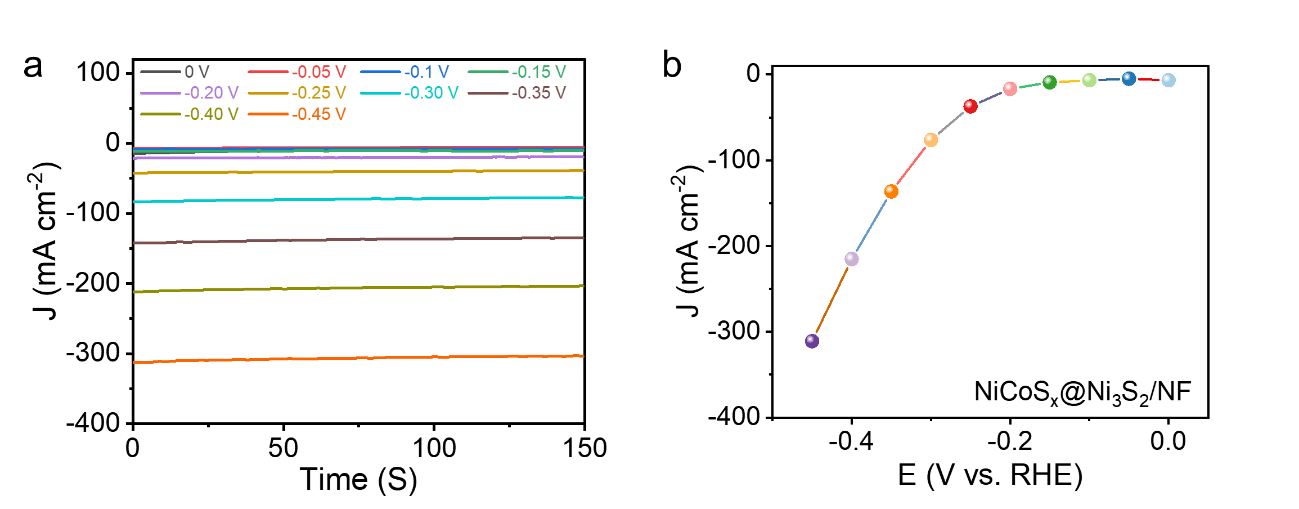


**Fig. S10 a** The chronoamperometric stability of NiCoS*_x_*@Ni_3_S_2_/NF for HER in 1.0 M KOH solution at applied potentials from 0 to −0.45 V (vs. RHE). **b** The plot of sampled HER by the steady-state polarization method


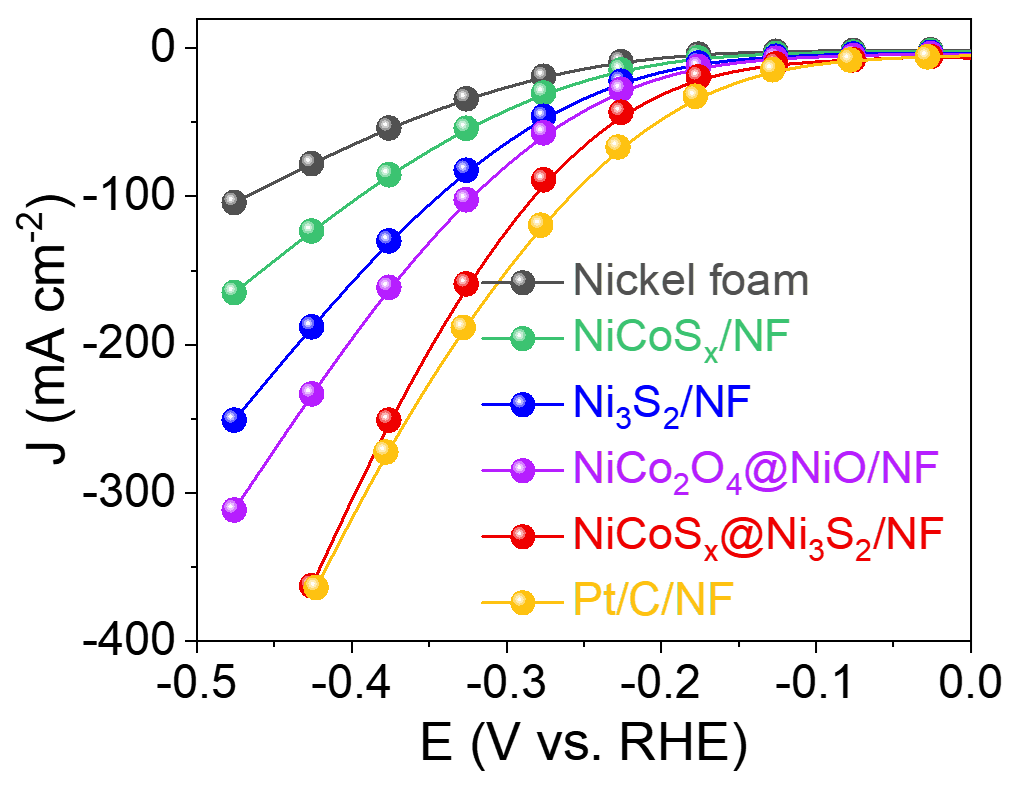


**Fig. S11** The steady-state polarization curves for HER by steady-state polarization method of nickel foam, NiCoS*_x_*/NF, Ni_3_S_2_/NF, NiCo_2_O_4_@NiO/NF, NiCoS*_x_*@Ni_3_S_2_/NF and Pt/C/NF in 1.0 M KOH solution


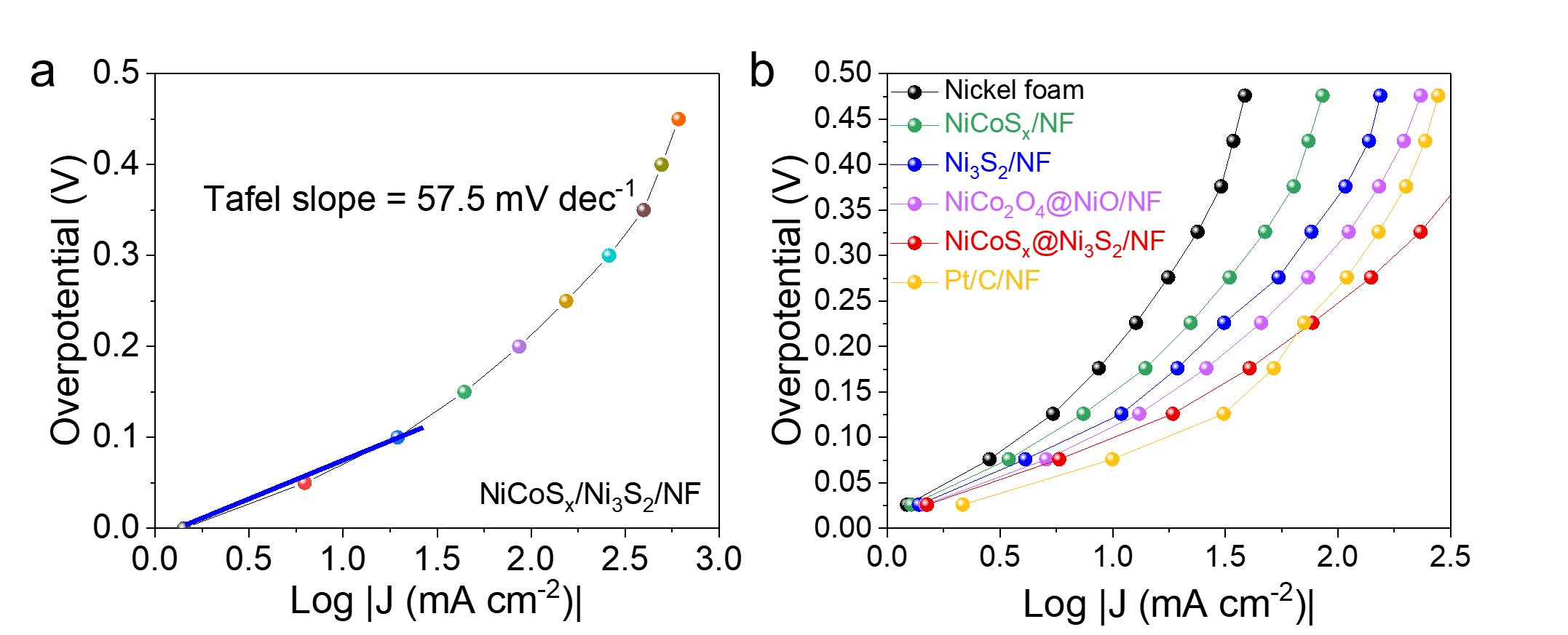


**Fig. S12** **a** The steady-state polarization Tafel curve of NiCoS*_x_*@Ni_3_S_2_/NF in 1.0 M KOH solution. **b** The steady-state polarization Tafel curve for nickel foam, NiCoS*_x_*/NF, Ni_3_S_2_/NF, NiCo_2_O_4_@NiO/NF, NiCoS*_x_*@Ni_3_S_2_/NF and Pt/C/NF in 1.0 M KOH solution


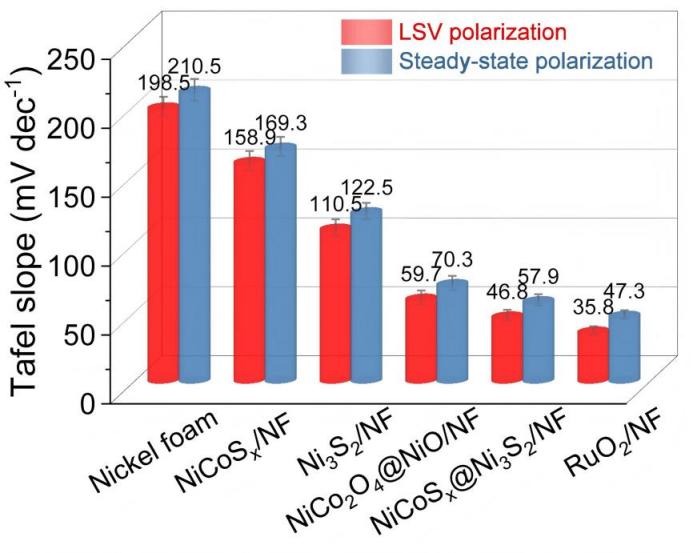


**Fig. S13** Contrast bar graphs of LSV-fitted Tafel slope and Tafel slope formulated by the steady-state polarization method for HER


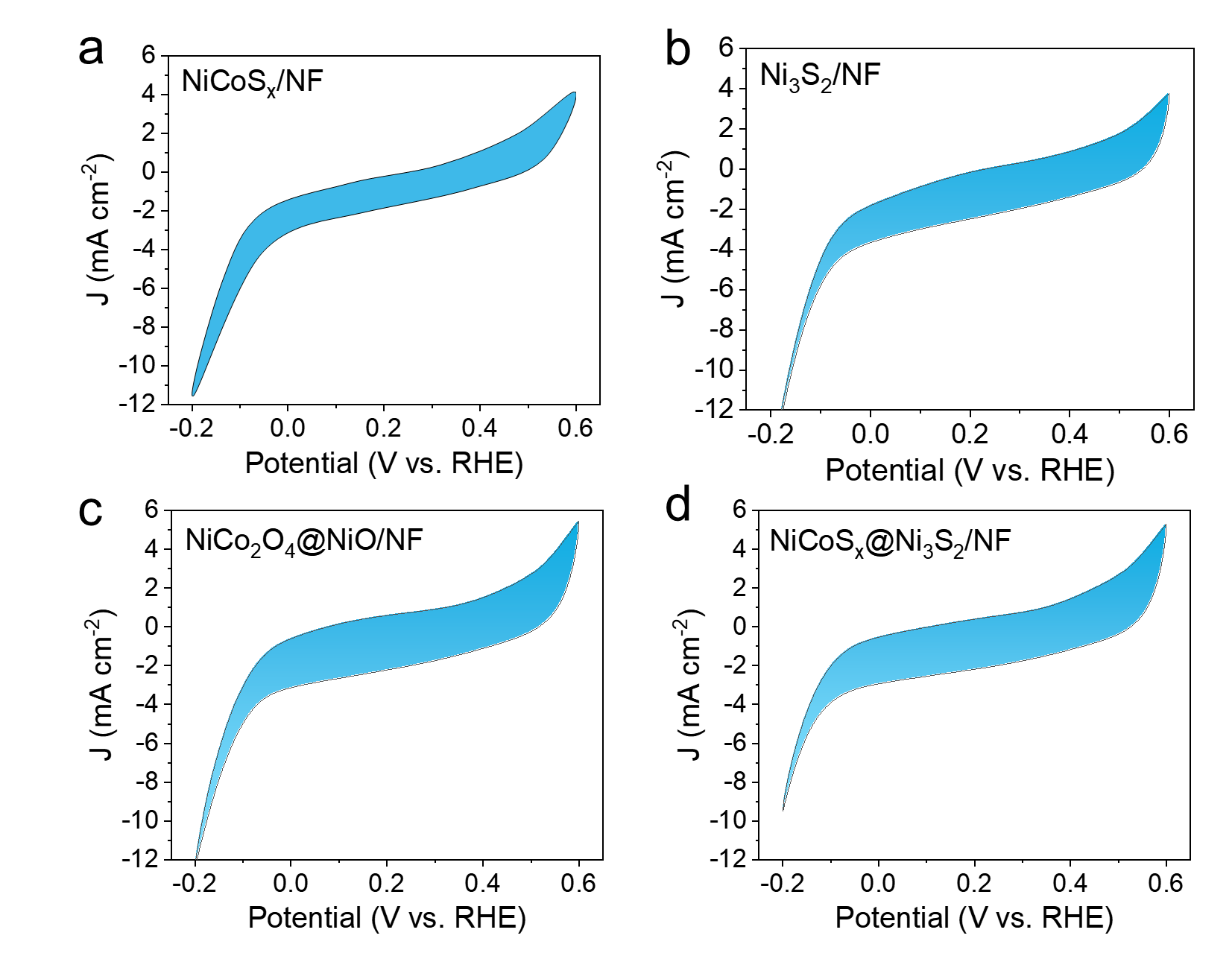
**Fig. S14** CVs of the catalysts with a scan rate of 50 mV s^−1^ in 1.0 M KOH solution with potential from −0.2 to 0.6 V (VS RHE) for HER: **a** NiCoS*_x_*/NF, **b** Ni_3_S_2_/NF, **c** NiCo_2_O_4_@NiO/NF, **d** NiCoS*_x_*@Ni_3_S_2_/NF


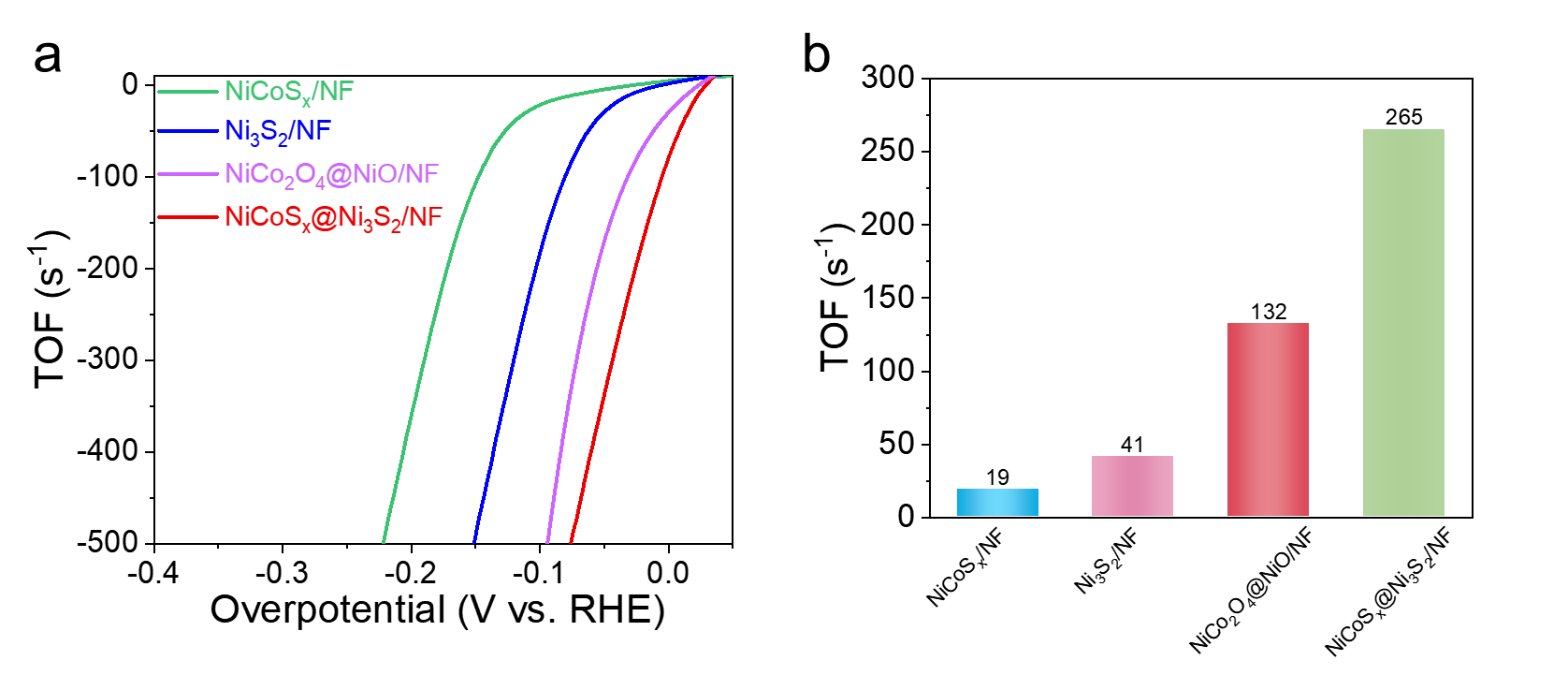
**Fig. S15** Contrast graphs of: **a** TOF values derived from LSV curves. **b** Calculated TOF values at 50 mV overpotential for NiCoS*_x_*/NF, Ni_3_S_2_/NF, NiCo_2_O_4_@NiO/NF, and NiCoS*_x_*@Ni_3_S_2_/NF under HER conditions

**
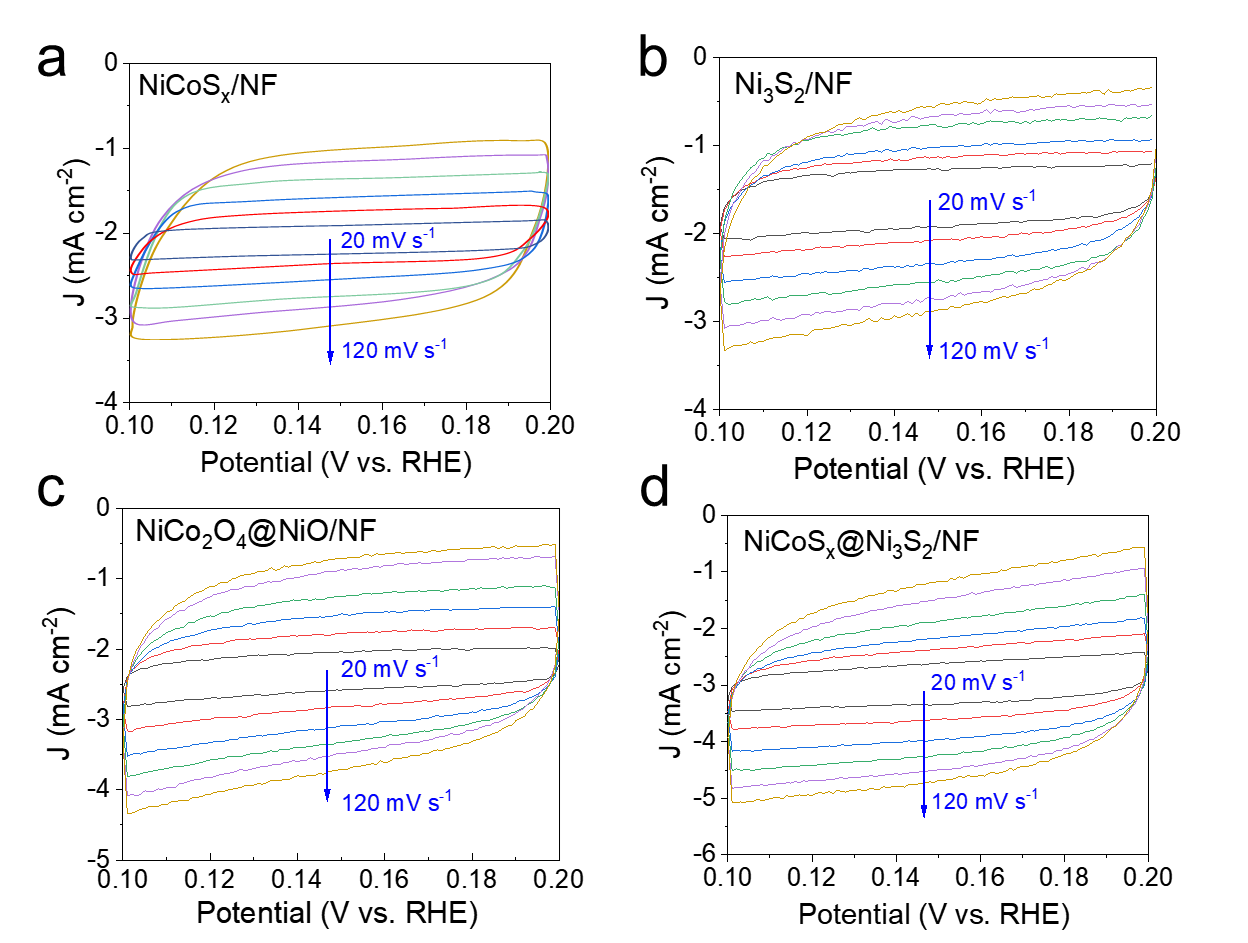
Fig. S16** The CV curves of HER in potential range of 0.1 to 0.2 V (vs. RHE): **a** NiCoS*_x_*/NF, **b** Ni_3_S_2_/NF, **c** NiCo_2_O_4_@NiO/NF, **d** NiCoS*_x_*@Ni_3_S_2_/NF


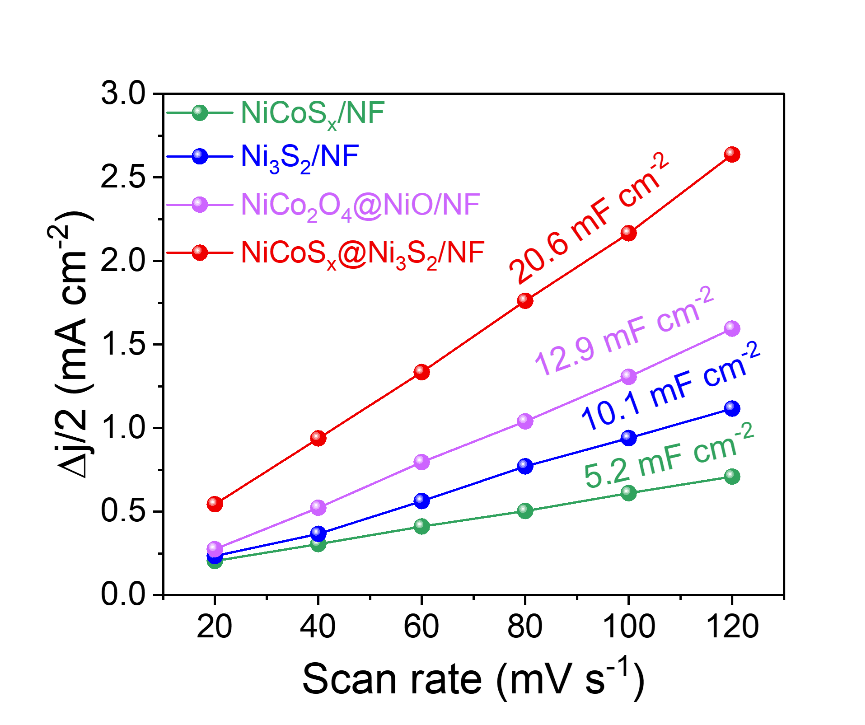


**Fig. S17** The C_dl_ measurements for HER with potential from 0.1 to 0.2 V (vs. RHE): NiCoS*_x_*/NF, Ni_3_S_2_/NF, NiCo_2_O_4_@NiO/NF, and NiCoS*_x_*@Ni_3_S_2_/NF


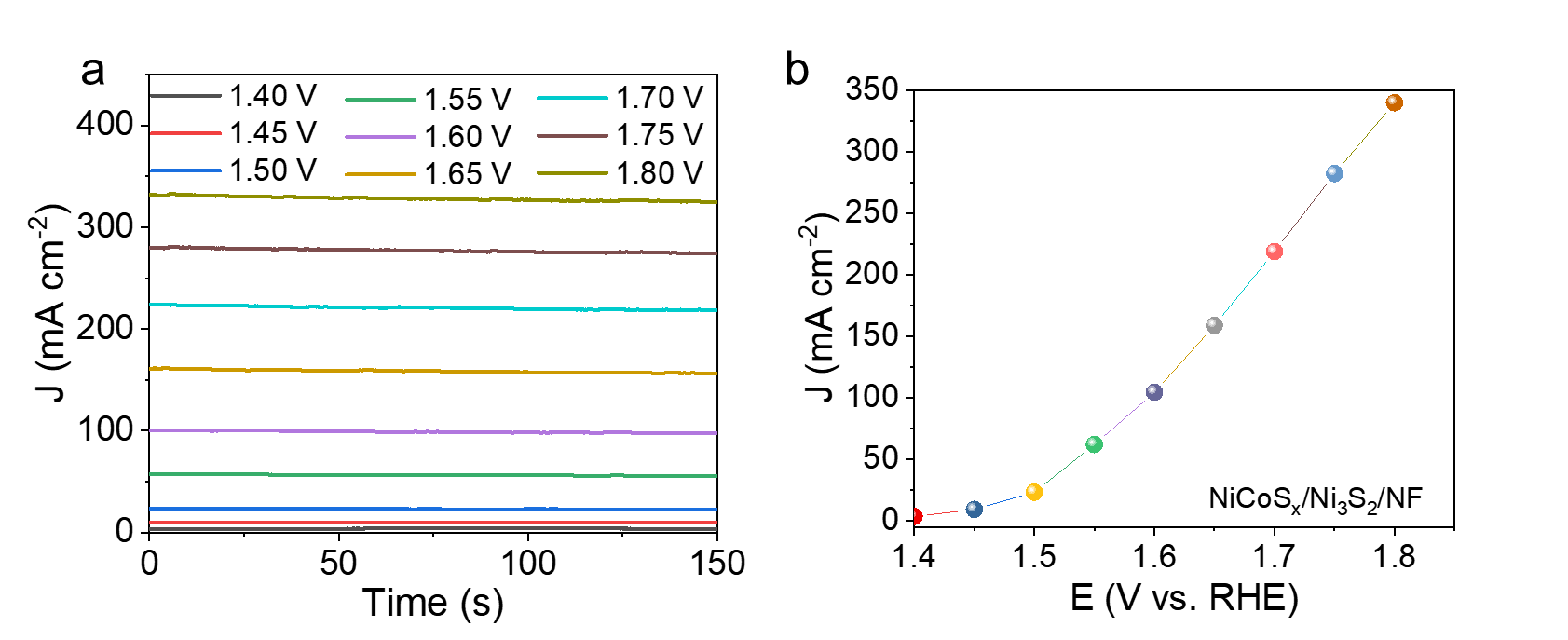


**Fig. S18 a** Chronoamperometric stability evaluation of NiCoS*_x_*@Ni_3_S_2_/NF for OER in 1.0 M KOH solution at applied potentials from 0 to −0.45 V vs. RHE. **b** The plot of sampled HER by the steady-state polarization method


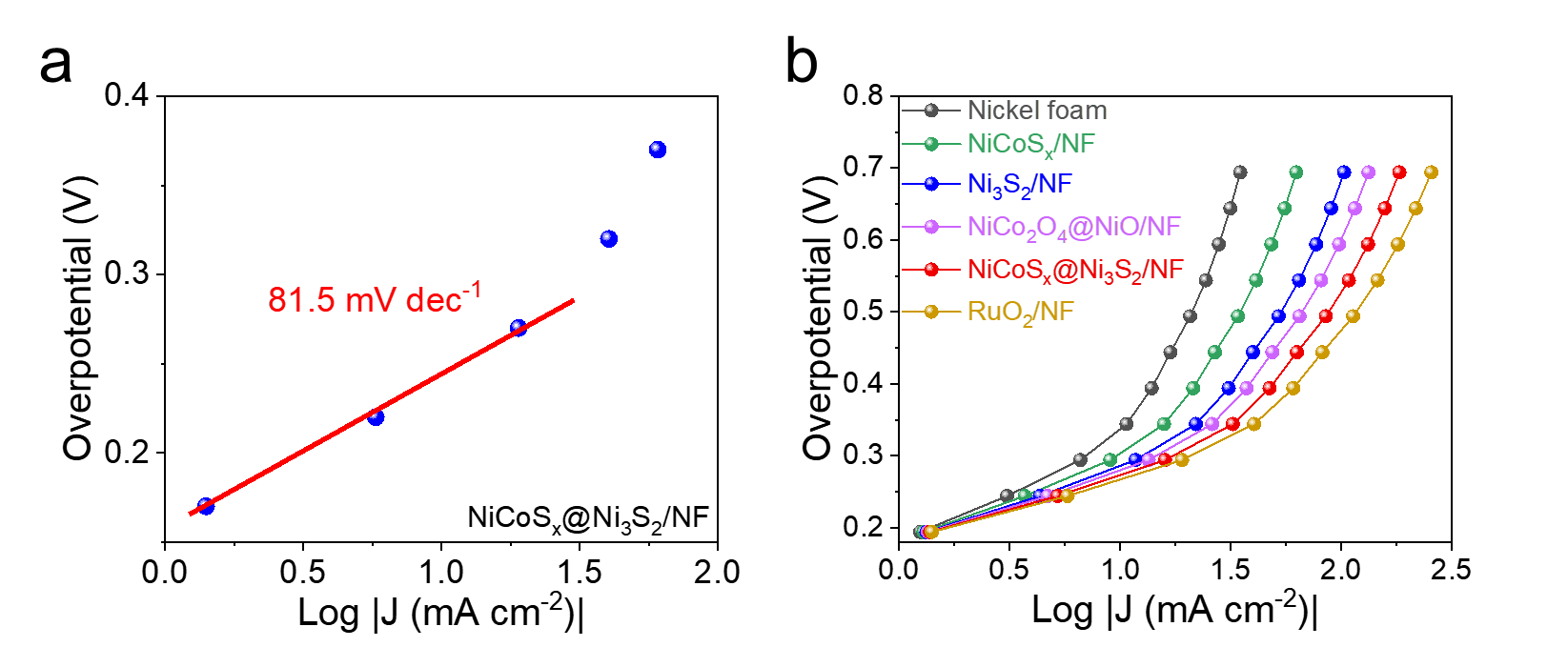


**Fig. S19 a** CA responses of NiCoS*_x_*@Ni_3_S_2_/NF for OER in 1.0 M KOH solution under stabilized catalytic conditions. **b** Steady-state polarization curves of OER activity in 1.0 M KOH solution


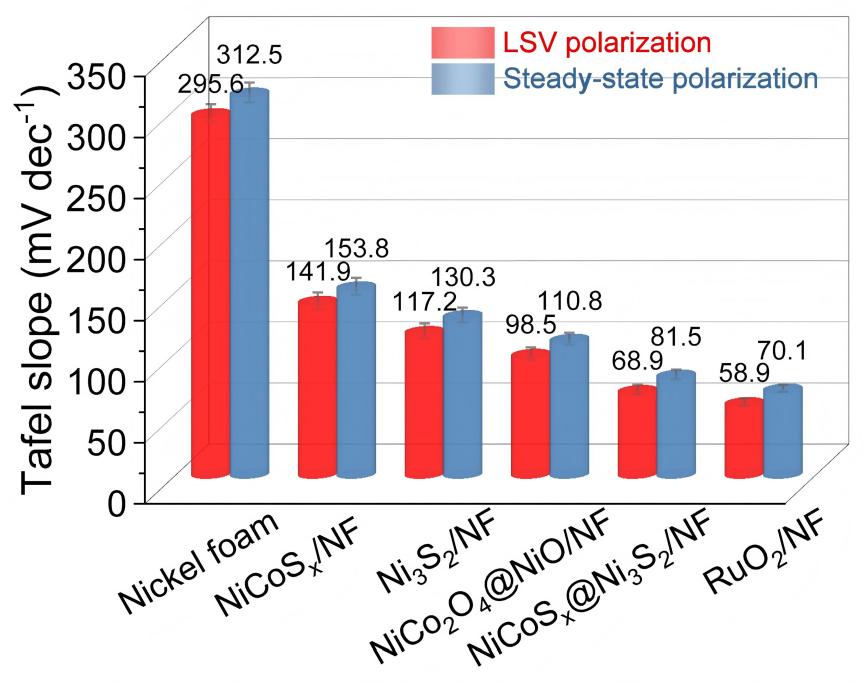


**Fig. S20** Contrast bar graphs of LSV-fitted Tafel slope and Tafel slope formulated by the steady-state polarization method for OER


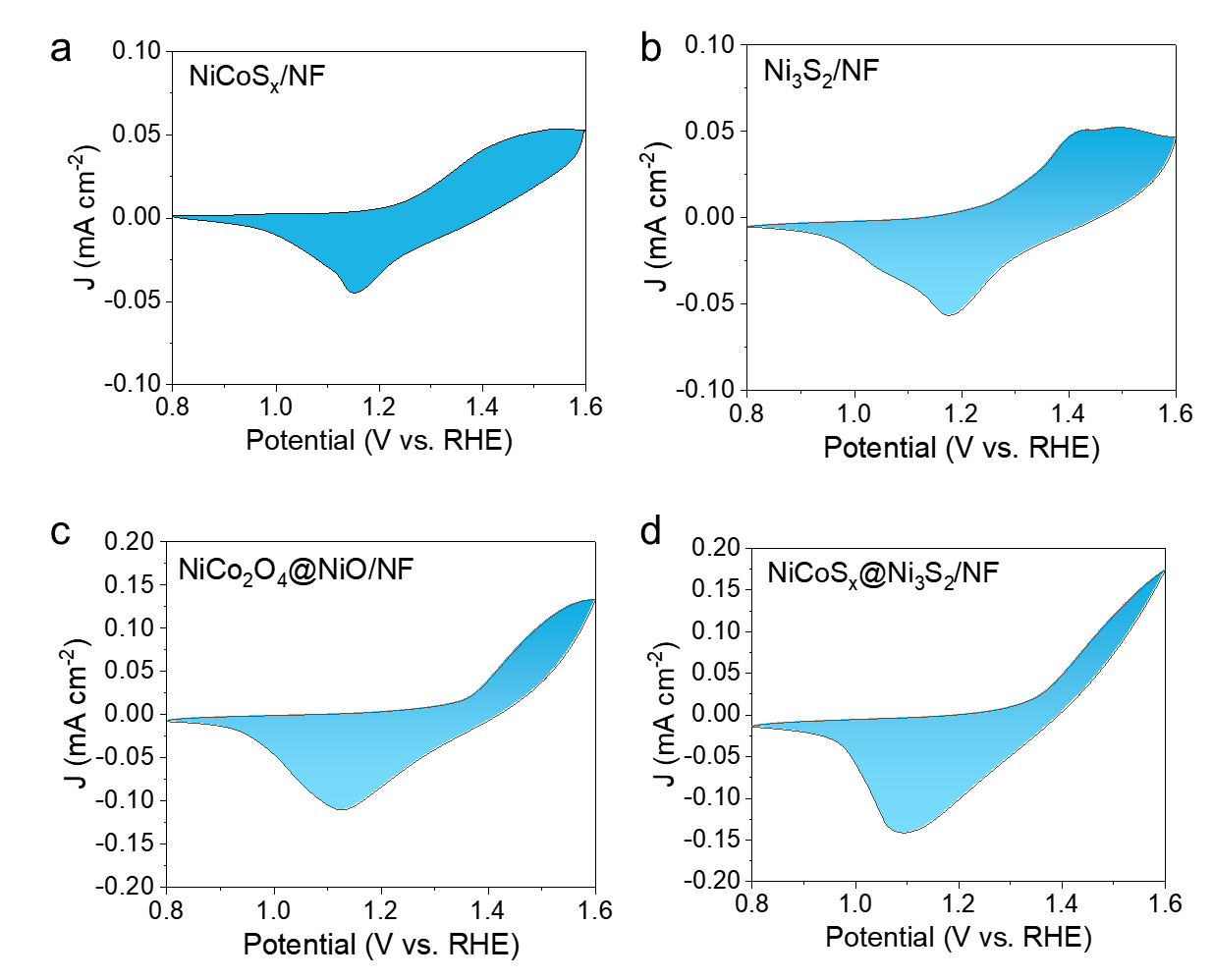
**Fig. S21** CV curves of catalytic electrodes at a scan rate of 50 mV s^−1^ in 1.0 M KOH solution (0.8~1.6 V vs. RHE) for OER: **a** NiCoS*_x_*/NF, **b** Ni_3_S_2_/NF, **c** NiCo_2_O_4_@NiO/NF, **d** NiCoS*_x_*@Ni_3_S_2_/NF for OER


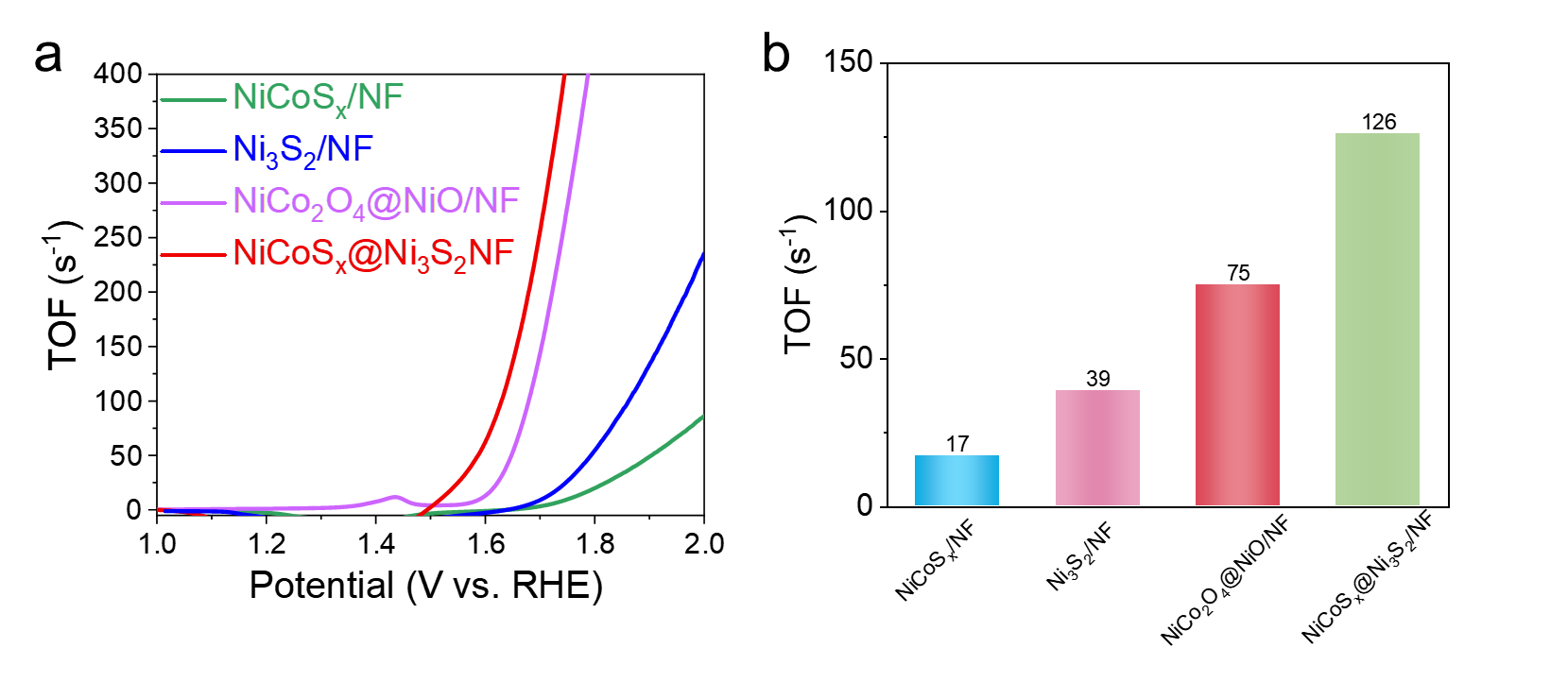


**Fig. S22** Comparison of: **a** TOF values derived from LSV analysis and **b** TOF values at 200 mV overpotential for NiCoS*_x_*/NF, Ni_3_S_2_/NF, NiCo_2_O_4_@NiO/NF and NiCoS*_x_*@Ni_3_S_2_/NF for OER


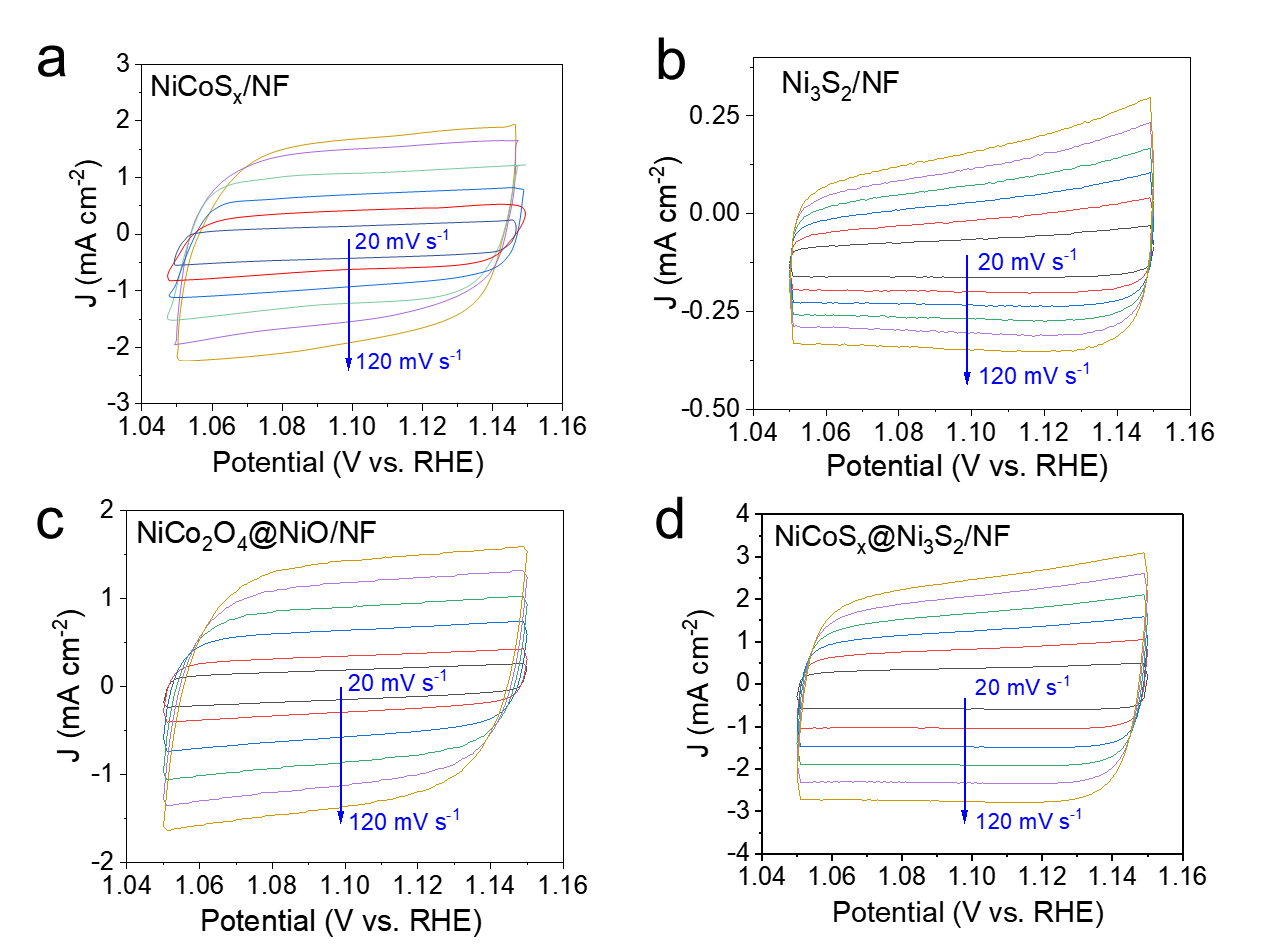
**Fig. S23** CV curves for OER at 1.05~1.15 V vs. RHE: **a** NiCoS*_x_*/NF, **b** Ni_3_S_2_/NF, **c** NiCo_2_O_4_@NiO/NF, **d** NiCoS*_x_*@Ni_3_S_2_/NF


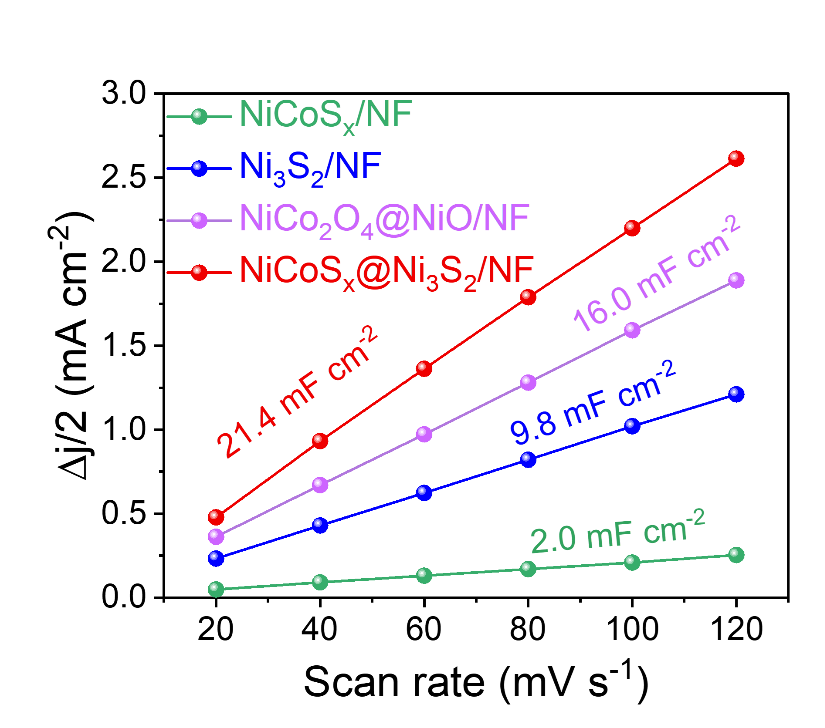


**Fig. S24** C_dl_ values of NiCoS*_x_*/NF, Ni_3_S_2_/NF, NiCo_2_O_4_@NiO/NF and NiCoS*_x_*@Ni_3_S_2_/NF for OER


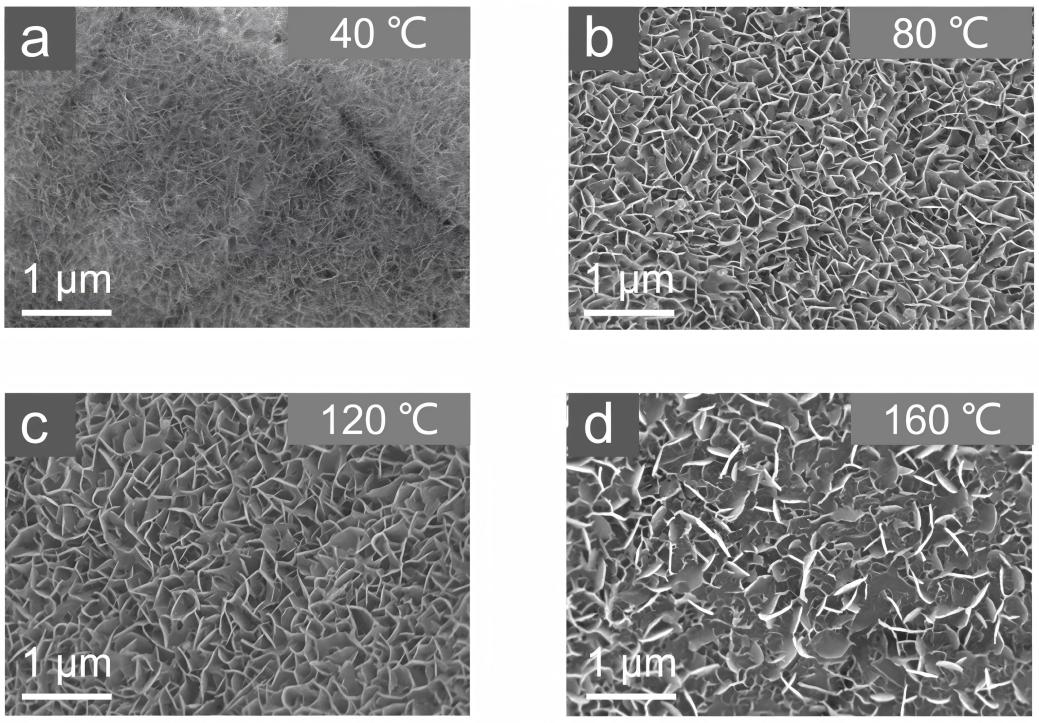


**Fig. S25** Morphological evolution of Ni(OH)_2_ catalysts at different cation exchange temperatures: **a** 40 °C, **b** 80 °C, **c** 120 °C, **d** 160 °C


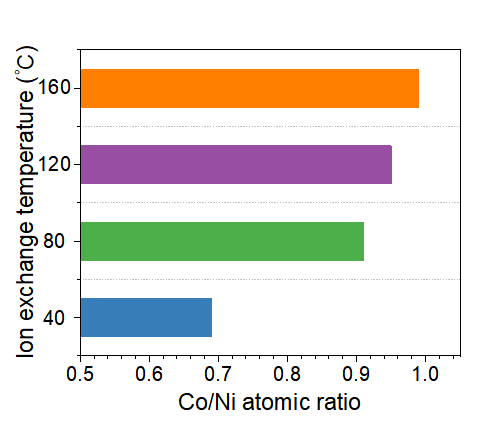


**Fig. S26** Co/Ni atomic ratio of catalysts as a function of Co^2+^ exchange temperature

**Fig. S27** EPR spectra of catalysts prepared at different Co^2+^ exchange temperatures

**Fig. S28** LSV curves of catalysts for **a** HER and **b** OER prepared at different Co^2+^ exchange temperatures

**
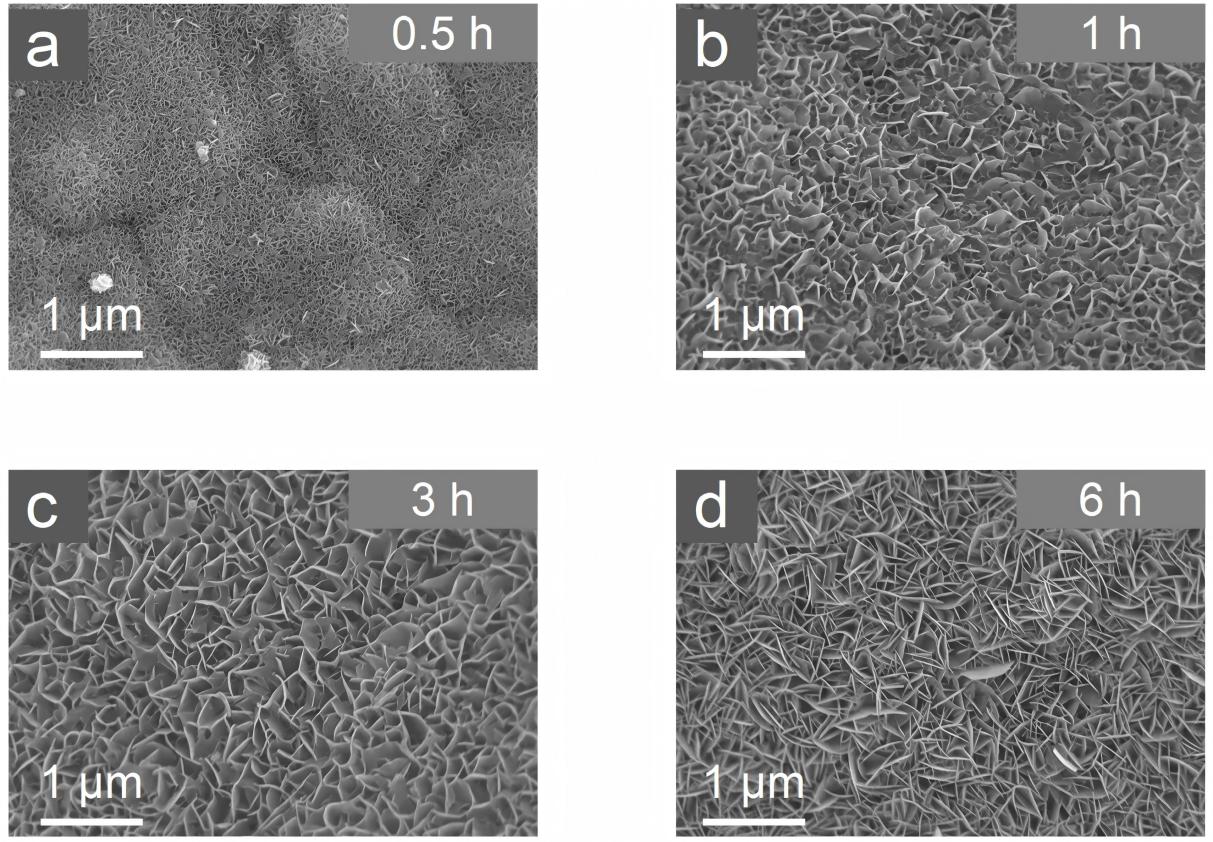
**

**Fig. S29** SEM images of catalysts prepared at different Co^2+^ exchange reaction times: **a** 0.5 h, **b** 1 h, **c** 3 h, **d** 6 h


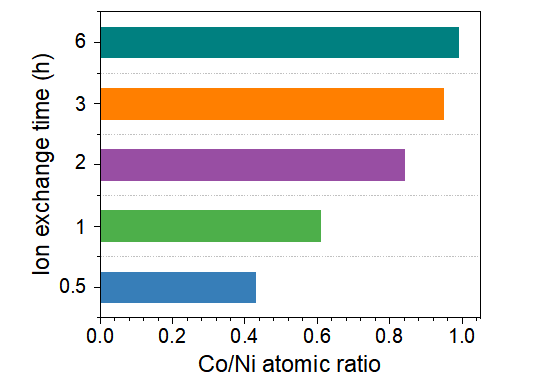


**Fig. S30** Co/Ni atomic ratio of catalysts at different Co^2+^ exchange reaction times (0.5 h, 1 h, 2 h, 3 h, 6 h)


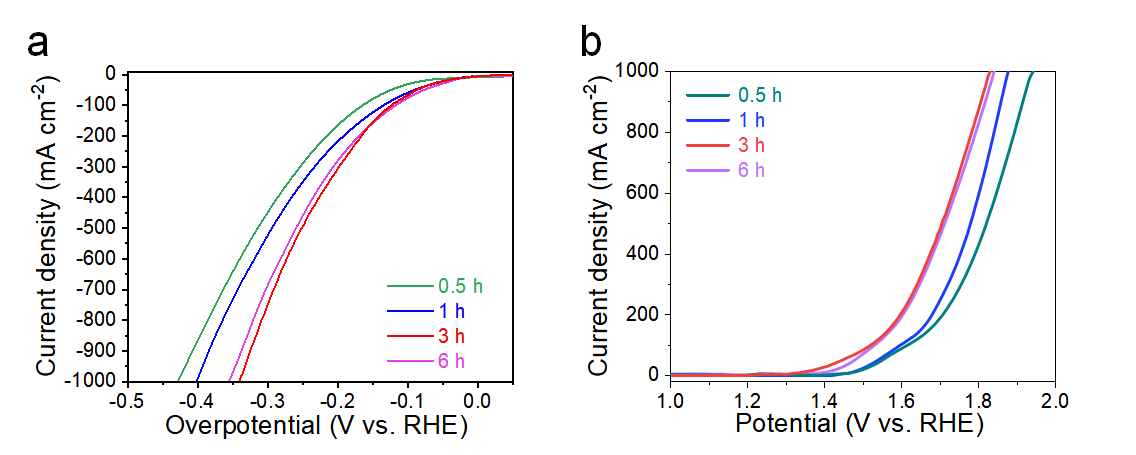


**Fig. S31** LSV curves of catalysts for **a** HER (a) and **b** OER prepared at different reaction times (0.5 h, 1 h, 3 h, 6 h)

**
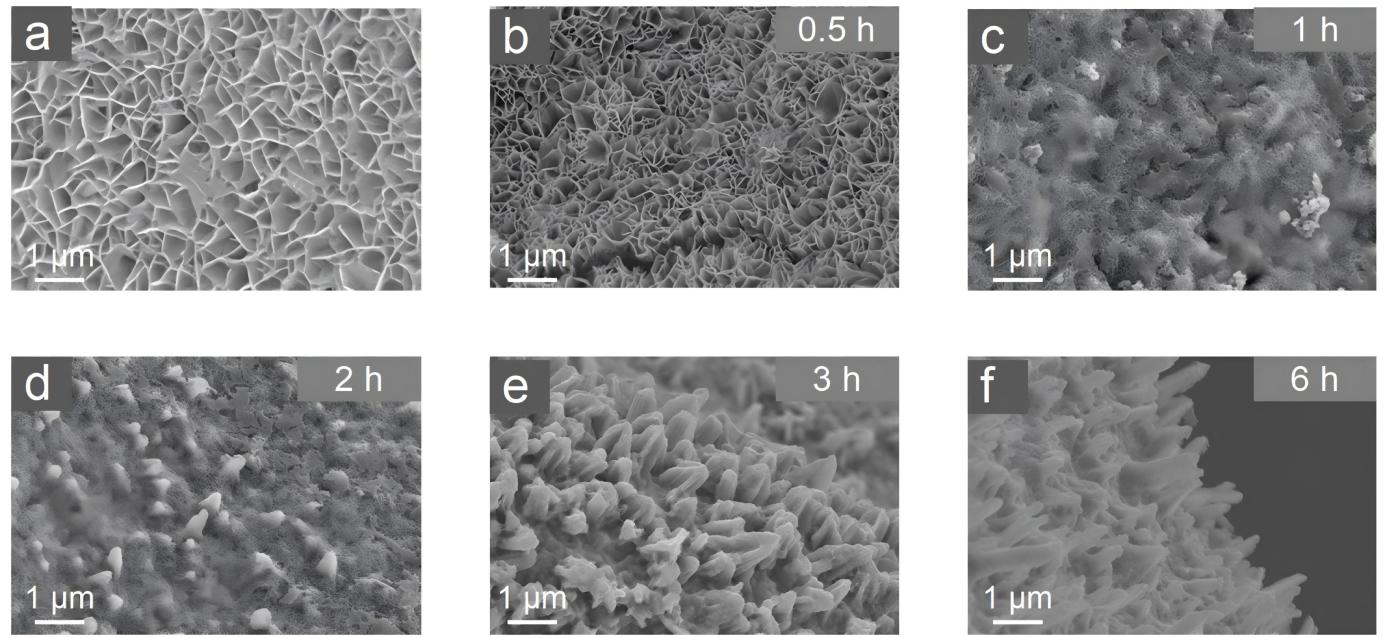
**

**Fig. S32** SEM images of catalysts prepared at different S ion exchange reaction times: **a** 0 h, **b** 0.5 h, **c** 1 h, **d** 2 h, **e** 3 h, **f** 6 h

**Fig. S33** XRD patterns of NiCoS*_x_*@Ni_3_S_2_/NF catalysts prepared at different reaction times

**Fig. S34** EPR spectra of NiCoS*ₓ*@Ni_3_S_2_/NF catalysts prepared at different reaction times


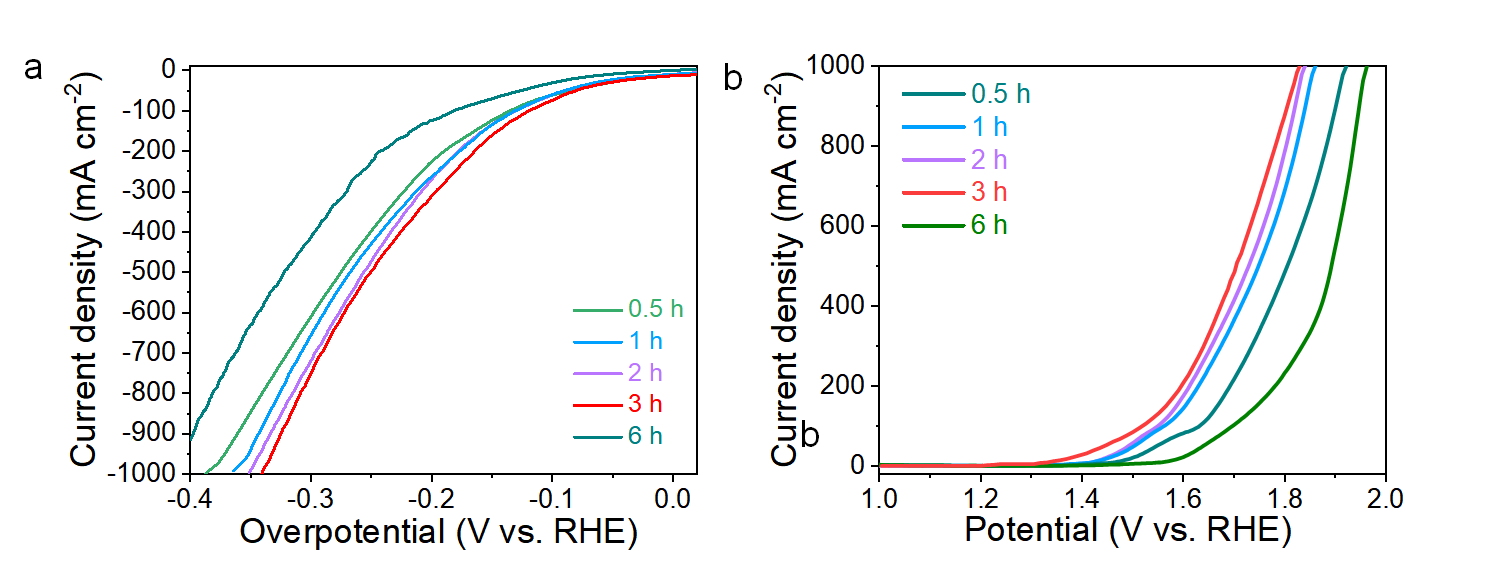


**Fig. S35** LSV curves of NiCoS*ₓ*@Ni_3_S_2_/NF catalysts for **a** HER and **b** OER prepared at different reaction times

**
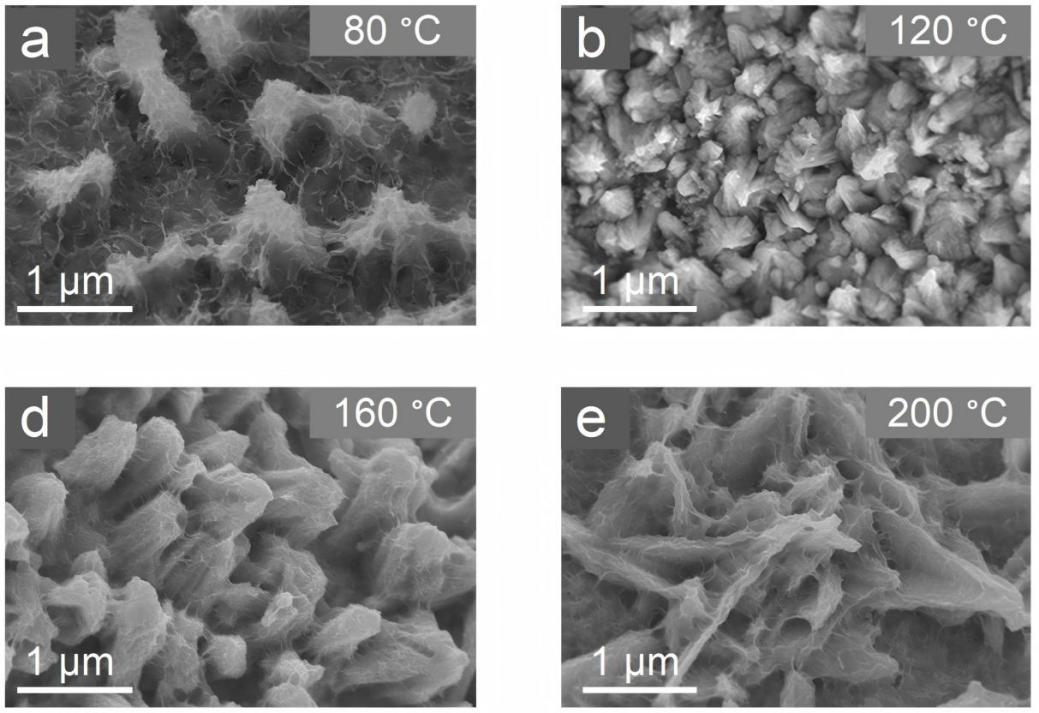
**

**Fig. S36** SEM images of catalysts prepared at different synthesis temperatures: **a** 80 °C, **b** 120 °C, **c** 160 °C, **d** 200 °C

**Fig. S37** EPR spectra of catalysts prepared at different synthesis temperatures (80 °C, 120 °C, 160 °C, 200 °C)


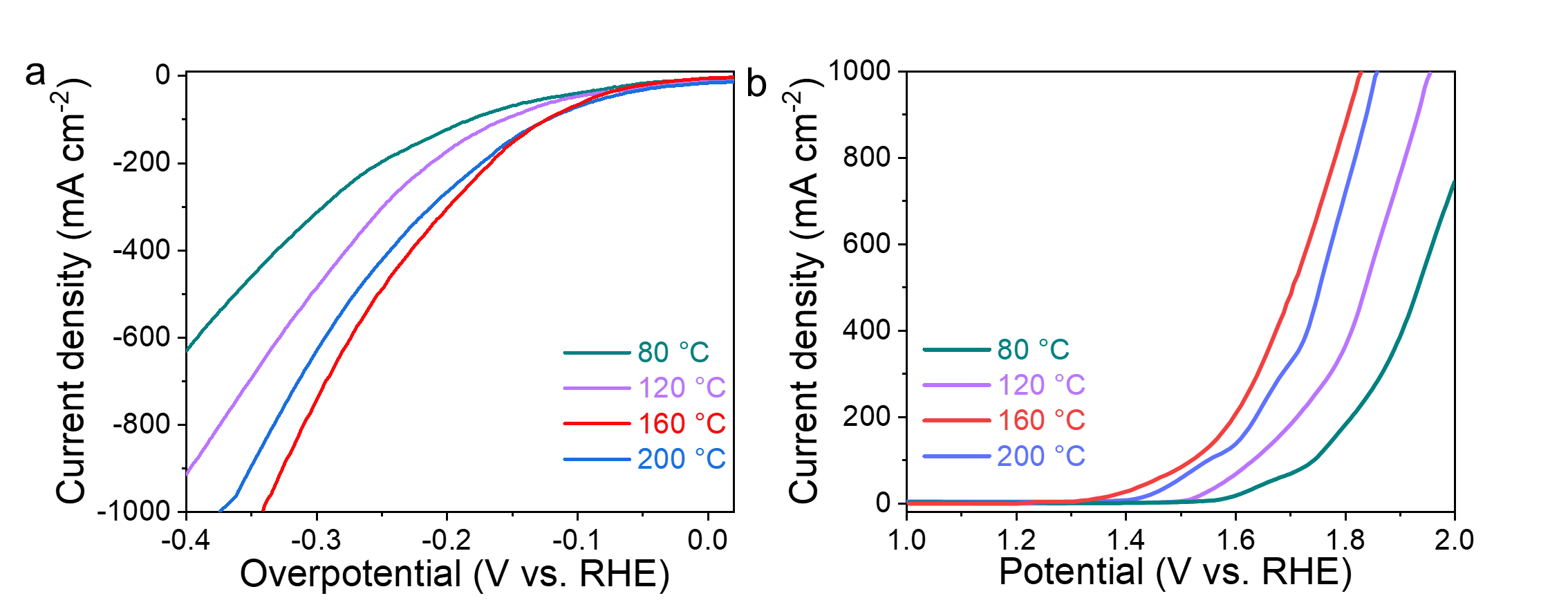


**Fig. S38** LSV curves of catalysts for **a** HER and **b** OER prepared at different synthesis temperatures


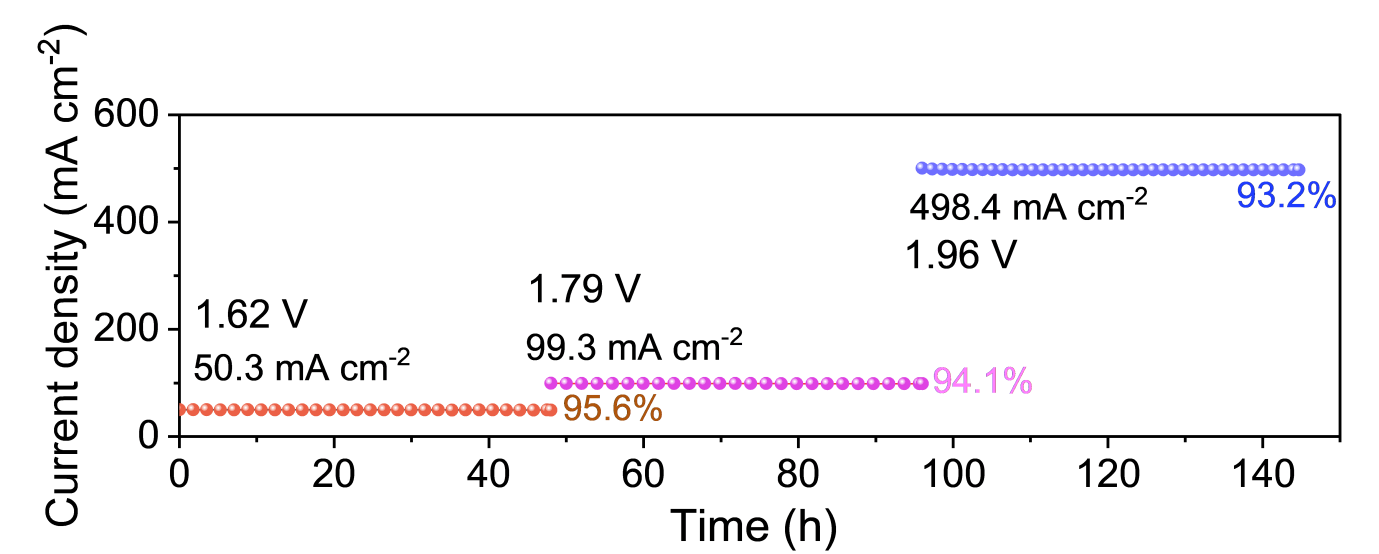


**Fig. S39** Long-term chronoamperometric stability curves of the electrolyzer under different constant potentials


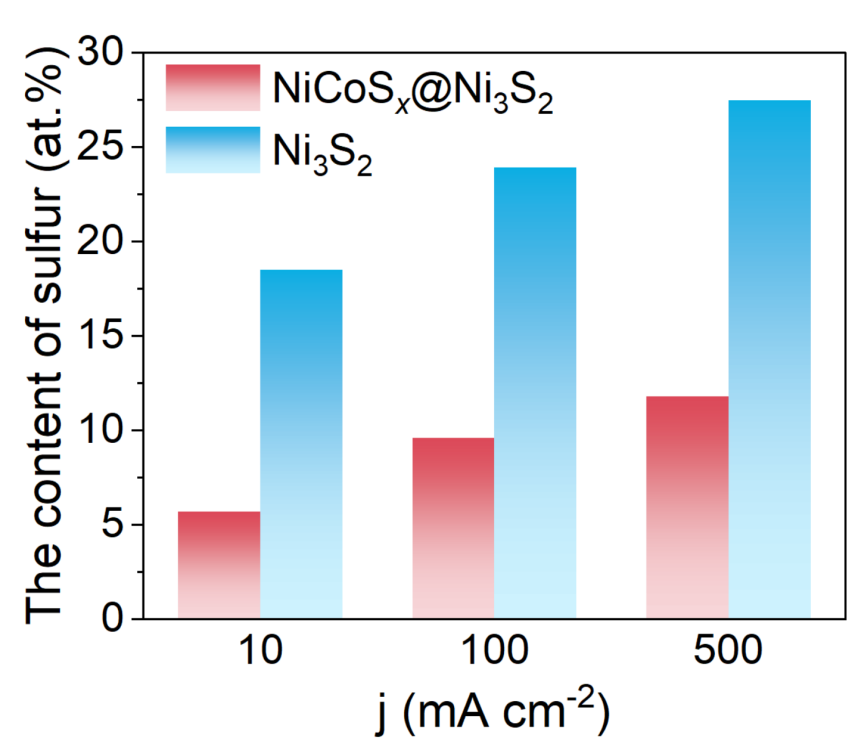


**Fig. S40** The content of sulfur under 10, 100 and 500 mA cm⁻^2^ after OER for 192 h
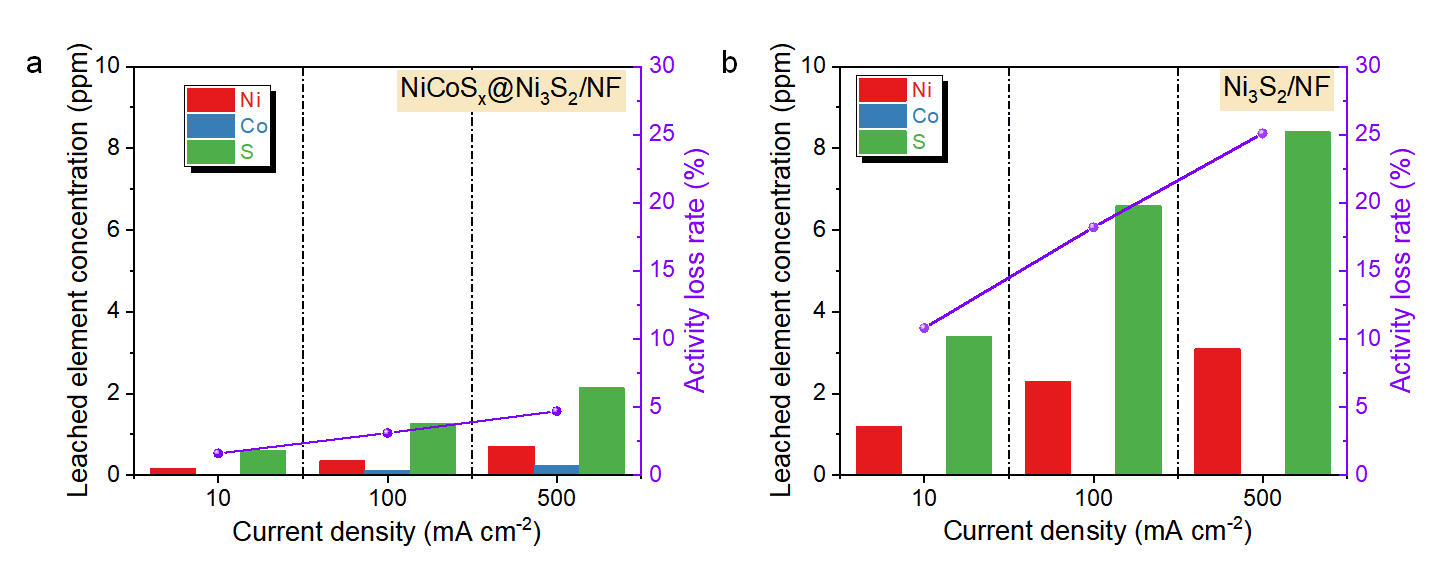


**Fig. S41** Stability comparison of NiCoS*_x_*@Ni_3_S_2_/NF and Ni_3_S_2_/NF after 192 h OER durability tests. Leached element concentrations (Ni: red, Co: blue, S: green) and activity loss rate (purple line) of **a** NiCoS*_x_*@Ni_3_S_2_/NF and **b** Ni_3_S_2_/NF at 10, 100, and 500 mA cm⁻^2^


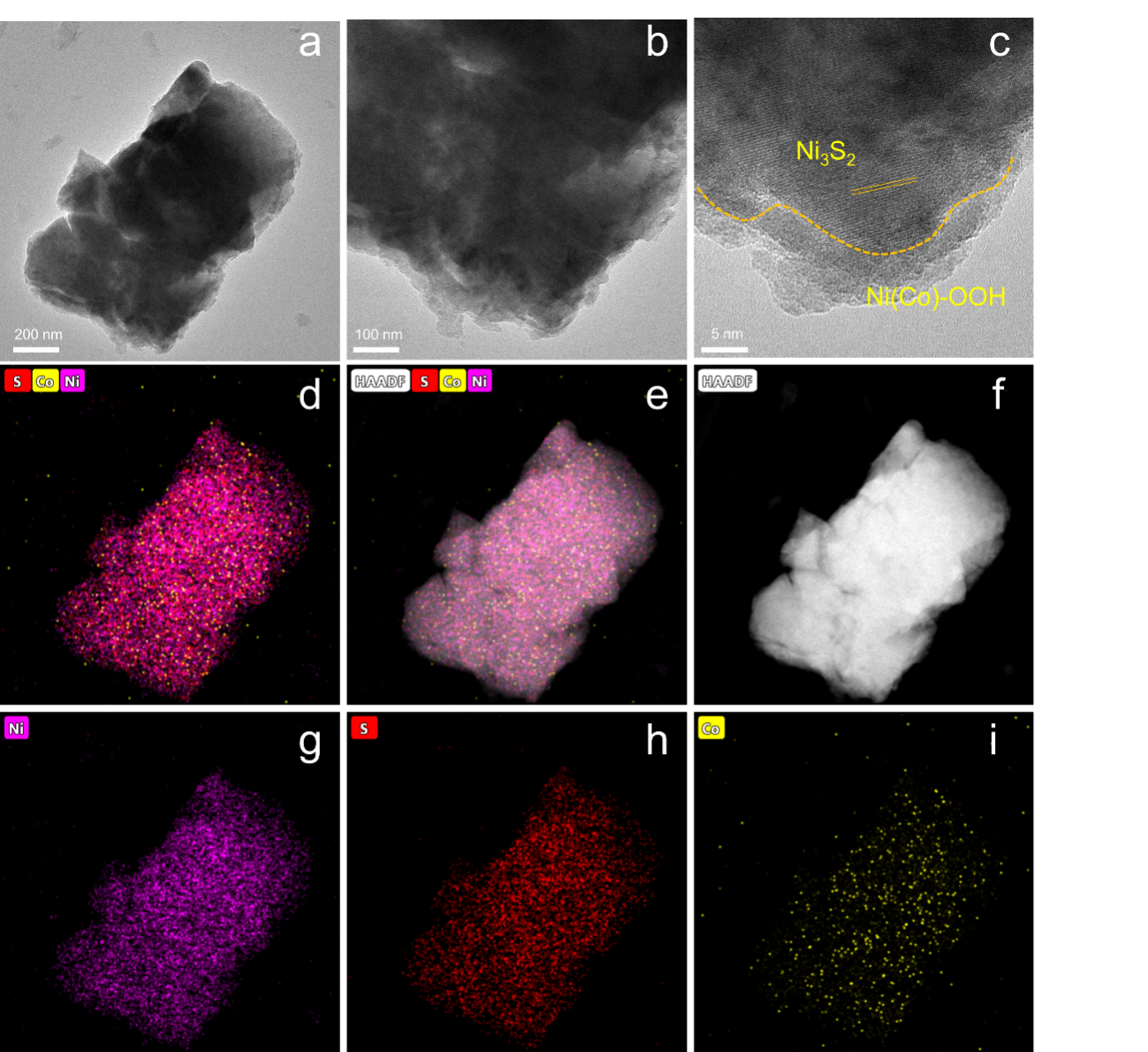
**Fig. S42** The NiCoS*_x_*@Ni_3_S_2_/NF morphology after OER reaction: **a**, **b** TEM images, **c** HRTEM image, **d**-**i** EDX elemental mappings


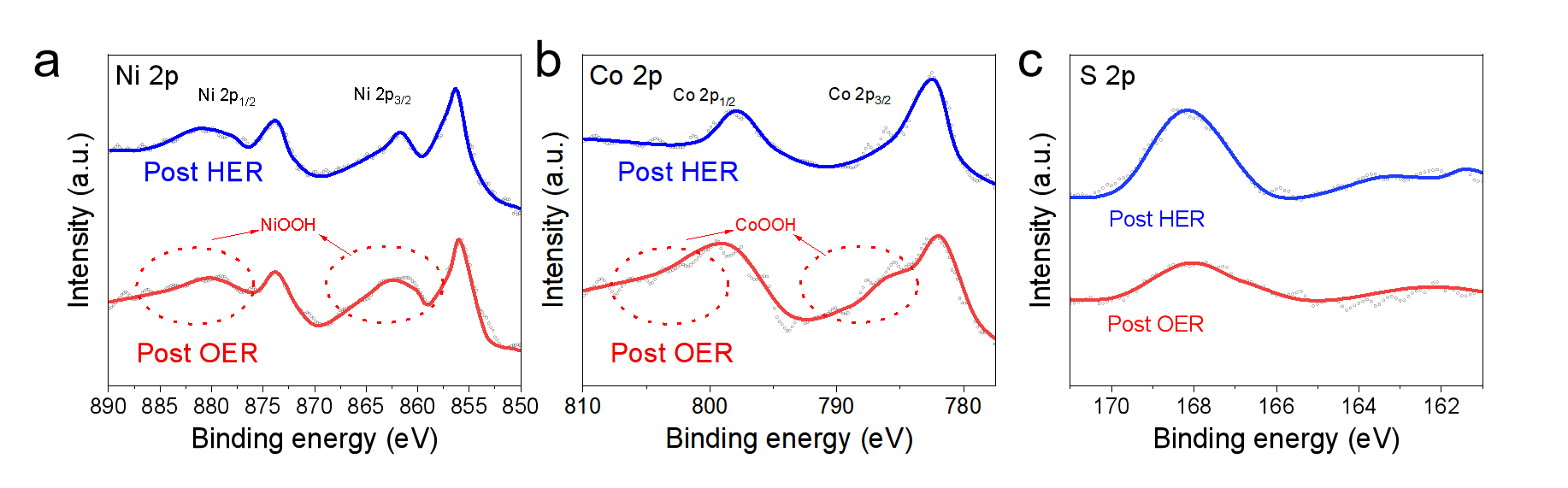


**Fig. S43 a** Ni 2p, **b** Co 2p, **c** S 2p XPS spectra after the HER and OER

**Fig. S44** Raman spectra of fresh and recovered catalysts after OER


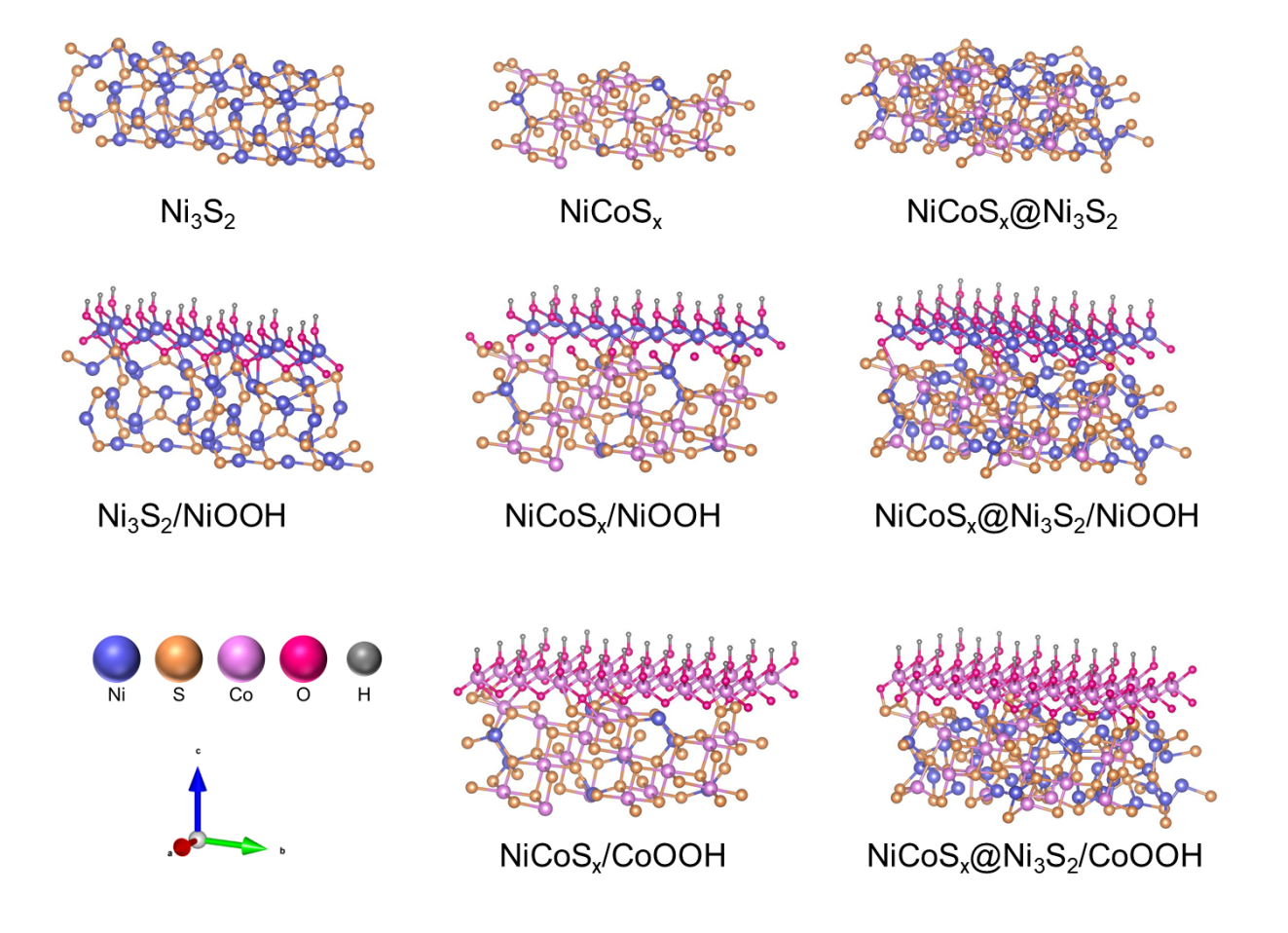


**Fig. S45** The theoretical models of optimization


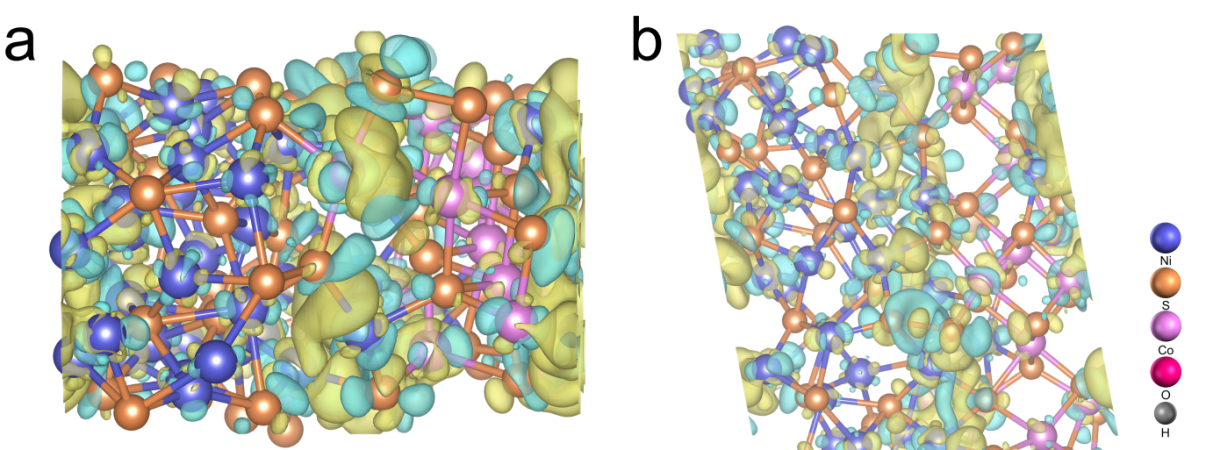


**Fig. S46** The mechanism of simulation calculation: **a**, **b** Charge density difference plot across the interface of NiCoS*_x_*@Ni_3_S_2_


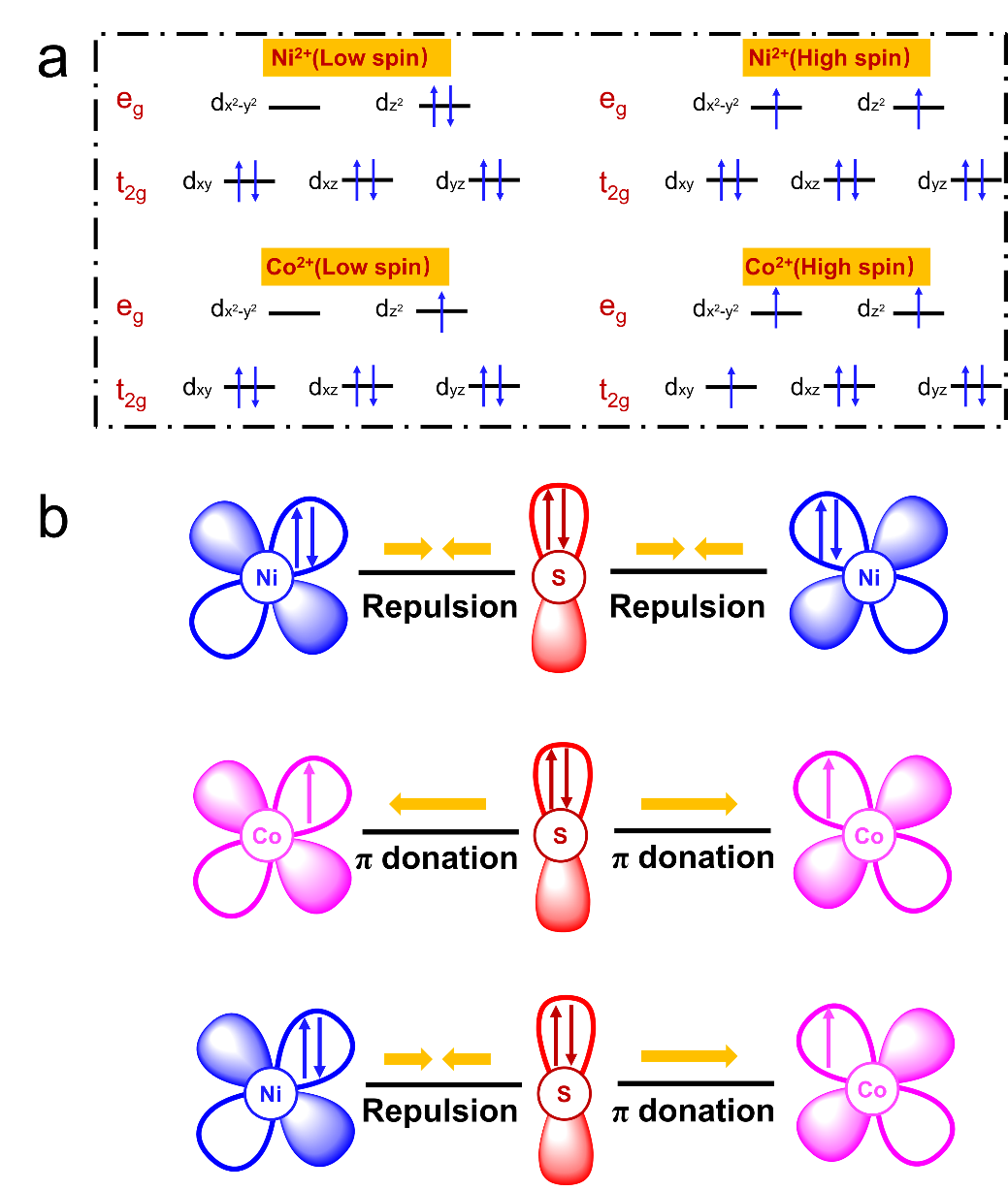


**Fig. S47 a** Electron configuration of the 3*d* orbital of Ni^2+^ and Co^2+^. **b** Mechanistic diagram of the electron coupling effect at the NiCoS*_x_*@Ni_3_S_2_ heterointerface


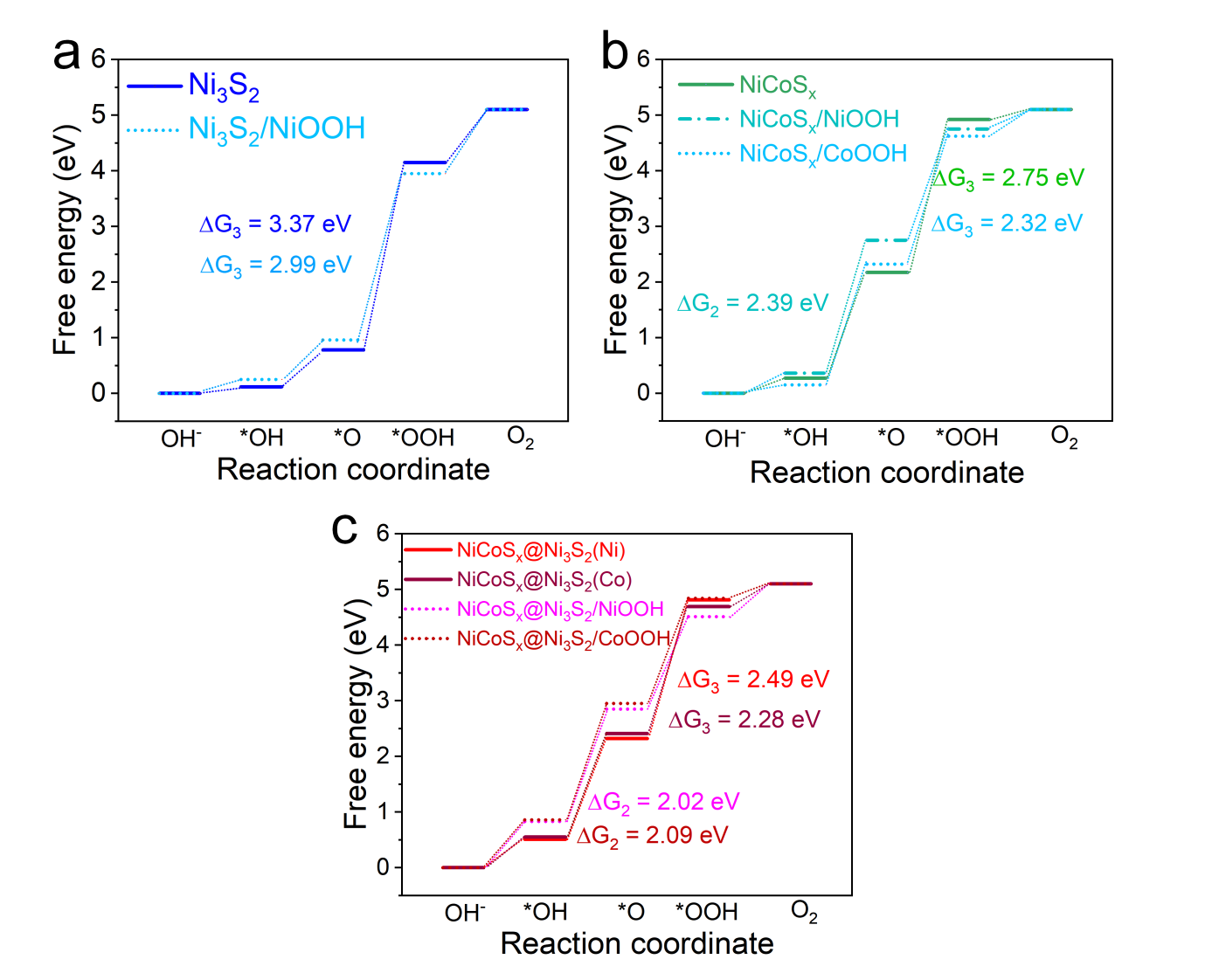


**Fig. S48 a-c** Calculated free-energy diagram of OER intermediates

**Table S1** The structure parameters of NiCoS*_x_*@Ni_3_S_2_ from the EXAFS fitting (S_0_^2^=0.80)

| **Sample** | **Path** | **CN** | ***R* (Å)** | ***σ*^2^ (×10**^−^**^3^Å^2^)** | **Δ*E*_0_ (eV)** | **R-factor (%)** |
| --- | --- | --- | --- | --- | --- | --- |
| NiCoS*_x_*@Ni_3_S_2_ | Ni-S | 2.7**±**0.7 | 2.05**±**0.03 | 5.3**±**0.7 | 1.70**±**0.57 | 0.012 |
|  | Co-S | 3.8**±**0.6 | 2.11**±**0.02 | 6.6±1.3 | 1.95**±**0.42 | 0.015 |

C.N.: coordination numbers;

R (Å): bond distance;

σ^2^ (10^−3^ Å^2^): Debye-Waller factors;

ΔE_0_ (eV): the inner potential correction;

R factor: goodness of fit.

**Table S2** Comparison of HER performance in 1.0 M KOH solution with other electrocatalysts

| Catalyst | Overpotential (mV) @10mA cm^−2^ | Refs. |
| --- | --- | --- |
| **NiCoS*_x_*@Ni_3_S_2_/NF** | **44** | **This work** |
| Ni_3_S_2_/NF | 78 | [S1] |
| 2H Nb_1.35_S_2_ | 140 | [S2] |
| CoP/FeCoP_x_ | 94 | [S3] |
| CoMoS_x_ | 120 | [S4] |
| Cu NDs/Ni_3_S_2_ NTs-CFs | 76.2 | [S5] |
| S‐NiFe_2_O_4_ | 80 | [S6] |
| Co_3_O_4_/MoS_2_ | 98 | [S7] |
| Ni-Fe-S | 108 | [S8] |
| Co_3_S_4_@FNC‐Co | 103 | [S9] |
| V-NiS/NiS_2_ | 94 | [S10] |
| MoS_2_/Ni_3_S_2_ | 89 | [S11] |
| NC-CoNi_2_S_4_@ReS_2_/CC | 87 | [S12] |

**Table S3** Comparison of OER performance in 1.0 M KOH solution with other electrocatalysts

| Catalyst | Overpotential (mV) @10mA cm^−2^ | Refs. |
| --- | --- | --- |
| NiCoS*_x_*@Ni_3_S_2_/NF | **212** | **This work** |
| Ni_3_S_2_/NF | 260 | [S1] |
| NC-CoNi_2_S_4_@ReS_2_/CC | 253 | [S12] |
| S-(Ni,Fe)OOH | 229 | [S13] |
| CoOOH/Co_9_S_8_ | 240 | [S14] |
| CoOOH/CoS_α_ | 220 | [S15] |
| CeO_2_-CoS_1.97_ | 264 | [S16] |
| NiO/NiS_2_ | 270 | [S17] |
| Fe-Co_9_S_8_@SNC | 273 | [S18] |
| MoS_2_‐NiS_2_ | 320 | [S19] |
| Co_3_S_4_@FNC‐Co | 250 | [S20] |
| Meso-Co-MoS_2_ | 268 | [S21] |
| MnCo_2_O_4.5_@NiS | 288 | [S22] |
| VMoS/MoNi | 223 | [S23] |

**Table S4** Comparison of water splitting performance in 1.0 M KOH solution with other electrocatalysts

| Catalyst | Voltage (V) @10 mA cm^−2^ | Refs. |
| --- | --- | --- |
| **NiCoS*_x_*@Ni_3_S_2_/NF** | **1.54** | **This work** |
| NC-CoNi_2_S_4_@ReS_2_/CC | 1.57 | [S12] |
| MoS_2_/NiS_2_\|\|MoS_2_/NiS_2_ | 1.59 | [S19] |
| VMoS/MoNi | 1.56 | [S23] |
| NiS\|\|NiS_2_ | 1.58 | [S24] |
| NiS\|\|Ni_2_P_2_S_6_ | 1.64 | [S25] |
| MoS_2_/NiS\|\|MoS_2_/NiS | 1.61 | [S26] |
| CoS_2_‐MoS_2_\|\|CoS_2_‐MoS_2_ | 1.6 | [S27] |
| Co_9_S_8_/Ni_3_S_2_\|\|Co_9_S_8_/Ni_3_S | 1.64 | [S28] |
| NiCoS*_x_*\|\|NiCoS*_x_* | 1.6 | [S29] |
| Ni‐Mo‐S\|\|Ni‐Mo‐S | 2.0 | [S30] |
| Co‐Ni_3_S_2_\|\|Co‐Ni_3_S_2_ | 1.54 | [S31] |
| CuCo_2_S_4_\|\|CuCo_2_S_4_ | 1.58 | [S32] |
| S-FeNi/NF\|\|S-FeNi/NF | 1.53 | [S33] |

**Table S5** The S content of ICP results under 10 mA cm^−2^ for 192 h

| Sample | S before OER (%) | S after OER (%) | Relative decrease (%) |
| --- | --- | --- | --- |
| NiCoS*_x_*@Ni_3_S_2_ | 31.4 | 29.6 | 5.7 |
| Ni_3_S_2_ | 39.4 | 32.1 | 18.5 |

**Table S6** The S content of ICP results under 100 mA cm^−2^ for 192 h

| Sample | S before OER (%) | S after OER (%) | Relative decrease (%) |
| --- | --- | --- | --- |
| NiCoS*_x_*@Ni_3_S_2_ | 31.4 | 28.4 | 9.6 |
| Ni_3_S_2_ | 39.4 | 30.0 | 23.9 |

**Table S7** The S content of ICP results under 500 mA cm^−2^ for 192 h

| Sample | S before OER (%) | S after OER (at.%) | Relative decrease (%) |
| --- | --- | --- | --- |
| NiCoS*_x_*@Ni_3_S_2_ | 31.4 | 27.7 | 11.8 |
| Ni_3_S_2_ | 39.4 | 22.7 | 27.5 |

**Table S8** Comparative stability of sulfide electrocatalysts for water splitting in 1.0 M KOH

| Catalyst | Electrolyte | Current density & durability | Ref. |
| --- | --- | --- | --- |
| **NiCoS*_x_*@Ni_3_S_2_/NF** | **1.0 M KOH** | **1000 mA cm⁻^2^ for 192h** | **This work** |
| Co_9_S_8_-NiCo_2_S_4_/N-rGO | 1.0 M KOH | 15 h OWS at 10 & 100 mA cm⁻^2^ | [S34] |
| NiS/NiCo_2_S_4_ heterostructure | Alkaline seawater | ≤100 mA cm⁻^2^ for long-term | [S35] |
| Fe-Ni_3_S_2_/MoO*_x_* | 1.0 M KOH | Mid-high current OWS | [S36] |
| Ni_3_S_2_/Ni(OH)_2_ | 1.0 M KOH | long-term OWS stability at 60 °C | [S37] |
| NiCo_2_S_4_@Ce-NiFe LDH/CeO_2_ nanoarrays | 1.0 M KOH | 100 h durability | [S38] |
| Dual-phase Ni-sulfide | 1.0 M KOH | 10 mA cm⁻^2^; (durability discussed) | [S39] |
| ReS_2_/Ni_3_S_2_ | 1.0 M KOH | ≥50 h; OWS enabled | [S40] |
| CeO_2_/NiCo_2_S_4_ | 1.0 M KOH | >200 h @ 500 mA cm^−2^ | [S41] |
| Mo-doped Ni_3_S_2_ | 1.0 M KOH | **120 mA cm⁻^2^** OER with **~90% retention** | [S42] |
| Ni_3_S_2_@Ta-NiFe-LDH | 1.0 M KOH | **100 h** stability reported at **50-100 mA** cm^−2^ | [S43] |
| Ni_3_S_2_@V-NiFe-LDH | 1.0 M KOH | **100 h** at **50 mA** cm^−2^ | [S44] |

**Supplementary References**

1. L.L. Feng, G.T. Yu, Y.Y. Wu, G.D. Li, H. Li et al., High-index faceted Ni_3_S_2_ nanosheet arrays as highly active and ultrastable electrocatalysts for water splitting. J. Am. Chem. Soc. **137**(43), 14023-14026 (2015). https://doi.org/10.1021/jacs.5b08186
2. J. Yang, A.R. Mohmad, Y. Wang, R. Fullon, X.J. Song et al., Ultrahigh-current-density niobium disulfide catalysts for hydrogen evolution. Nat. Mater. **18**(12), 1309-1314 (2019). https://doi.org/10.1038/s41563-019-0463-8
3. S. Zhang, C. Tan, R. Yan, X. Zou, F.L. Hu et al., Constructing built-in electric field in heterogeneous nanowire arrays for efficient overall water electrolysis. Angew. Chem. Int. Ed. **62**(26), e202302795 (2023). https://doi.org/10.1002/anie.202302795
4. X.Y. Shan, J. Liu, H.R. Mu, Y. Xiao, B.B. Mei et al., An engineered superhydrophilic/superaerophobic electrocatalyst composed of the supported CoMoS*_x_* chalcogel for overall water splitting. Angew. Chem. Int. Ed. **59**(4), 1659-1665 (2020). https://doi.org/10.1002/anie.201911617
5. J.X. Feng, J.Q. Wu, Y.X. Tong, G.R. Li, Efficient hydrogen evolution on Cu nanodots-decorated Ni_3_S_2_ nanotubes by optimizing atomic hydrogen adsorption and desorption. J. Am. Chem. Soc. **140**(2), 610-617 (2018). https://doi.org/10.1021/jacs.7b08521
6. J. Jin, J. Yin, H.B. Liu, B.L. Huang, Y. Hu et al., Atomic sulfur filling oxygen vacancies optimizes H absorption and boosts the hydrogen evolution reaction in alkaline media. Angew. Chem. Int. Ed. **60**, e202104055 (2021). https://doi.org/10.1002/anie.202104055
7. A. Muthurasu, V. Maruthapandian, H.Y. Kim, Metal-organic framework derived Co_3_O_4_/MoS_2_ heterostructure for efficient bifunctional electrocatalysts for oxygen evolution reaction and hydrogen evolution reaction. Appl. Catal. B **248**, 202-210 (2019). https://doi.org/10.1016/j.apcatb.2019.02.014
8. T.V.T. Mai, L.K. Huynh, Atmospheric chemistry of oxazole: the mechanism and kinetic studies of the oxidation reaction initiated by OH radicals. New J. Chem. **45**(4), 2237-2248 (2021). https://doi.org/10.1039/D0NJ05797E
9. X. Zhu, J. Dai, L. Li, D. Zhao, Z. Wu et al., Hierarchical carbon microflowers supported defect-rich Co_3_S_4_ nanoparticles: an efficient electrocatalyst for water splitting. Carbon **160**, 133-144 (2020). https://doi.org/10.1016/j.carbon.2019.12.072
10. W. Xu, R. Zhao, Q. Li, B. Sun, J. Wu et al., Overall water splitting on the NiS/NiS_2_ heterostructures featuring self-equilibrium orbital occupancy. Adv. Energy Mater. **13**, 2300978 (2023). https://doi.org/10.1002/aenm.202300978
11. Y. Cheng, L. Zhang, S. Wang, S. Wang, C. Deng et al., 2 A cm^−2^ level large-scale production of hydrogen enabled by constructing higher capacity of interface “electron pocket”. ACS Nano **17**(16), 15504-15515 (2023). https://doi.org/10.1021/acsnano.3c01720
12. Y. Lu, Z. Zhao, X. Liu, X. Yu, W. Li et al., Reconstructed monolithic RuNi heterostructure enables hydrogen production from alkaline seawater at industrial current density. Adv. Sci. **12**, e10916 (2025). https://doi.org/10.1002/advs.202510916
13. L. Yu, L.B. Wu, B. McElhenny, S.W. Song, D. Luo et al., Ultrafast room-temperature synthesis of porous S-doped Ni/Fe (oxy)hydroxide electrodes for oxygen evolution catalysis in seawater splitting. Energy Environ. Sci. **13**, 3439-3446 (2020). https://doi.org/10.1039/D0EE00921K
14. Q. Wen, K. Yang, D. Huang, G. Cheng, X. Ai et al., Schottky heterojunction nanosheet array achieving high-current-density oxygen evolution for industrial water splitting electrolyzers. Adv. Energy Mater. **11**, 2102353 (2021). https://doi.org/10.1002/aenm.202102353
15. N. Yao, G. Wang, H. Jia, J. Yin, H. Cong et al., Intermolecular energy gap-induced formation of high-valent cobalt species in CoOOH surface layer on cobalt sulfides for efficient water oxidation. Angew. Chem. Int. Ed. **61**, e202117178 (2022). https://doi.org/10.1002/anie.202117178
16. T.Y. Dai, X. Zhang, M.Z. Sun, B.L. Huang, N. Zhang et al., Uncovering the promotion of CeO_2_/CoS_1.97_ heterostructure with specific spatial architectures on oxygen evolution reaction. Adv. Mater. **33**(42), 2102593 (2021). https://doi.org/10.1002/adma.202102593
17. N. Zhang, Y. Hu, L. An, Q. Li, J. Yin et al., Surface activation and Ni-S stabilization in NiO/NiS_2_ for efficient oxygen evolution reaction. Angew. Chem. Int. Ed. **61**, e202207217 (2022). https://doi.org/10.1002/anie.202207217
18. W. Wang, Y. Yang, Y. Zhao, S.Z. Wang, X.M. Ai et al., Multi-scale regulation in S, N co-incorporated carbon encapsulated Fe-doped Co_9_S_8_ achieving efficient water oxidation with low overpotential. Nano Res. **15**, 872-880 (2022). https://doi.org/10.1007/s12274-021-3568-8
19. J. Lin, P. Wang, H. Wang, C. Li, X. Si et al., Defect-rich heterogeneous MoS_2_/NiS_2_ nanosheets electrocatalysts for efficient overall water splitting. Adv. Sci. **6**(14), 1900246 (2019). https://doi.org/10.1002/advs.201900246
20. X. Zhu, J. Dai, L. Li, D. Zhao, Z. Wu, Z. Tang, L. Ma, S. Chen, Hierarchical carbon microflowers supported defect-rich Co_3_S_4_ nanoparticles: an efficient electrocatalyst for water splitting. Carbon **160**, 133-144 (2020). https://doi.org/10.1016/j.carbon.2019.12.072
21. H. Wu, Y. Zhang, B. Liu, J. Liu, L. Zhao et al., Mesoporous Co-MoS_2_ with sulfur vacancies: a bifunctional electrocatalyst for enhanced water-splitting reactions in alkaline media. J. Colloid Interface Sci. **684**, 158-169 (2025). https://doi.org/10.1016/j.jcis.2025.01.017
22. X. Zhu, Z. Ji, W. Wan, Y. Zhu, X. Lang, Q. Jiang, Vacancy-rich heterogeneous MnCo_2_O_4.5_@NiS electrocatalyst for highly efficient overall water splitting. J. Colloid Interface Sci. **678**, 878-884 (2025). https://doi.org/10.1016/j.jcis.2024.09.005
23. M. Xi, A.R. Chen, L.F. Yang, Y.Y. Long, H. Zhang et al., Effective hydrogen evolution enabled by heterogeneous interface engineering in bimetallic sulfide with MoNi alloy. Rare Met. **44**(5), 3094-3106 (2025). https://doi.org/10.1007/s12598-024-03163-0
24. P. Luo, H. Zhang, L. Liu, Y. Zhang, J. Deng et al., Targeted synthesis of unique nickel sulfide (NiS, NiS_2_) microarchitectures and the applications for the enhanced water splitting system. ACS Appl. Mater. Interfaces **9**, 2500-2508 (2017). https://doi.org/10.1021/acsami.6b13984
25. L. Jia, G. Du, D. Han, Y. Hao, W. Zhao et al., Ni_3_S_2_/Cu-NiCo LDH heterostructure nanosheet arrays on Ni foam for electrocatalytic overall water splitting. J. Mater. Chem. A **9**(48), 27639-27650 (2021). https://doi.org/10.1039/D1TA08148A
26. Z.J. Zhai, C. Li, L. Zhang, H.C. Wu, L. Zhang et al., Dimensional construction and morphological tuning of heterogeneous MoS_2_/NiS electrocatalysts for efficient overall water splitting. J. Mater. Chem. A **6**(21), 9833-9838 (2018). https://doi.org/10.1039/C8TA03304H
27. J. Hou, B. Zhang, Z. Li, S. Cao, Y. Sun et al., Vertically aligned oxygenated CoS_2_-MoS_2_ heteronanosheet architecture from polyoxometalate for efficient and stable overall water splitting. ACS Catal. **8**(5), 4612-4621 (2018). https://doi.org/10.1021/acscatal.8b00668
28. F. Du, L. Shi, Y. Zhang, T. Li, J. Wang et al., Foam-like Co_9_S_8_/Ni_3_S_2_ heterostructure nanowire arrays for efficient bifunctional overall water-splitting. Appl. Catal. B **253**, 246-252 (2019). https://doi.org/10.1016/j.apcatb.2019.04.067
29. F. Li, R.C. Xu, Y.M. Li, F. Liang, D.F. Zhang et al., N-doped carbon coated NiCo_2_S_4_ hollow nanotube as bifunctional electrocatalyst for overall water splitting. Carbon **145**, 521-528 (2019). https://doi.org/10.1016/j.carbon.2019.01.065
30. Z. Ma, H. Meng, M. Wang, B. Tang, J. Li, X. Wang, Porous Ni-Mo-S nanowire network film electrode as a high-efficiency bifunctional electrocatalyst for overall water splitting. ChemElectroChem **5**(2), 335-342 (2018). https://doi.org/10.1002/celc.201700965
31. C. Jin, P. Zhai, Y. Wei, Q. Chen, X. Wang et al., Ni(OH)_2_-templated synthesis of ultrathin Ni_3_S_2_ nanosheets as a bifunctional electrocatalyst for overall water splitting. Small **17**(33), 2102097 (2021). https://doi.org/10.1002/smll.202102097
32. C. Ren, Y.J. Chen, L.Z. Du, Q. Wang, L.G. Li, G.H. Tian, Hierarchical CuCo_2_S_4_ nanoflake arrays grown on carbon cloth: a remarkable bifunctional electrocatalyst for overall water splitting. ChemElectroChem **8**(6), 1134-1140 (2021). https://doi.org/10.1002/celc.202100247
33. K. Feng, R. Song, J. Xu, Y. Chen, C. Lu et al., The S-Fe(Ni) sub-surface active sites for efficient and stable overall water splitting. Appl. Catal. B **325**, 122365 (2023). https://doi.org/10.1016/j.apcatb.2023.122365
34. M. Ali, M. Wahid, K. Majid, Binary Co_9_S_8_-NiCo_2_S_4_ anchored on N-doped rGO backbone as an efficient bifunctional and durable heterocatalyst for overall water splitting in alkaline medium. New J. Chem. **47**(29), 13888-13902 (2023). https://doi.org/10.1039/D3NJ01470C
35. Z. Wan, Y. Zhang, Q. Ren, X. Li, H. Yu et al., Interface engineering of NiS/NiCo_2_S_4_ heterostructure with charge redistribution for boosting overall water splitting. J. Colloid Interface Sci. **653**, 795-806 (2024). https://doi.org/10.1016/j.jcis.2023.09.117
36. N.S. Gultom, C.H. Li, D.H. Kuo, M.Z. Silitonga, Multiphase Fe-doped Ni_3_S_2_/MoO_x_ electrocatalyst prepared by facile one-step hydrothermal method for full-cell water splitting: effect of Mo on physical and electrochemical properties. Appl. Catal. B **353**, 124100 (2024). https://doi.org/10.1016/j.apcatb.2024.124100
37. X. Dai, Z. Tang, X. Yan, S. Tao, S. Wang et al., In-situ sulfuration of Ni(OH)_2_ to heterostructured Ni_3_S_2_/Ni(OH)_2_@Ni catalyst for efficient water splitting. Chem. Asian J. **20**(3), e202401190 (2025). https://doi.org/10.1002/asia.202401190
38. J. Yu, N. Zhang, J. Li, H. Sun, X. Gu et al., Self-supported NiCo_2_S_4_@Ce-NiFe LDH/CeO_2_ nanoarrays for electrochemical water splitting. Inorg. Chem. **64**(18), 8971-8980 (2025). https://doi.org/10.1021/acs.inorgchem.5c00289
39. M.S.N., S. Gupta, V. Shvalya, M. Kosicek, J. Zavasnik, U. Cvelbar, Advancing oxygen evolution catalysis with dual-phase nickel sulfide nanostructures. Energy Fuels **39**(2), 1375-1383 (2025). https://doi.org/10.1021/acs.energyfuels.4c05182
40. W. Fan, J. Hu, J. Li, P. Tao, Y. Liu, Y. Zhou, Y. He, An electrodeposited ReS_2_/Ni_3_S_2_ heterostructure on nickel foam as a bifunctional electrocatalyst for overall water splitting. Electrochim. Acta **536**, 146827 (2025). https://doi.org/10.1016/j.electacta.2025.146827
41. P. Wang, X. Han, P. Bai, J. Mu, Y. Zhao et al., Utilizing an electron redistribution strategy to inhibit the leaching of sulfur from CeO_2_/NiCo_2_S_4_ heterostructure for high-efficiency oxygen evolution. Appl. Catal. B **344**, 123659 (2024). https://doi.org/10.1016/j.apcatb.2023.123659
42. P. Sharma, L. Khandare, A. Saha, N.B. Chaure, A. Yengantiwar, Growth of Mo-doped Ni_3_S_2_ nanorods array for superior overall water splitting reaction. Int. J. Hydrogen Energy **141**, 729-737 (2025). https://doi.org/10.1016/j.ijhydene.2025.04.479
43. Z. Yang, X. Wu, W. Tan, C. Li, S. Shao, X. Meng, Bilayer heterostructured Ni_3_S_2_@Ta-NiFe LDH cross-linked nanosheets for efficient oxygen evolution reaction. ACS Appl. Mater. Interfaces **17**(32), 45764-45773 (2025). https://doi.org/10.1021/acsami.5c10243
44. Q. Dong, Q. Zhong, J. Zhou, Y. Li, Y. Wang et al., Construction of heterostructured Ni_3_S_2_@V-NiFe(III) LDH for enhanced OER performance. Molecules **29**(24), 6018 (2024). https://doi.org/10.3390/molecules29246018
